# Structurally diverse secondary metabolites from the dung-inhabiting fungus *Botryotrichum murorum*

**DOI:** 10.1038/s41598-026-52958-x

**Published:** 2026-05-14

**Authors:** Esteban Charria-Girón, Yong-Yue Liu, Frank Surup, Yasmina Marin-Felix

**Affiliations:** 1 Department Microbial Drugs, Helmholtz Centre for Infection Research (HZI), Centre for Infection Research (DZIF), Partner Site Hannover/Braunschweig, Inhoffenstrasse 7, 38124 Braunschweig, Germany; 2 Bioinformatics Group, Wageninen University & Research, Droevendaalsesteeg 1, Wageningen, 6708 PB The Netherlands; 3 Institute of Microbiology, Technische Universität Braunschweig, Spielmannstraße 7, 38106 Braunschweig, Germany

**Keywords:** Biochemistry, Chemical biology, Chemistry, Computational biology and bioinformatics, Drug discovery, Microbiology

## Abstract

A combination of confidence-aware MS/MS-based metabolomics and conventional chemical screening approaches was employed to investigate the metabolome of *Botryotrichum murorum* DSM 113281, isolated from tortoise dung. The metabolomic survey revealed a high degree of chemical diversity; however, low production rates necessitated large-scale solid-state cultivation to purify the major constituents. This approach led to the discovery of tortoisellide A (**1**), which features a rare polyketide γ-lactol scaffold, an unprecedented thio-substituted analogue **2** of the 14-membered bis-macrolactone antibiotic grahamimycin A, alongside the known sesquiterpenoid eremophilane cryptosphaerolide (**3**) and isocochliodinol (**4**). All planar structures were elucidated by HR-MS and NMR analysis. Tortoisellide A (**1**) displayed pronounced tautomerism at its hemiacetal carbon C–23, manifesting in the duplication of most NMR signals. Mosher ester analysis supported the assignment of the 2’*S* configuration of compound **2**, while the configurations at C–2 and C–8 were tentatively assigned by analogy to the natural (−)-grahamimycin A1. The previously unassigned side-chain configuration of **3** was determined as 17*S*, 19*S*, 21*S* by NMR chemical shift and *J*-based configurational analyses (Murata’s method) supported by computational modeling. Compounds showed varying degrees of cytotoxicity against mammalian cell lines, with isocochliodinol being most active displaying potency in the nanomolar range. The metabolite diversity of *B. murorum* broadens the chemical repertoire of coprophilous fungi and knowledge on the diversification of secondary metabolism in the Sordariales.

**Supplementary Information:**

The online version contains supplementary material available at 10.1038/s41598-026-52958-x.

## Introduction

Fungal secondary metabolites have long served as an important source of novel antimicrobial molecules and therapeutic agents^1,2^. Several fungal metabolites have inspired or led to the development of commercially used drugs, including antibacterials, antimycotics, cardiovascular drugs, immunosuppressants, immunomodulatory agents, and antiparasitics^3^. Despite the absence of anticancer drugs of fungal origin on the market, contrary to earlier claims related to taxol, which is now known to be exclusively produced by plants^4,5^, semisynthetic illudins are currently in clinical trials for the treatment of castration-resistant metastatic prostate cancer^6^.

Coprophilous fungi, which inhabit or are associated with animal dung, are well-documented as an important source of biologically active molecules^7–9^. Some of these fungi have evolved the capacity to produce secondary metabolites as defense mechanisms in response to the high microbial competition in their environment. Members of the order Sordariales are commonly found in this substrate^10,11^, and many have been recognized as prolific producers of bioactive secondary metabolites^12,13^. For instance, fungi belonging to the family Chaetomiaceae are to date the greatest source of bioactive metabolites within this order, including among others the known chaetoglobosins^14^. These molecules, products of hybrid polyketide synthase non-ribosomal peptide synthetases (PKS-NRPS), are capable of blocking actin polymerization in eukaryotic cells, a process poorly understood at molecular level, stressing the need for comprehensive structure-activity relationship (SAR) studies^15^. Concurrent studies on this fungal group continue to yield promising molecules, such as the potent antifungal dactylfungins produced by *Amesia hispanica*^16^, unusual chetracin-type epithiodiketopiperazines featuring a C-11a′-S-N cross-linkage by another member of *Amesia*^17^, and the arcopilins, biofilm disruptors of *Staphylococcus aureus* biofilms, isolated from the soil-borne fungus *Arcopilus navicularis*^18^.

In our search for novel bioactive metabolites from the Sordariales, the coprophilous fungus *Botryotrichum murorum*, belonging to the Chaetomiaceae, was selected for the study of its secondary metabolome through a confidence-aware Molecular Networking approach in combination with conventional chemical screening. As a result, secondary metabolites from four different biosynthetic classes were isolated and elucidated, corresponding to the new polyketide-derived lactone tortoisellide A (**1**), the thio-substituted derivative **2** of the 14-membered polyketide bis-macrolactone grahamimycin A, the sesquiterpenoid eremophilane cryptosphaerolide (**3**), together with the aminoacid-derived isocochliodinol (**4**). The isolation, structure elucidation, and biological activities of the isolated compounds are presented herein.

## Results and Discussion

### Fungal isolation and identification

**Fig. 1:**
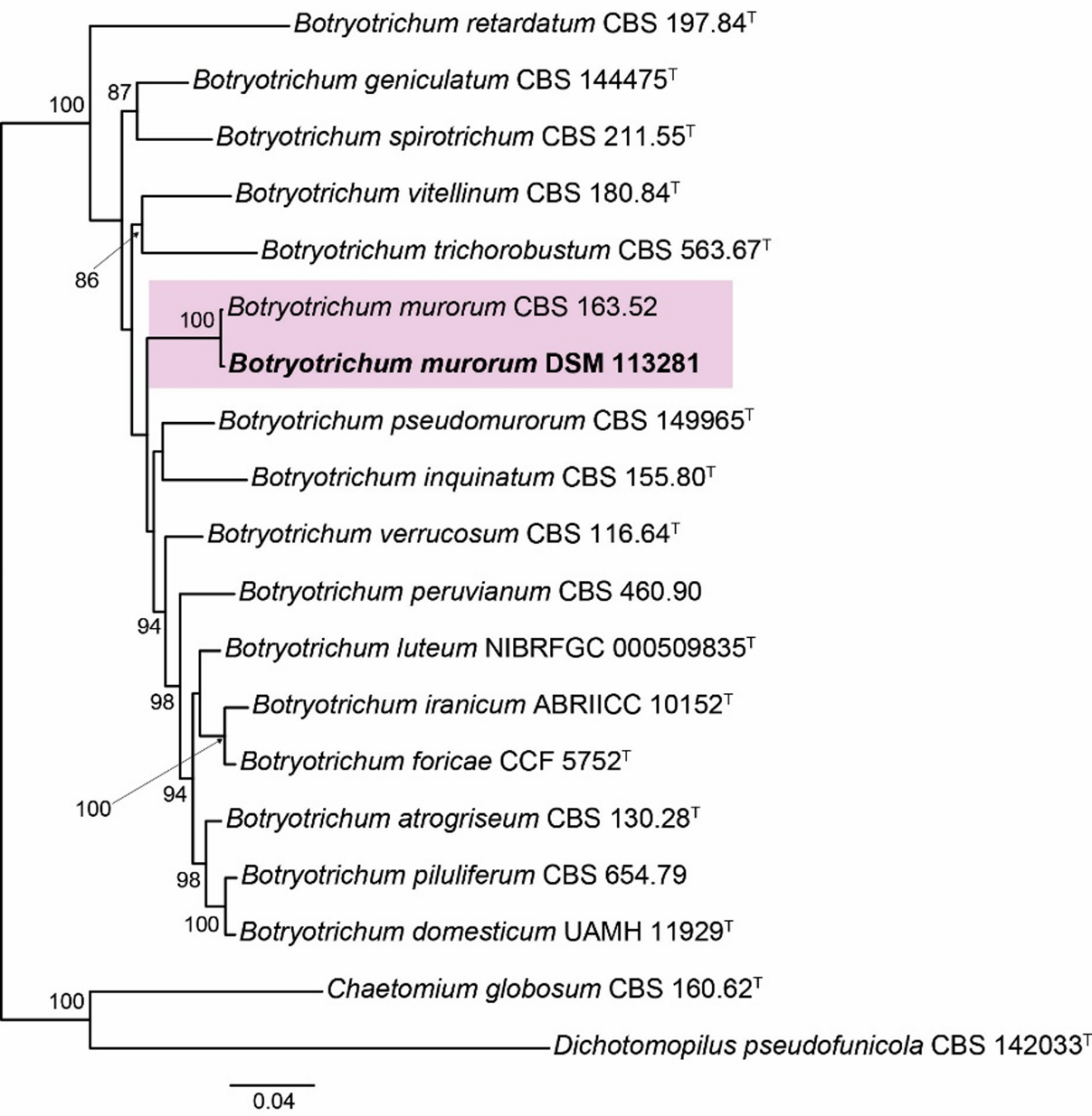
Randomized accelerated maximum likelihood (RAxML) phylogram obtained from the combined sequences of the internal transcribed spacer region (ITS), the nuclear rDNA large subunit (LSU), and fragments of ribosomal polymerase II subunit 2 (*rpb2*) and β-tubulin (*tub2*) genes of species belonging to *Botryotrichum*, with *Chaetomium globosum* CBS 160.62 and *Dichotomopilus pseudofunicola* CBS 142033 as outgroup. Bootstrap support values ≥ 70 are indicated along branches. Branch lengths are proportional to distance. Strain studied in the present study is indicated in **bold**. Type material is indicated with ^T^.

**Fig. 2:**
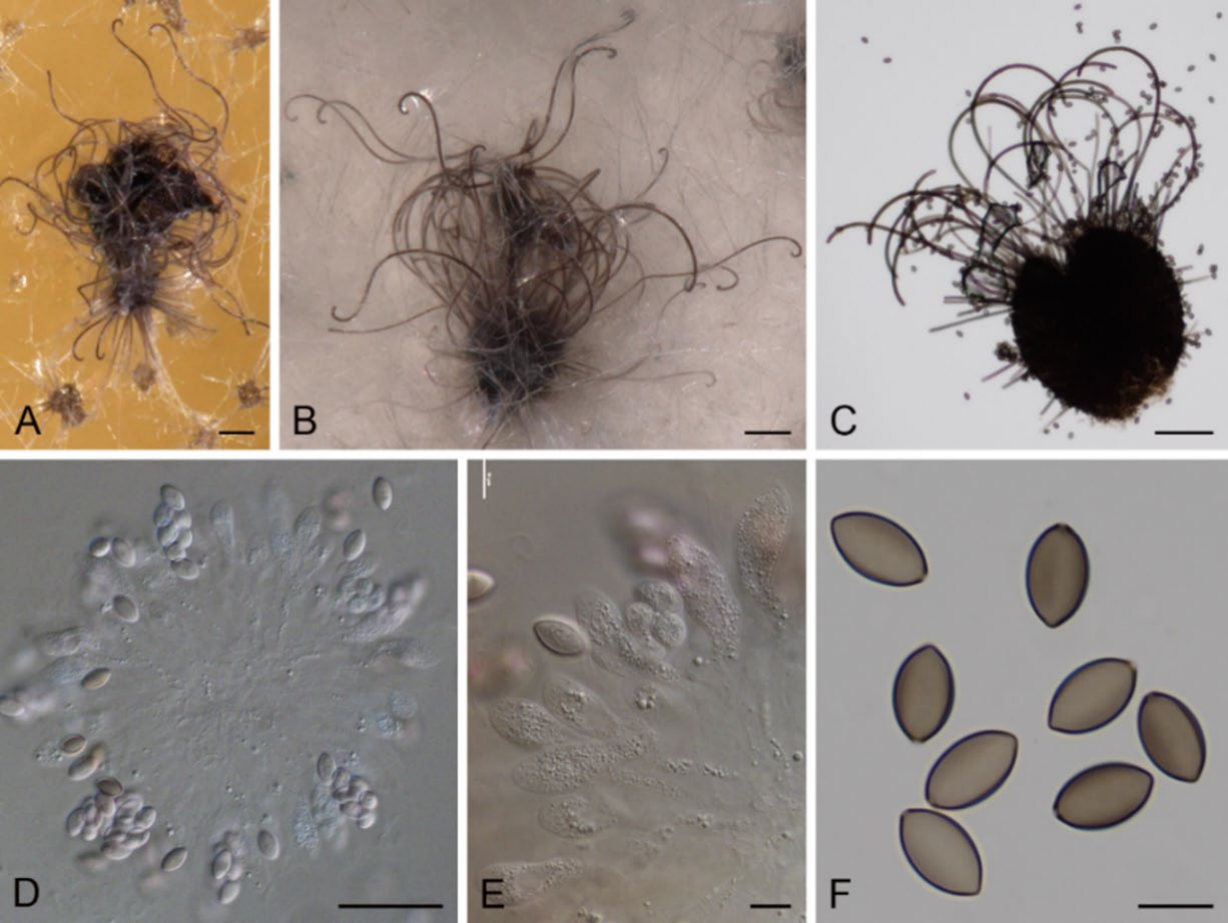
*Botryotrichum murorum* (DSM 113281). A–C. Ascomata. D–E. Asci and ascospores. F. Ascospores. Scale bar: A–C = 100 μm; D = 50 μm; E, F = 10 μm.

The strain DSM 113281 was isolated from tortoise dung and preliminary identified as member of the Chaetomiaceae due to its ostiolate ascomata with hairs surrounding both the ostiole and fruiting body, as well as unicellular, ellipsoidal to fusiform ascospores with an apical germ pore. The phylogenetic tree obtained from the RAxML analysis of the combined dataset, including ITS (575 bp), LSU (541 bp), *rpb2* (525 bp) and *tub2* (772 bp) sequences, is shown in Fig. 1. Our strain was located in a well-supported clade (100 bs) together with the reference strain *B. murorum* CBS 163.52. The morphological characteristics and the size of the reproductive structures matched with those previously described for this species by Wang et al. (Fig. 2)^19^, corroborating the identification at species level.

### Solid Cultivation and MS/MS-based Dereplication

**Fig. 3:**
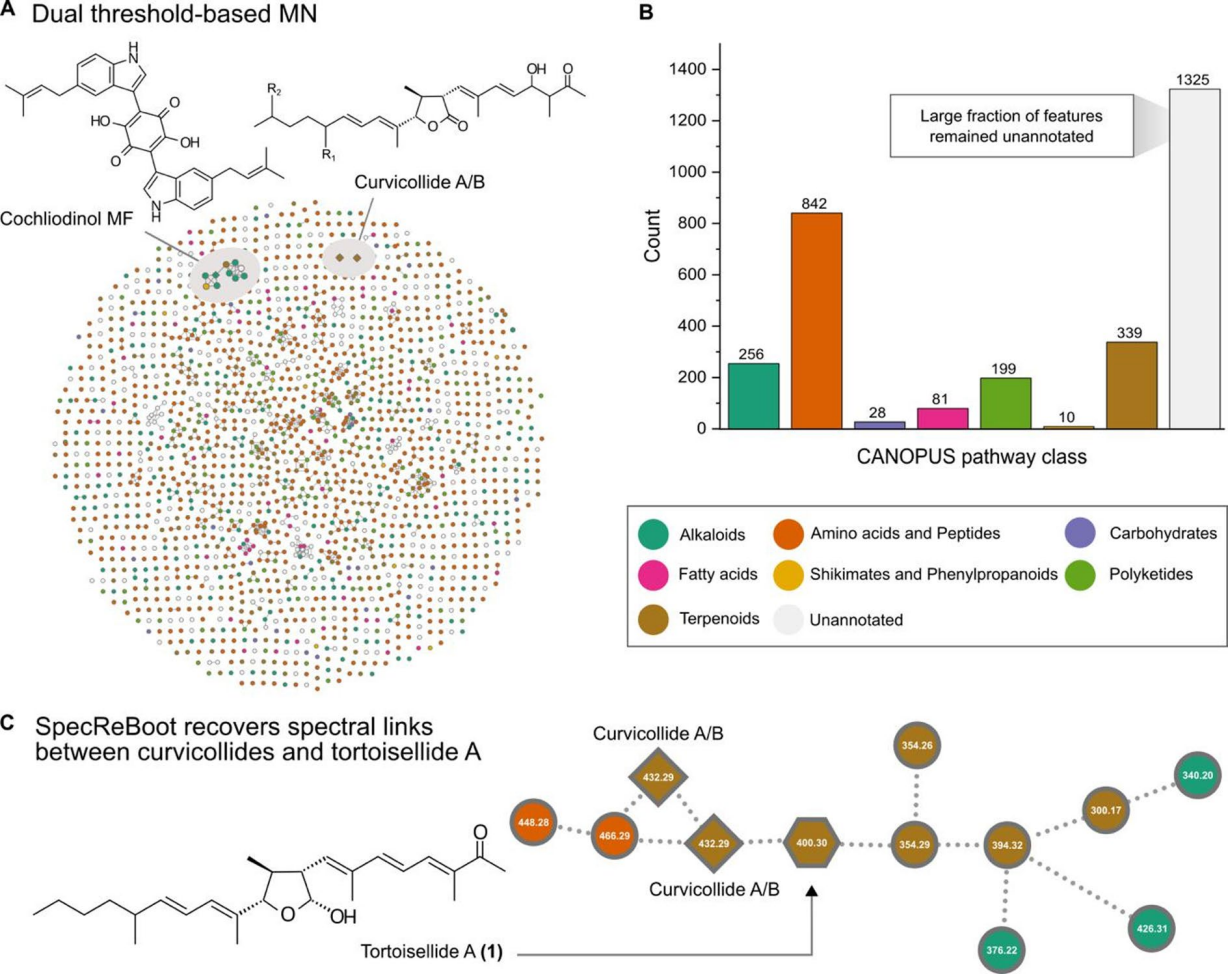
Confidence-aware MN confirms robust spectral links between curvicollides and tortoisellide A (**1**). (A) Dual threshold-based molecular network derived from MS/MS features detected in BRFT solid cultures of *Botryotrichum murorum* DSM 113281, with nodes colored according to CANOPUS pathway-level predictions. Representative annotated features are highlighted, including cochliodinol and curvicollides. (B) Distribution of CANOPUS pathway-level classes among detected MS/MS features. Amino acids and peptides represented the largest annotated group, followed by terpenoids, alkaloids, and polyketides, whereas most features remained without predicted pathway-level annotations. (C) SpecReBoot analysis recovered robust spectral links between curvicollides and tortoisellide A (**1**), revealing relationships that were not evident from the dual threshold-based MN alone. Nodes indicate MS/MS features and are labeled by their neutral masses; dashed edges indicate rescued spectral relationships.

Since only a handful of metabolites have been reported from *Botryotrichum*^19^, we decided to culture the coprophilous fungus *B. murorum* DSM 113281 on solid rice medium (BRFT) to investigate its metabolome using ultrahigh-performance liquid chromatography coupled to diode array detection and ion mobility tandem mass spectrometry (UHPLC-DAD-IM-MS/MS)^20^. After preprocessing the raw data using MetaboScape software, features detected in the medium blank were excluded, and the remaining ones were prioritized for dereplication and further analysis via confidence-aware Molecular Networking using SpecReBoot^21^. Despite the large number of detected signals (MS = 3,374; MS/MS = 3076), only six could be putatively annotated based on accurate mass (mass deviation < 2 mDa), isotope pattern quality (mSigma < 20), and MS/MS spectral matching (MS/MS score > 900) (Table S1). One of these putative annotations suggested that the main compound within the BRFT extracts corresponded to cochliodinol, a compound widely produce by members of the Chaetomiaceae aside from the cultures of *Pyrenopolyporus* (Hypoxylaceae, Xylariales), which makes it a reliable chemotaxonomic marker at the family level^19,22^. Additionally, two distinct features were both annotated as curvicollides A or B, which share the same molecular formula. However, these features appeared as singletons within our dual threshold-based MN, which retained only edges fulfilling two criteria: a mean modified cosine similarity above 0.7 and edge support above 0.5 (Fig. 3A).

To expand our knowledge on the chemical space produced by *B. murorum* after BRFT cultivation, we predicted *de novo* the chemical classes for those detected MS/MS features using the CANOPUS tool within SIRIUS software^23,24^. This provided further insights into the structural diversity, being evident that the main compound classes observed in the BRFT extracts belonged to the amino acids and peptides, terpenoids, and alkaloids classes, while other such as shikimates and phenylpropanoids or carbohydrates were rather absent or underrepresented in the samples. Notably, for 1,325 nodes there were not compound class predictions by CANOPUS, which overall accounted for ~ 43% of the total number of detected features at the MS/MS level. Since most of the observed chemical space remained obscure to us after our dereplication efforts, we embarked on the targeted isolation of the major metabolites present in the crude extract.

### Structure elucidation of major metabolites

**Fig. 4:**
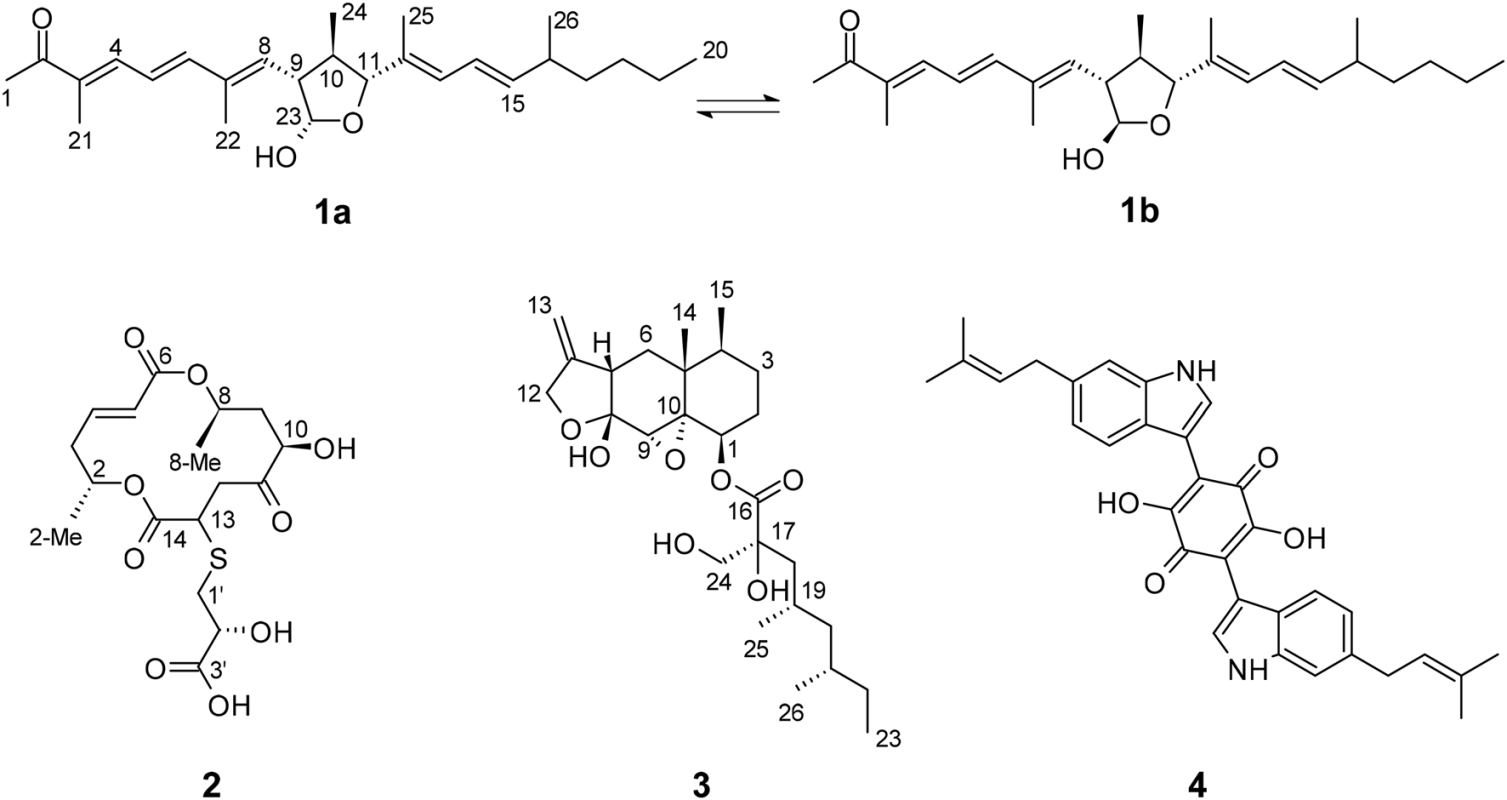
Chemical structures of isolated metabolites from the BRFT cultures of *Botryotrichum murorum* DSM 113281.

Fractionation of the crude extract obtained from the scaled-up cultivation of *B. murorum* in BRFT medium (50 flasks) resulted in the isolation of the main compounds **1**–**4** (Fig. 4). Compound **1** was isolated as a colorless oil; its quasimolecular ion cluster at *m/z* 401.3046 [M + H]^+^ indicated the molecular formula to be C_26_H_40_O_3_, implying 7 degrees of unsaturation. Signals in ^1^H and^13^C NMR spectra (Table 1) did not match this number, since at least 40 carbon signals were detected, suggesting the presence of two slowly interconverting tautomers on the NMR timescale, leading to signal duplication. The largest chemical shift difference was observed for C–23, indicating the difference is located at this position. Consequently, the structures of both isomers had to be elucidated in parallel. COSY and HMBC NMR data established the planar structures of the two tautomers **1a**/**1b** in a straightforward way (Figure S1), revealing a central 2-hydroxy-3,5-bisacyl-4-methyloxolane connected to two partially unsaturated carbon chains. Subsequently, the relative stereochemistry of the C–9/C–10/C–11 segment was assigned based on ROESY correlations, with the strong cross-peak between H–9 and H–11 being particularly diagnostic. In contrast, the configuration at C–23 could not be unambiguously determined from ROESY data alone. Thus, C,H coupling constants were determined by a HSQC-Hecade NMR experiment. Most couplings were highly similar, but ^2^*J*_H9a, C23a_ = −2.5 Hz and ^2^*J*_H9b, C23b_ = −9.4 Hz as the key difference define the configuration of **1a** and **1b**^25^. Accordingly, compound **1** was named tortoisellide A, in reference to the animal from which the producing strain was isolated in its dung. This irregularly assembled polyketide resembles the scaffolds of the annotated curvicollides and also of fusaequisin A, which are intriguing structures due to the non-aldol stereotriad within their lactone ring. The former were reported from the sclerotium-colonizing fungus *Pseudoechria curvicolla* (syn. *Podospora curvicolla*), and the latter from *Fusarium equiseti*^26,27^. The complexity and unusual chemistry of these metabolites have led to the proposal that they arise from condensation of two distinct polyketide subunits and have motivated different total synthesis campaigns to clarify their full structural assigment^28,29^. Notably, compound **1** shares the same C26 scaffold with these molecules, however, the fewer oxidative modifications suggest that tortoisellide A might be an earlier product in the biosynthesis of the more complex curvicollides. This hypothesis is consistent with our dereplication efforts, in which two features in our MN with molecular formulae C_26_H_40_O_5_ were annotated as curvicollide A/B, suggesting a shared biosynthetic origin. Although these curvicollide-related features appeared as singletons in the dual threshold-based MN, by further rescuing edges with low spectral similarity but exhibiting a support value over 0.5, SpecReBoot recovered links between them and compound **1**, further supporting their chemical relatedness (Fig. 3C). Moreover, the isolation of **1** as an inseparable mixture of isomers raises the possibility that the observed interconversion reflects late-stage, spontaneous hemiacetal formation, implying that only one conformer may correspond to the genuine natural product. Future dedicated studies will be required to clarify this point and shed light on the unusual polyketide chemistry of these γ-lactol metabolites.

**Table 1:** NMR data (700 MHz, DMSO-*d*_6_) of **1**.

pos	Major epimer 1a		Minor epimer 1b	
	δ_C_, mult.	δ_H_, mult.	δ_C_, mult.^a^	δ_H_, mult. ^a^
1	25.6, CH_3_	2.30, d (2.2)		
2	198.8, C			
3	134.8, C			
4	140.2, CH	7.25, t (12.1)	140.0, CH	
5	122.7, CH	6.53 (m)	123.0, CH	6.55, m
6	145.2, CH	6.80, d (12.2)	144.7, CH	6.78, d (12.2)
7	135.6, C		136.3, C	
8	135.1, CH	5.74, d (9.9),	136.4, CH	5.71, d (9.7)
9	51.1, CH	2.74, ddd (11.3, 9.9, 4.6 Hz)	54.9, CH	2.68, td (10.1, 5.2)
10	40.5, CH	2.00, m	44.1, CH	1.80, m
11	91.4, CH	3.86, d (9.7)	89.0, CH	4.04, d (9.6)
12	134.6, C		133.4, C	
13	126.9, CH	5.97, t, (9.7)	127.1, CH	
14	124.3, CH	6.20, m	124.0, CH	6.23, m
15	140.5, CH	5.57, dd (14.6, 7.9)	140.8, CH	5.60, dd (14.6, 7.9)
16	36.3, CH	2.17, m		
17	36.2, CH_2_	1.27, m		
18	29.0, CH_2_	1.22, m		
19	22.3, CH_2_	1.26, m		
20	14.0, CH_3_	0.85, t (7.1)		
21	11.4, CH_3_	1.83, br s		
22	12.9, CH_3_	1.86, br s	12.8, CH_3_	
23	97.0, CH	5.15, d (4.6), OH: 6.31 (br s)	102.0, CH	5.07, d (5.2)
24	14.1, CH_3_	0.80, d (6.7)	14.2, CH_3_	0.82, d (6.7)
25	11.60, CH_3_	1.73, s	11.57, CH_3_	1.67, s
26	20.4, CH_3_	0.97, d (6.7)		

^a^ only signals different from **1a** are shown for **1b**.

For compound **2**, the molecular formula C_17_H_24_O_9_S was determined based on HR-ESI-MS data, indicating six degrees of unsaturation. The presence of a sulfur atom was supported by the isotopic pattern in the sodium adduct ion peak at *m/z* 517.2237 [M + Na]^+^, and the characteristic NMR shift values for the sulfated methylene of CH_2_−1’ (*δ*_C_ 35.5, *δ*_H_ 2.93 and 2.77) and methine CH-9 (*δ*_C_ 36.4, *δ*_H_ 2.87). Moreover, ^1^H and HSQC (Table 2) spectra account for signals for two olefinic methines, four oxy- and one thiomethine as well as four methylenes. The^13^C data obtained from HMBC data revealed the presence of a single ketone and three carboxyl atoms. Moieties spanning CH–4/CH_2_–5/CH–6/CH_3_–16, CH–9/CH_2_–10, CH–12/CH_2_–13/CH–14/CH_3_–15 and CH_2_–1’/CH–2’ were assembled by COSY correlations. Additionally, HMBC correlations confirmed that compound **2** contains a grahamimycin A scaffold connected to a (2-hydroxy-3-carboxypropyl)thio substituent, even though the latter was not detected within the chemical space observed for *B. murorum*. However, the HMBC data alone did not allow an unambiguous distinction between attachment of the side chain at C–12 or C–13. This ambiguity was resolved by ROESY data, which showed clear correlations between H–10 and both methylene protons at C–12, indicating their spatial proximity. These correlations are consistent with the side chain being attached at C–13, whereas an attachment at C–12 would not account for the observed ROESY cross-peaks.

**Table 2:** NMR data (700 MHz, DMSO-*d*_6_) of **2**.

pos	δ_C_, mult.	δ_H_, mult.	COSY	HMBC	ROESY
2	69.7, CH	4.24, m	2-Me, 3b	2Me, 3, 4,14	2-Me, 3a, 4 > > 13
3	38.6, CH_2_	2.48, m 2.20, m	3b, 4 2, 3a, 4	2, 4, 5 2, 4, 5, 2-Me	3b, 2-Me > 2, 4,5 3a, 2-Me > 2, 4
4	144.6, CH	6.56, ddd (15.8,9.0,6.9)	3a, 3b, 5	2, 3, 5, 6	2, 3a, 3b
5	124.4, CH	5.59, d (15.8)	4	2, 3, 4, 6	3a, 3b, 13
6	164.5, C				
8	65.5, CH	5.03, m	9a, 8-Me	6, 8-Me, 9, 10	9b, 10 > 12
9	39.2, CH_2_	2.16, m 1.84, ddd (14.6,7.2,1.5)	9b, 10 9a, 10	8, 10, 8-Me 10, 11	9b, 10 9a, 10, 8-Me > 8
10	72.6, CH	4.08, dd (7.2,1.5)	9a, 9b	8, 9, 11	8-Me, 9a, 9b > 12a, 12b
11	210.1, C				
12	41.8, CH_2_	2.99, dd (20.2,4.5) 2.93, m	12b, 13 12a, 13	11, 13, 14 11, 13, 14	10, 13 13
13	39.0, CH	3.80, dd (6.0,4.5)	12a, 12b	11, 12, 14, 1’	1’a, 1’b, 5
14	172.5, C				
2-Me	20.2, CH_3_	1.28, d (6.3)	2	2, 3	2, 3a, 3b
8-Me	20.0, CH_3_	1.14, d (6.5)	8	8, 9	8, 9a, 9b > 10
1’	35.5, CH_2_	2.93, m 2.77, dd (13.3,7.3)	1’b, 2’ 1’a, 2’	13, 2’, 3’ 13, 2’, 3’	1’b, 2’ 13, 1’a, 2’
2’	69.8, CH	4.03, m	1’a, 1’b	3’	1’a, 1’b
3’	173.8, C				

**Fig. 5:**
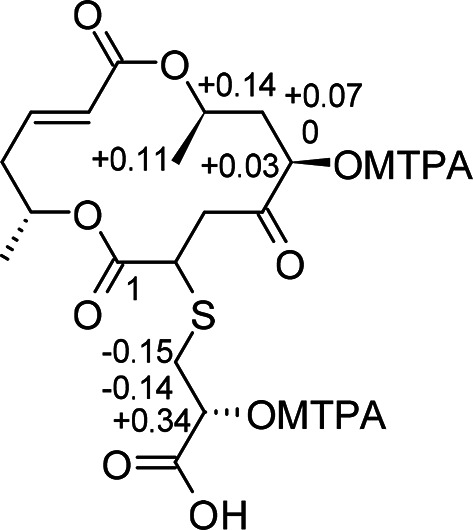
*Δδ*^SR^ values for MPTA esters of thio-grahamimycin **2** diagnostic for 12*R*,2’*S* configuration.

The absolute configuration of natural (−)-grahamimycin A1 had previously been established by total synthesis^30^. Compound **2** showed a negative optical rotation ([α]^20^_D_ −43, MeOH), consistent with the natural (−)-grahamimycin series and opposite to the reported synthetic (+)-enantiomer. Therefore, the configurations at C–2 and C–8 were tentatively assigned by analogy. This assignment is further supported by the fact that the stereochemistry of methyl-bearing centers in macrolides of this class is set during chain assembly by the nascent polyketide and should be conserved whenever the PKS processes the same substrate, whereas the stereochemistry of post-PKS hydroxylations can diverge. Consistent with this, colletodiol, a structurally related macrodiolide from *Diplogelasinospora grovesii* (Diplogelasinosporaceae, Sordariales)^31^, displays the same configuration at the corresponding C–2 and C–8, the biosynthetically conserved methyl-bearing centers. The configuration at C–2’ was investigated by Mosher ester analysis^32^. Despite poor spectral quality only allowed partial assignments (Fig. 5, Table S2), which were mainly deducted from the HSQC and COSY spectral data, positive *Δδ*^SR^ values for H_3_–15, H–14, H–12a and H–12b confirmed the 10*R* absolute configuration. In addition, the negative *Δδ*^SR^ values for H–1’a and H–1’b indicated a 2’*S* configuration. Thus, the configuration at C–2’ could be assigned as 2’*S*, while the configurations at C–2 and C–8 remain tentative assignments based on biosynthetic analogy and optical rotation data. The configuration at C–12 remains unassigned.

The incorporation of a 2-hydroxy-3-mercaptopropanoic acid moiety has been associated with self-detoxification mechanisms in diverse natural products, such as sulfurated pleurotin derivatives^33^. However, the presence of this structural moiety in the berkeleylactones is essential for the observed biological activity^34^. Within the Sordariales, this metabolite class is uncommon, and to date only colletodiol and its epimer have been reported in *Diplogelasinospora grovesii*, which lack the 2-hydroxy-3-mercaptopropanoic acid thio-substitution^31^. Whether the thio-decoration observed on this macrolide scaffold derives from an enzymatic tailoring step or a spontaneous reaction is yet unclear and warrants further exploration of the biosynthetic diversity of macrolide polyketides within the Sordariales.

Similarly, cryptosphaerolide (**3**) was identified by comparison of its HR-ESI-MS and NMR data with literature values. In particular, ^1^H, ^13^C, COSY, ROESY, HSQC and HMBC NMR data measured in chloroform-*d* were virtually identical (see Table S3, Figures S21–S25). The structure of **3**, including the absolute configuration of the sesquiterpenoid core, had been previously elucidated^35^. However, the stereochemistry of the side chain remained unassigned. To resolve this, we undertook a stereochemical assignment using a combination of NMR-based methods. The large chemical shift difference *Δδ*_H_ = 0.20 ppm of the germinal protons H–20a (*δ*_H_ 1.15 ppm) and H–20b (*δ*_H_ 0.95 ppm) indicated a *syn* configuration of the groups CH_3_–25 and CH_3_–26, according to Breit’s rule^36^. To assign the relative configuration between the oxymethylene CH_2_–24 and methyl CH_3_–25, a *J*-based conformational analysis was performed. Relevant coupling constants were obtained from H and *J*-resolved NMR experiments for H,H coupling constants, and from HSQC–Hecade and *J*-HMBC spectra for H,C coupling constants, respectively.

The interpretation of the data for the C–17/C–18 bond gave clear evidence for a *syn* configuration of the methyl groups CH_3_–25 and CH_3_–26. Coupling constants disprove all conformations of the relative configuration depicted in D-F, and two of the three possible conformations (B & C) to the left. Unfortunately, the interpretation of the data for the C–18/C–19 bond was more difficult, since several of the diagnostic coupling constant values were not significant. However, with interpretation of only the significant coupling constant values of ^3^*J*_H18aH19_ = 4.8 Hz, ^3^*J*_C20H18b_ = 2.9 Hz and ^3^*J*_C25H18b_ = 5.6 Hz it was possible to generate a structure proposal depicted in Figures S3–S6. Other potential rotamers are unlikely to be the actual conformation due to these significant coupling constants. This assignment is further confirmed by a ROESY correlation between methyl group CH_3_–25 and oxymethylene CH_2_–24, which is characteristic for *syn* configurated chains. Taken together, the *J*-resolved analysis yielded the side chain to be 2*R*,4*R*,6*R* or 2*S*,4*S*,6*S* configurated. A rather strong ROESY correlation between CH_2_–24 and CH_3_–14 was observed, which facilitated the transfer of the absolute configuration of the eremophilane central moiety, established by Mosher’s method, to the 2-hydroxy-2-(hydroxymethyl)−4,6-dimethyloctanoic acid side chain. Thus, both possibilities 2*R*,4*R*,6*R* and 2*S*,4*S*,6*S* were calculated together with the known 1*R*,4*S*,5*R*,7*S*,8*R*,9*S*,10*R* configuration with the program ORCA 6.0.0. C–14/C–24 distances of 4.800 Å and 4.137 Å were obtained for 2*R*,4*R*,6*R* and 2*S*,4*S*,6*S* configurations, respectively. Since the observed ROESY cross peak is only in line with a distance of about 4 Å, the absolute configuration was assigned as 2*S*,4*S*,6*S* configuration.

Finally, the molecular formula of the major metabolite in the BRFT extract was initially determined to be C_32_H_32_N_2_O_4_, based on its putative annotation as cochliodinol. However, direct comparison with an authentic standard isolated from a related chaetomiaceous fungus revealed differences in retention time and UV/Vis spectra, suggesting it was a distinct isomer (Figure S7)^16^. After purification, detailed NMR analysis, alongside literature comparison, revealed its identity as isocochliodinol (**4**)^37^.

### Biological properties of metabolites produced by *B. murorum*

All isolated metabolites were evaluated for their antimicrobial and cytotoxic activities (Table 3 and S6). Regrettably, compound **1** was not obtained in sufficient quantity for biological testing. Related molecules such as the curvicollides and fusaequisin A exhibit diverse activities, including antifungal effects (curvicollides), antibacterial properties (fusaequisin A), and inhibition of *Trypanosoma brucei*, the causing agent of African trypanosomiasis, via DNA binding and transcriptional blockade (curvicollide D)^25,26,38,39^. Notably, this latter molecule, isolated from a chaetomiaceous fungus of the genus *Amesia*, despite its name, has a distinct carbon skeleton and oxidation pattern compared with the “original” curvicollides, which suggest the diversification of this biosynthetic pathway within the Chaetomiaceae and other families from the Sordariales. Future studies on the diversity of these metabolites in *Botryotrichum* and *Amesia* or even related taxa will clarify their biological properties, including those of **1**, and determine whether their production is conserved across other members of the family and beyond.

On the other hand, the thio-derivative of grahamimycin A (**2**) showed no antimicrobial activity up to the highest tested concentration (see methods), and only limited cytotoxicity, with IC_50_ values of 56.9 and 47.0 µM against A549 and KB 3.1 cell lines, respectively. The absence of antimicrobial properties for **2** confirms that the incorporation of the 2-hydroxy-3-mercaptopropanoic acid moiety acts as a detoxification mechanism, since the known grahamimycin A has been shown to be active against a wide range of diverse microorganisms^40^. In contrast, cryptosphaerolide (**3**) displayed selective antimicrobial effects, including strong activity against *Bacillus subtilis* and *Staphylococcus aureus*, moderate inhibition of *Mycolicibacterium smegmatis*, and weak antifungal activity against *Mucor hiemalis* and *Rhodotorula glutinis* (Table S6). Compound **3** also exhibited significant cytotoxicity, with IC_50_values of 2.5 and 2.8 µM against the KB 3.1 and L929 cell lines, respectively. Notably, cryptosphaerolide has been reported as an inhibitor of the anti-apoptotic protein Mcl-1, a clinically relevant cancer drug target^35^. However, as happened in this study, its low production yield may have impeded further pharmacological and mechanistic investigations into its therapeutic potential.

Additionally, to compare both cochliodinol isomers, isocochliodinol (**4**) isolated in this study was tested alongside cochliodinol, previously isolated from *Amesia hispanica* (Table S6)^16^. Isocochliodinol showed moderate inhibition of *B. subtilis* and weak activity against *M. hiemalis*, but no activity against other tested strains. In contrast, cochliodinol showed weak activity against *B. subtilis* and *R. glutinis*, but was inactive against *M. hiemalis*. Both compounds displayed cytotoxic effects, though **4** was significantly more potent, ranging from 0.058 to 1.8 µM across all tested cell lines, including submicromolar effects on A431, A549, and MCF-7 cells. The above suggest that positional isomerism significantly influences the antimicrobial and cytotoxic activity of this compound scaffold, offering new insights into the modulation of their biological properties.

**Table 3:** Cytotoxicity of isolated metabolites against mammalian cell lines [half maximal inhibitory concentrations (IC_50_): µM].

Compound	A431	A549	KB 3.1	L929	MCF-7	SK-OV-3	PC-3
**2**	–	56.9	47.0	14.6	–	–	–
**3**	–	–	2.5	2.8	–	–	–
**4**	0.24	0.072	0.058	0.068	0.18	0.24	1.8
**Cochliodinol** ^16^	–	–	5.5	4.9	–	–	–
**Epothilone B**	0.000026	0.000034	0.000027	0.00084	0.000015	0.00013	0.000048

## Conclusion

Motivated by recent taxonomic revisions in the Chaetomiaceae and the hitherto underexplored phylogenetic diversity of its genera, we explored the secondary metabolome of the coprophilous fungus *Botryotrichum murorum*, isolated from tortoise dung. Confidence-aware Molecular Networking and CANOPUS-guided annotation revealed a high degree of putatively novel chemistry or unannotated features, which led us to isolate and characterize the major metabolites produced in BRFT solid cultures and reconcile the chemical diversity of this coprophilous fungus. The resulting secondary metabolites can be classified within distinct biosynthetic classes, highlighting the chemical creativity of *B. murorum* and uncovering unprecedented chemical scaffolds not previously described from the Chaetomiaceae or rarely found in the Sordariales. Among these, we characterized a γ-lactol macrolide pair of isomers that resemble the curvicollides/fusaequisin A family, all with a unique carbon skeleton and oxidative pattern. While curvicollides have been reported from distantly related Sordariales taxa, it remains an open question whether the biosynthetic machinery underlying these scaffolds is broadly distributed across families within the order or reflects more restricted patterns of inheritance such as potential horizontal transfer gene transfer events. Nevertheless, the compounds reported here expand the known chemical space of the Chaetomiaceae and suggest biosynthetic solutions that differ from superficially similar pathways in other lineages in the Sordariales. Despite the chemical novelty of **1**, the biological properties of this novel polyketide macrolide remains obscure to us, which should be addressed by future experimental efforts complemented by genome mining and directed biosynthetic experiments to gain insights into the unusual polyketide chemistry of these molecules, ultimately linking these to their biological role. Similarly, more comprehensive campaigns across the Chaetomiaceae and other sordarialean groups are planned to evaluate the structural and biological diversification of these molecules.

## Methods

### Fungal Isolation and Identification

Strain DSM 113281 was isolated from tortoise dung collected in the Essehof zoo (Lower Saxony, Germany), following the moist chamber method and incubating the sample at room temperature^41^. Fungal colonies were examined up to 2 months under a Zeiss Stemi 508 stereomicroscope (Jena, Germany), and reproductive structures of the fungus were transferred to Petri dishes containing yeast malt agar (YM agar; malt extract 10 g/L, yeast extract 4 g/L, D-glucose 4 g/L, agar 20 g/L, pH 6.3 before autoclaving, with 250 mg/L penicillin and 250 mg/L streptomycin to avoid bacteria, and 50 mg/L ivermectin to avoid mites or nematodes) using a sterile needle.

Fertile fungal structures grown in YM were mounted and measured in lactic acid. Photomicrographs of the structures were obtained with a Keyence (Neu-Isenburg, Germany) VHX-970 F microscope, and a Nikon eclipse Ni compound microscope, using a DS-Fi3 digital camera (Nikon, Tokyo, Japan) and NIS-Elements imaging software v. 5.20.

DNA extraction and PCR amplification were done as Charria-Girón et al.^16^. PCR products were sequenced using Sanger Cycle Sequencing method at Microsynth Seqlab GmbH (Göttingen, Germany), and the consensus sequences were obtained using Geneious^®^ 7.1.9 (http://www.geneious.com)^42^. For the polyphasic identification of the fungus, a phylogenetic analysis was carried out based on the combination of the four loci of our isolate and those of the type and reference strains of taxa belonging to the genus *Botryotrichum* (Table 4). Each locus was aligned separately using MAFFT v. 7 (Katoh et al.^43^), manually optimized using MEGA v. 10.2.4 (Kumar et al.^44^). The four loci were then concatenated using SequenceMatrix (Vaidya et al.^45^), after checking for no conflicts. The Maximum Likelihood (ML) analysis was done on the CIPRES portal (www.phylo.org) using RAxML-HPC BlackBox v8.2.12 with default parameters (Stamatakis^46^), and bootstrap support (bs) ≥ 70% were considered significant. The sequences generated in the present study have been deposited in GenBank (Table 4), and the final alignment is available in the Supplementary Material.

**Table 4:** Our isolate, as well as type and reference strains of the genus *Botryotrichum* included in the phylogenetic study. Strain studied in the present study and sequences generated are indicated in **bold**. ABRIICC: Agriculture Biotechnology Research Institute of Iran culture collections, Karaj, Iran; CBS: Westerdijk Fungal Biodiversity Institute, Utrecht, the Netherlands; CCF: Culture Collection of Fungi, Department of Botany, Faculty of Science, Charles University, Prague, Czech Republic; DSM: German Collection of Microorganisms and Cell Cultures, Braunschweig, Germany; NIBR: Culture center of National Institute of Biological Resources, Incheon, Republic of Korea; UAMH: Centre for Global Microfungal Biodiversity at the University of Toronto, Toronto, Canada. ^T^ indicates type material.

Species	Strain	GenBank accession number				References
		ITS	LSU	rpb2	tub2	
*Botryotrichum atrogriseum*	CBS 130.28^T^	KX976589	KX976714	KX976813	KX976931	Wang et al.^47^
*B. domesticum*	UAMH 11929^T^	MH899168	MH899169	MH899171	MH899172	Schultes et al.^48^
*B. foricae*	CCF 5752^T^	LR584032	LR584033	-	LR584034	Crous et al.^49^
*B. geniculatum*	CBS 144475^T^	MZ334719	MZ351422	MZ342972	MZ343011	Wang et al.^50^
*B. inquinatum*	CBS 155.80^T^	MK919289	MK919289	MK919345	MK919403	Wang et al.^50^
*B. iranicum*	ABRIICC 10152^T^	MN134583	MN134576	-	MN128435	Alidadi et al.^51^
*B. luteum*	NIBRFGC 000509835^T^	LC731694	LC731695	LC731696	LC731697	Ryu et al.^52^
*B. murorum*	CBS 163.52	KX976591	KX976716	KX976815	KX976933	Wang et al.^47^
	**DSM 113281**	**PX442094**	**PX442095**	**PX508860**	**PX508861**	**Present study**
*B. peruvianum*	CBS 460.90	KX976595	KX976720	KX976819	KX976937	Wang et al.^47^
*B.piluliferum*	CBS 654.79	KX976597	KX976722	KX976821	KX976939	Wang et al.^47^
*B. pseudomurorum*	CBS 149,965^T^	OZ001674	OZ001675	OZ001714	OZ001715	Sastoque et al.^53^
*B retardatum*	CBS 197.84^T^	MH861728	-	MZ342980	MZ343019	Vu et al.^54^, Wang et al.^50^
*B. spirotrichum*	CBS 211.55^T^	KX976601	KX976726	KX976825	KX976943	Wang et al.^47^
*B. trichorobustum*	CBS 563.67^T^	-	MZ351420	MZ342988	MZ351420	Wang et al.^50^
*B. verrucosum*	CBS 116.64^T^	LT993567	LT993567	-	LT993648	Wang et al.^55^
*B. vitellinum*	CBS 180.84^T^	MZ334725	MZ351421	MZ342979	MZ343018	Wang et al.^50^
*Chaetomium globosum*	CBS 160.62^T^	KT214565	KT214596	KT214666	KT214742	Wang et al.^56^
*Dichotomopilus pseudofunicola*	CBS 142033^T^	KX976668		KX976870	KX977010	Wang et al.^47^

### General Experimental Procedures

The 1D- and 2D‐ nuclear magnetic resonance (NMR) spectra of the isolated compounds were recorded with an Avance III 700 spectrometer with a 5 mm TXI cryoprobe (Bruker, ^1^H NMR: 700 MHz, ^13^C: 175 MHz, Billerica, MA, USA) and an Avance III 500 (Bruker, ^1^H NMR: 500 MHz, ^13^C: 125 MHz, Billerica, MA, USA) spectrometer, respectively. The chemical shifts δ were referenced to the solvents DMSO‐*d*_6_ (^1^H, δ = 2.50; ^13^C, δ = 39.51). Optical rotations were measured with an MCP 150 circular polarimeter at 20 °C (Anton Paar, Graz, Austria) and UV/Vis spectra with a UV‐2450 spectrophotometer (Shimadzu, Kyoto, Japan). The optical rotation was obtained in MeOH and the UV/Vis spectra were measured in ACN.

### Untargeted Metabolomics Analysis

Each sample was analyzed at a concentration of 450 µg/mL on a Dionex Ultimate 3000RS (Thermo Scientific, Bremen, Germany), using a Kinetex C18 (1.7 μm, 21 × 150 mm, 100 Å) with a sample injection volume of 2 µL. The mobile phase consisted of A (H_2_O + 0.1% formic acid) and B (ACN + 0.1% formic acid) with a constant flow rate of 0.3 mL/min. The gradient began with 1% B for 0.5 min, increasing to 5% B in 1 min, then to 100% B in 19 min and holding at 100% B for 5 min. DAD-UV data was recorded at 190–600 nm and the temperature of the column was set at 40 °C. MS spectra were collected using a timsTOF Pro mass spectrometer (Bruker Daltonics, Bremen, Germany) with the following parameters: tims ramp time 100 ms, spectra rate 9.52 Hz, PASEF on, cycle time 320 ms, MS/MS scans 2, scan range (*m*/*z*, 100 − 1800 Da; 1/k0, 0.55–2.0 V.s/cm^2^). The untargeted profiling data and the measurement of standards was acquired in both ESI positive and negative ion modes.

Data was first pre-processed with MetaboScape^®^2022 (Bruker Daltonics, Bremen, Germany) in the range of 0.5 to 25 min. First, the mass spectrometry features with a coefficient of variance higher than 80% between the three biological replicates were excluded from the analysis, as well as features present in the medium blank. The resulting features were dereplicated against our in-house spectral database of authentic standards from characteristic fungal metabolites. Feature-based molecular networks were constructed using SpecReBoot as follows: first, the exported MGF file was preprocessed using DEFAULT_FILTERS and CLEAN_PEAKS from matchms for general spectral cleaning. The cleaned MGF file was then used as input for the SpecReBoot bootstrapping workflow^21^. Spectral similarities were calculated using the modified cosine score implemented in FlashSimilarity, with a fragment mass tolerance of 0.02 Da, 100 bootstrap replicates, k = 10, a spectral similarity threshold of 0.7, an edge support threshold of 0.5, and a minimum spectral similarity threshold of 0.1 to allow the recovery of rescued connections. Further integration of CANOPUS predictions was done following the same approach as in Valencia-Revelo et al.^57^

### Fermentation, Extraction and Isolation

Cultivation and extraction was conducted as previously reported^16,20,43^. The scaled-up cultivation was done in 30 flasks of 500 mL containing solid rice BRFT medium, from which 2.9 g of crude extract were obtained after extraction.

The methanol extract was pre-fractionated using flash chromatography (Grace Reveleris^®^, Columbia, MD, USA) (silica cartridge 80 g, solvent A: DCM, solvent B: acetone, solvent C: ([DCM/acetone 8:2]: MeOH), gradient: 100% A for 5 min, increasing to 100% B in 20 min, followed by increasing to 100% solvent mixture C in 15 min and holding at 100% solvent C in 5 min). Ten fractions (F1–F10) were collected, even when fraction F1 was found to contain almost exclusively fatty acids and subsequently discharged.

Fraction F2 (383 × 2 mg) was re-purified by preparative reversed phase (RP)-HPLC (VP Nucleodur 100 − 10 C18 ec column (250 × 40 mm; Macherey-Nagel, Düren, Germany), solvent A: H_2_O + 0.1% formic acid, solvent B: ACN + 0.1% formic acid, flow rate 45 mL/min and UV detection at 210, 240, and 300 nm, gradient: From 50% to 75% B in 10 min, from 75% to 86% in 30 min, from 86% to 100% in 5 min, and 100% B isocratic for 5 min) to afford tortoisellide A (**1**) (1.5 mg, tR = 25–28 min) and isocochliodinol (**4**) (376 mg, *t*_R_ = 19–21 min).

Fraction F3 and F4 were combined (301 mg) and further fractionated via size exclusion chromatography on Sephadex^®^LH 20 material (Pharmacia Fine Chemicals, Inc., New York, NY, USA) (solvent: MeOH; 33 × 820 mm; flow rate 3.4 mL/min). Six fractions (F11-F16) were collected, from which fraction F11 (43 mg) was purified using RP-HPLC (Gemini, 10 μm column (250 × 21.2 mm; Phenomenex, Torrance, CA, USA), solvent A: H_2_O + 0.1% formic acid, solvent B: ACN + 0.1% formic acid, flow rate 45 mL/min and UV detection at 210, 240, and 300 nm, gradient: From 5% to 65% B in 15 min, from 65% to 100% in 35 min, and 100% B isocratic for 10 min) to afford cryptosphaerolide (**3**) (7.3 mg, *t*_R_ = 42–44 min).

Also, F6 and F7 were combined and purified using RP-HPLC (XBridge^®^ Prep C18, 5 μm OBDTM column (250 × 19 mm; Waters, Milford, MA, USA), solvent A: H_2_O + 0.1% formic acid, solvent B: ACN + 0.1% formic acid, flow rate 45 mL/min and UV detection at 210, 240, and 300 nm, gradient: From 5% to 65% B in 15 min, from 65% to 100% in 35 min, and 100% B isocratic for 10 min) to afford thio-grahamimycin A (**2**) (3.4 mg, *t*_R_ = 30–33 min).

### Spectral data

**Tortoisellide A (1)**: Blue to green oil; UV: 232, 322 nm; ^1^H NMR (700 MHz) and 13C NMR (175 MHz) data in DMSO-*d*_6_: see Table 1; ESI-MS: *m/z* 399.18 [M – H]^−^ and 401.30 [M + H]^+^; HR-ESI-MS: *m/z* 383.2941 [M – H_2_O + H]^+^, 401.3046 [M + H]^+^ and 423.2867 [M + Na]^+^ (calculated for C_26_H_40_O_3_, 400.2977). SMILES: CC(= O)/C(C) = C/C = C/C(C) = C/[C@H]1 C(O)O[C@H]([C@@H]1 C)/C(C) = C/C = C/C(C)CCCC.

**Thio-grahamimycin (2)**: Brown oil; [α]^20^_D_ −43° (c 0.001, MeOH); UV (MeOH) λ_max_ (log ε) 218 (2.2); ^1^H‐NMR and^13^C‐NMR see Table 2; ESI‐MS: *m/z* 402.95 [M – H]^−^and 405.10 [M + H]^+^; HR-ESI-MS: *m/z* 387.1108 [M – H_2_O + H]^+^, 405.1215 [M + H]^+^ and 427.1034 [M + Na]^+^ (calculated for C_17_H_24_O_9_S, 404.1141). SMILES: O=C1O[C@H](C)C/C = C/C(= O)O[C@H](C)C[C@@H](O)C(= O)CC1SC[C@H](O)C(O) = O.

**Cryptosphaerolide (3)**: Brown to yellow oil; ^1^H-NMR and^13^C‐NMR see Table S3; ESI‐MS: *m/z* 465.36 [M – H]^−^ and 467.34 [M + H]^+^; HR-ESI-MS: *m/z* 467.3000 [M + H]^+^ (calculated for C_26_H_42_O_7_, 466.2931). SMILES: C[C@@]12 C[C@H]3 C(= C)CO[C@@]3(O)[C@H]3O[C@]32[C@@H](CC[C@@H]1 C)OC(= O)[C@@](O)(CO)C[C@@H](C)C[C@@H](C)CC.

**Isocochliodinol (4)**: Dark purple powder; ^1^H-NMR (500 MHz, DMSO-*d*_6_): *δ*_H_ 11.22 (br s, NH–1’, NH–1’’), 10.65 (br s, 2–OH, 5–OH), 7.44 (d, *J* = 2.6 Hz, H–2’, H–2’’), 7.32 (d, *J* = 8.2 Hz, H–4’, H–4’’), 7.17 (br s, H–7’, H–7’’), 6.82 (dd, *J* = 8.2, 1.5 Hz, H–5’, H–5’’), 5.36 (m, H–11’, H–11’’), 3.39 (dd, *J* = 7.3, Hz, H–10’, H–10’’), 1.73 (m, H_3_–14’, H_3_–14’’), 1.72 (m, H_3_–13’, H_3_–13’’) ppm; ^13^C‐NMR (500 MHz, DMSO-*d*_6_): *δ*_C_ 136.1 (C, C–8’, C–8’’), 134.1 (C, C–6’, C–6’’), 131.0 (C, C–12’, C–12’’), 126.9 (CH, C–2’, C–2’’), 124.6 (C, C–9’, C–9’’), 124.3 (CH, C–11’, C–11’’), 121.5 (CH, C–4’, C–4’’), 119.7 (CH, C–5’, C–5’’), 111.0 (C, C–3, C–6), 110.3 (CH, C–7’, C–7’’), 104.4 (C, C–3’, C–3’’), 33.9 (CH_2_, C–10’, C–10’’), 25.6 (CH_3_, C–13’, C–13’’), 17.7 (CH_3_, C–14’, C–14’’); ESI‐MS: *m/z* 505.14 [M – H]^−^ and 507.32 [M + H]^+^; HR-ESI-MS: *m/z* 507.2438 [M + H]^+^ (calculated for C_32_H_30_N_2_O_4_, 506.2362). SMILES: C\C(C) = C/CC1CC2[NH]CC(C3C(O)C(O)C(C(O)C3O)C3C[NH]C4CC(CCC43)C\C = C(/C)C)C2CC1.

### Biological assays

The antimicrobial activity of each compound was evaluated by determining the minimum inhibitory concentration (MIC) against five fungi (i.e., *Candida albicans*, *Mucor hiemalis*, *Rhodotorula glutinis*, *Schizosaccharomyces pombe* and *Wickerhamomyces anomalus*) and against different Gram-positive (*Bacillus subtilis*,* Mycolicibacterium smegmatis* and *Staphylococcus aureus*) and Gram-negative (*Acinetobacter baumannii*,* Chromobacterium violaceum*,* Escherichia coli* and *Pseudomonas aeruginosa*) bacteria, using nystatin as a positive control against all the tested fungi and oxytetracycline against all the bacteria, except for *A. baumannii*,* My. smegmatis* and *Ps. aeruginosa*, against which ciprobay, kanamycin and gentamycin were used, respectively. The cytotoxicity of all compounds against seven mammalian cell lines obtained from the DSMZ (German Collection of Microorganisms and Cell Cultures, Braunschweig, Germany), i.e. human endocervical adenocarcinoma KB 3.1 (ACC 158), breast cancer MCF-7 (ACC 317), lung cancer A549 (ACC 107), ovary cancer SK-OV-3 (ACC HTB 77), prostate cancer PC-3 (ACC 465), squamous cancer A431 (ACC 91), and mouse fibroblasts L929 (ACC 2), was determined by the MTT method using epothilone B as the positive control. Both biological assays were performed following the protocols previously described^16^.

## Electronic Supplementary Material

Below is the link to the electronic supplementary material.

Supplementary Material 1

## Data Availability

All data related to structure elucidation and bioassays are available as Supplementary Material. The DNA sequences are deposited in GenBank (https://www.ncbi.nlm.nih.gov/genbank/). Molecular networking related datasets are available through Zenodo (https://zenodo.org/records/19733366).
