# Supplementary material for: Structurally diverse secondary metabolites from the dung-inhabiting fungus Botryotrichum murorum
Source: Sci Rep. 2026 May 14;16:15180. doi: 10.1038/s41598-026-52958-x (PMC13176333; doi:10.1038/s41598-026-52958-x)
Supplement: Supplementary file 1 — Supplementary Material 1 [file 41598_2026_52958_MOESM1_ESM.docx]

Supplementary Information

Structurally diverse secondary metabolites from the dung-inhabiting fungus *Botryotrichum murorum*

Esteban Charria-Girón^1,2^, Yong-Yue Liu^1^, Frank Surup^1,3*^, Yasmina Marin-Felix^1,3*^

^1^ Department Microbial Drugs, Helmholtz Centre for Infection Research (HZI), and German Centre for Infection Research (DZIF), Partner Site Hannover/Braunschweig, Inhoffenstrasse 7, 38124 Braunschweig, Germany

^2^ Bioinformatics Group, Wageninen University & Research, Droevendaalsesteeg 1, 6708 PB Wageningen, The Netherlands.

^3^ Institute of Microbiology, Technische Universität Braunschweig, Spielmannstraße 7, 38106 Braunschweig, Germany.

* Corresponding Author: f.surup@tu-braunschweig.de (Frank Surup); yasmina.marinfelix@helmholtz-hzi.de (Yasmina Marin-Felix)

**Table S1.** Dereplicated metabolites from the BRFT culture of *B. murorum*.

| **Compound** | ***m/z*** | **rt** | **Formula** | **Annotation*** | **Database** |
| --- | --- | --- | --- | --- | --- |
| Curvicollide A | 397.2737 [M-2H_2_O+H]^+^ | 13.10 | C_26_H_40_O_5_ | Level 2 | NP Atlas (Order: Sordariales) |
| Curvicollide A | 397.2737 [M-2H_2_O+H]^+^ | 15.30 | C_26_H_40_O_5_ | Level 2 | NP Atlas (Order: Sordariales) |
| Curvicollide A | 415.2838 [M-H_2_O+H]^+^ | 16.38 | C_26_H_40_O_5_ | Level 2 | NP Atlas (Order: Sordariales) |
| Curvicollide B | 415.2840 [M−H_2_O+H]^+^ | 16.09 | C_26_H_40_O_5_ | Level 2 | NP Atlas (Order: Sordariales) |
| Chaetorcinol | 369.2423 [M−H_2_O+H]^+^ | 15.85 | C_24_H_34_O_4_ | Level 2 | NP Atlas (Order: Sordariales) |
| Cochliodinol | 507.2272 [M+H]^+^ | 17.20 | C_32_H_30_N_2_O_4_ | Level 2 | NP Atlas (Order: Sordariales) |
| 27-epi-tryptoquivaline | 529.2090 [M−H_2_O+H]^+^ | 17.26 | C_29_H_30_N_4_O_7_ | Level 2 | NP Atlas (Order: Sordariales) |

*Level 2 to putative annotations by comparison of measured MS/MS spectra and *in silico* predicted MS/MS spectra of compounds reported for members of the Sordariales in NP Atlas. rt = retention time in min.

**3**

**Fig. S1** Diagnostic COSY (bold lines) and HMBC (arrows) utilized in the structure elucidation process of **1**–**4**.

**Table S2.** ^1^H NMR data (700 MHz, pyridine-*d*_5_) of diagnostic signals of the (*S*)-MTPA and (*R*)-MTPA derivatives of **2** in ppm as well as calculated *Δδ*^SR^ [shifts of (*S*)-MTPA minus (*R*)-MTPA] values.

| pos. | (*S*)-Mosher's acid derivative | (*R*)-Mosher's acid derivative | *Δδ*^SR^ |
| --- | --- | --- | --- |
| 15 | 1.26, m | 1.15, m | 0.11 |
| 14 | 5.19, m | 5.05, m | 0.14 |
| 13a | 2.39, m | 2.32, m | 0.07 |
| 13b | 2.18, m | 2.18, m | 0 |
| 12 | 6.00, m | 5.97, m | 0.03 |
| 1'a | 3.81, m | 3.95, m | -0.14 |
| 1'b | 3.34, br s | 3.49, m | -0.15 |
| 2' | 6.08, m | 5.72, m | +0.36 |

**Fig. S2** *Δδ*^SR^ values for MPTA esters of **2** diagnostic for 12*R*,2’*S* configuration.

**Table S3.** NMR data (^1^H 700 MHz, ^13^C 175 MHz) of cryptosphaerolide (**3**) in CHCl_3_-*d*.

| Atom# | *δ*_C_, mult. | *δ*_H_, mult. | COSY | ROESY | C to H HMBC |
| --- | --- | --- | --- | --- | --- |
| 1 | 76.8, CH | 4.65, br s | 2a, 2b | 9 | 3, 5, 9, 10, 16 |
| 2 | 28.3, CH_2_ | 1.93, m | 3b, 1 |  |  |
|  |  | 1.86, m | 1 |  | 4, 10, 1 |
| 3 | 25.6, CH_2_ | 1.65, m |  | 14 |  |
|  |  | 1.48, m | 2 |  |  |
| 4 | 38.4, CH | 1.63, m |  |  |  |
| 5 | 35.9, C |  |  |  |  |
| 6 | 36.4, CH_2_ | 1.58, dd (13.1,7.0) | 7 |  | 14, 5, 7, 10, 8 |
|  |  | 1.39, m |  |  | 14, 5, 7, 11 |
| 7 | 44.7, CH | 2.67, m | 6a |  | 6, 8, 11 |
| 8 | 102.5, C |  |  |  |  |
| 9 | 61.8, CH | 3.25, s |  | 1 | 7, 10, 1 |
| 10 | 63.0, C |  |  |  |  |
| 11 | 150.9, C |  |  |  |  |
| 12 | 70.1, CH_2_ | 4.45, br dd (12.7,1.8) | 12b, 13a, 13b |  | 7, 8, 13, 11 |
|  |  | 4.56, br dd (12.7,1.8) | 12a, 13a, 13b |  | 13, 11 |
| 13 | 104.4, CH_2_ | 4.94, br d (1.8) | 12a, 12b |  | 7, 12, 11 |
|  |  | 4.88, br d (1.8) | 12a, 12b |  | 7, 12, 11 |
| 14 | 15.2, CH_3_ | 1.20, s |  | 3a, 24b | 5, 6, 4, 10 |
| 15 | 15.2, CH_3_ | 0.92, d (6.3) |  |  | 3, 5, 4 |
| 16 | 174.9, C |  |  |  |  |
| 17 | 79.3, C |  |  |  |  |
| 18 | 41.2, CH_2_ | 1.68, dd (13.8,4.8) |  | 24a, 24b | 25, 19 |
|  |  | dd (13.8,7.4) |  | 24a, 24b | 25, 19, 20b, 24a, 17, 16 |
| 19 | 26.8, CH | 1.64, m |  |  |  |
| 20 | 45.7, CH_2_ | 0.95, m | 19, 20b, 21 |  | 26, 25, 22, 21 |
|  |  | 1.15, dt (13.4,6.7) | 19, 20a, 21 |  | 26, 25, 19, 22, 21, 18b |
| 21 | 31.5, CH | 1.39, m |  |  |  |
| 22 | 28.6, CH_2_ | 1.35, m | 23 |  |  |
|  |  | 1.03, br dd (13.2, 7.3) | 23 |  | 23, 26, 21 |
| 23 | 11.1, CH_3_ | 0.85, m | 22a, 22b |  | 22b, 21 |
| 24 | 69.0, CH_2_ | 3.58, d (11.1) | 24b | 25, 18a, 18b | 18b, 17, 16 |
|  |  | 3.79, d (11.1) | 24a | 14, 18a, 18b |  |
| 25 | 21.6, CH_3_ | 0.97, d (6.6) |  | 24a | 19, 18, 20 |
| 26 | 19.9, CH_3_ | 0.83, s |  |  | 22b, 21, 20 |

**Table S4.** ^2^*J*_CH_ and ^3^*J*_CH_ coupling constants utilized for *J*-conformational analysis for the C–17/C–18 bond.

| nuclei | type | Coupling constant [Hz] | interpretation |
| --- | --- | --- | --- |
| H18a-C16 | ^3^*J*_CH_ | 1.6 | small |
| H18b-C16 | ^3^*J*_CH_ | 7.8 | large |
| H18a-C17 | ^2^*J*_CH_ | 2.1 | small |
| H18b-C17 | ^2^*J*_CH_ | 5.6 | large |
| H18a-C24 | ^3^*J*_CH_ | 3.0 | small |
| H18b-C24 | ^3^*J*_CH_ | 2.2 | small |

**Table S5.** ^3^*J*_HH_ and ^3^*J*_CH_ coupling constants utilized for *J*-conformational analysis for the C18/C–19 bond.

| nuclei | type | Coupling constant [Hz] | interpretation |
| --- | --- | --- | --- |
| H18a-H19 | ^3^*J*_HH_ | 4.8 | small |
| H18b-H19 | ^3^*J*_HH_ | 7.4 | medium |
| H18a-C20 | ^3^*J*_CH_ | 4.0 | medium |
| H18b-C20 | ^3^*J*_CH_ | 2.9 | small |
| H18a-C25 | ^3^*J*_CH_ | 5.6 | large |
| H18b-C25 | ^3^*J*_CH_ | 4.8 | medium |

**Fig. S3** *J*-based analysis of six hypothetical rotamers to determine the stereochemistry of cryptosphaerolide (**3**) on C–17/C–18 bond.

**Fig. S4** *J*-based analysis of six hypothetical rotamers to determine the stereochemistry of cryptosphaerolide (**3**) on C–18/C–19 bond.

Geometry optimizations for the two potential diastereomers of cryptosphaerolide, namely with 17*S*,19*S*,21*S* and 17*R*,19*R*,21*R* configuration, were carried out using the built-in xTB method within ORCA 6.0.1, employing the GOAT procedure. Solvent effects were considered via both COSMO and ALPB models, with chloroform employed as the solvent in each case. In the COSMO model, the interatomic distance between the two relevant carbon atoms was found to be 4.086 Å for the 2*S*,4*S*,6*S* isomer and 4.582 Å for the 2*R*,4*R*,6*R* isomer. Under the ALPB model, the corresponding distances were 4.121 Å and 4.579 Å, respectively. These consistent results across both solvation models suggest a robust difference in spatial proximity between the two isomers.

Table S6 Energies and Distances of Cryptosphaerolide Isomers Calculated with ALPB and COSMO Models

| Method | GOAT with ALPB | | GOAT with COSMO | |
| --- | --- | --- | --- | --- |
| Conformation | 17*S*,19*S*,21*S* | 2*R*,4*R*,6*R* | 17*S*,19*S*,21*S* | 17*R*,19*R*,21*R* |
| Energy(kcal/mol) | -105.6699368 | -105.6687336 | -105.6797731 | -105.6789563 |
| Distance(Å) | 4.121 | 4.579 | 4.086 | 4.582 |


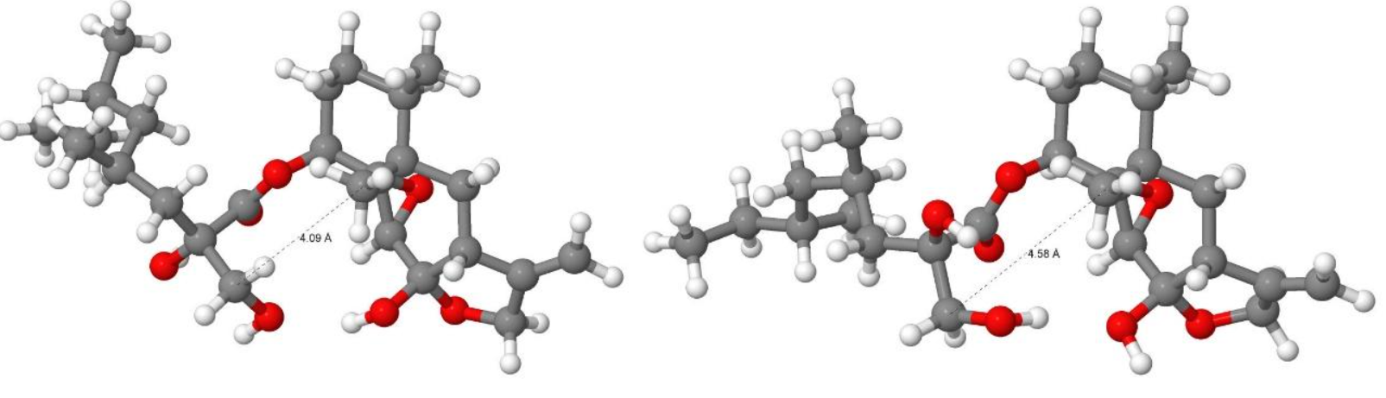


Figure S5. Optimized Geometries of Cryptosphaerolide Isomers 17*S*,19*S*,21*S* (left) and 17*R*,19*R*,21*R* (right) under COSMO Solvation Models


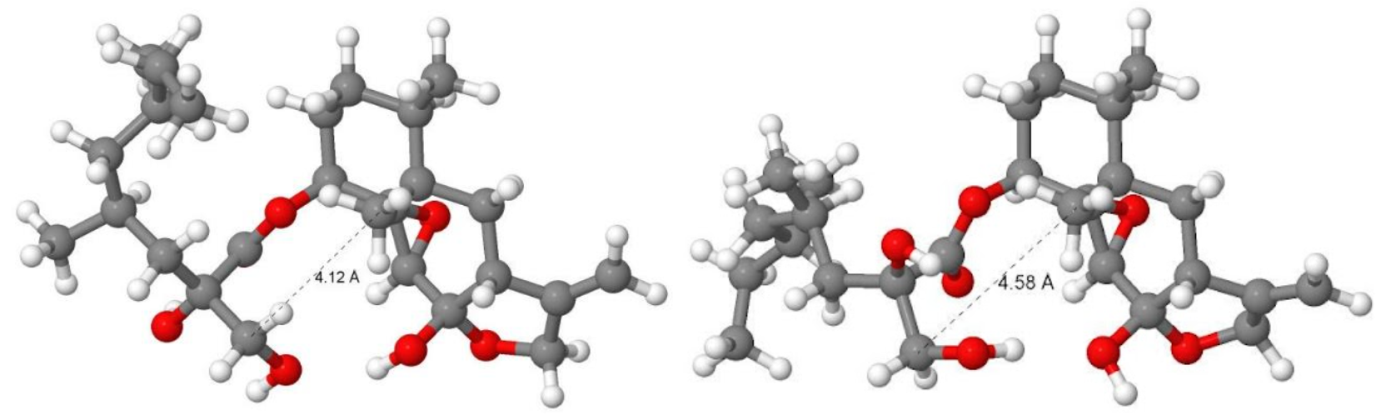


Figure S6. Optimized Geometries of Cryptosphaerolide Isomers 17*S*,19*S*,21*S* (left) and 17*R*,19*R*,21*R* (right) under ALPB Solvation Models

The computational results indicate that in the 17*R*,19*R*,21*R* configuration, the interaction between the two carbon atoms is sterically hindered by an intervening oxygen atom, effectively preventing spatial proximity. In contrast, the 17*S*,19*S*,21*S* configuration exhibits no such steric blockage, allowing closer spatial alignment between the carbon atoms. This finding is consistent with the ROESY NMR spectrum, which supports the hypothesis of spatial proximity between the two atoms in the *S* configuration. Therefore, by integrating the quantum chemical calculations with the ROESY NMR data and J-resolved analysis, we conclude that the absolute configuration of cryptosphaerolide is 17*S*,19*S*,21*S*.

**Fig. S7** ^1^H NMR spectrum (700 MHz, DMSO­–*d*_6_) of tortoisellide A (**1**)

**Fig. S8** ^13^C NMR spectrum (175 MHz, DMSO­–*d*_6_) of tortoisellide A (**1**)


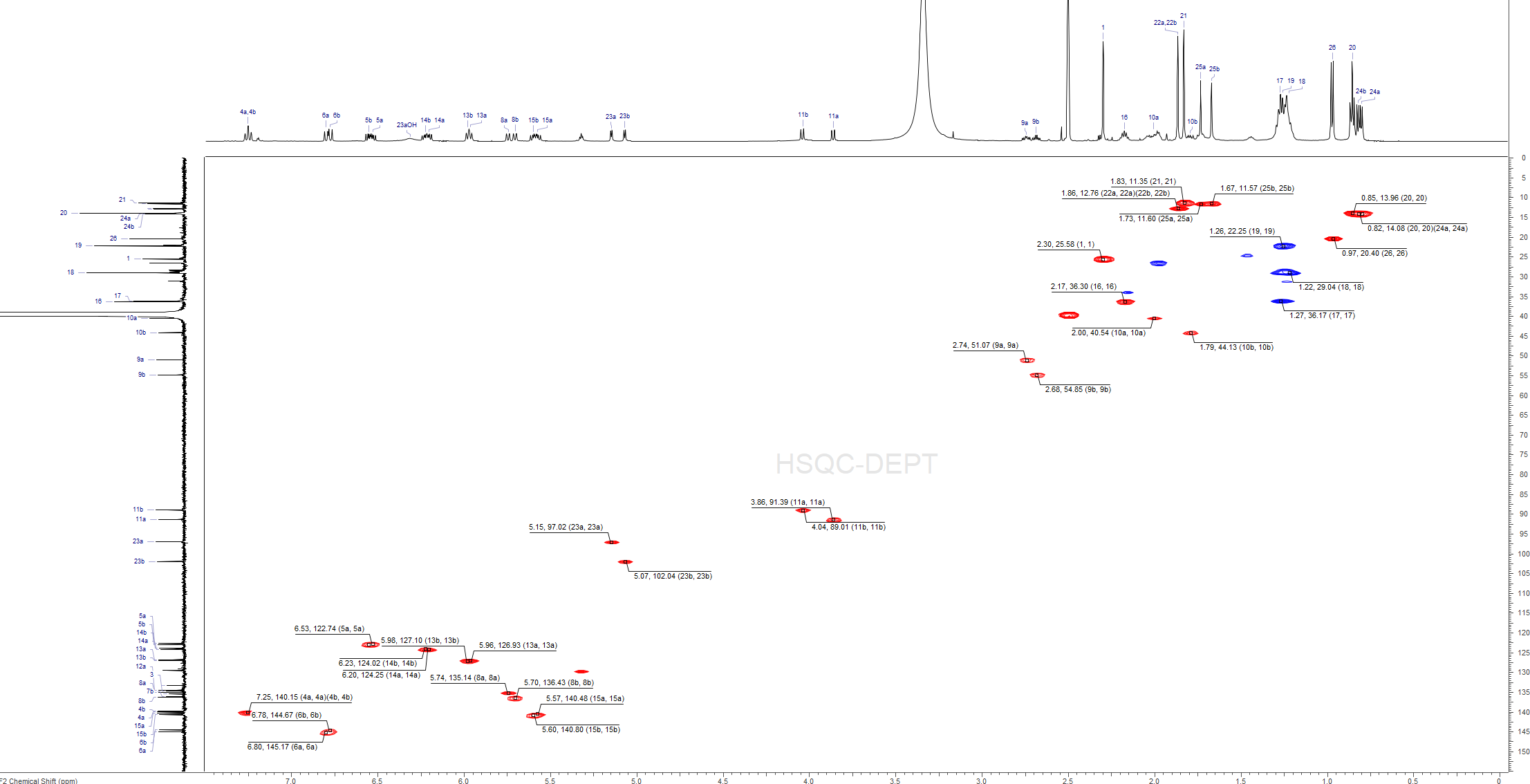


**Fig. S9** HSQC NMR spectrum (700 MHz, DMSO­–*d*_6_) of tortoisellide A (**1**)


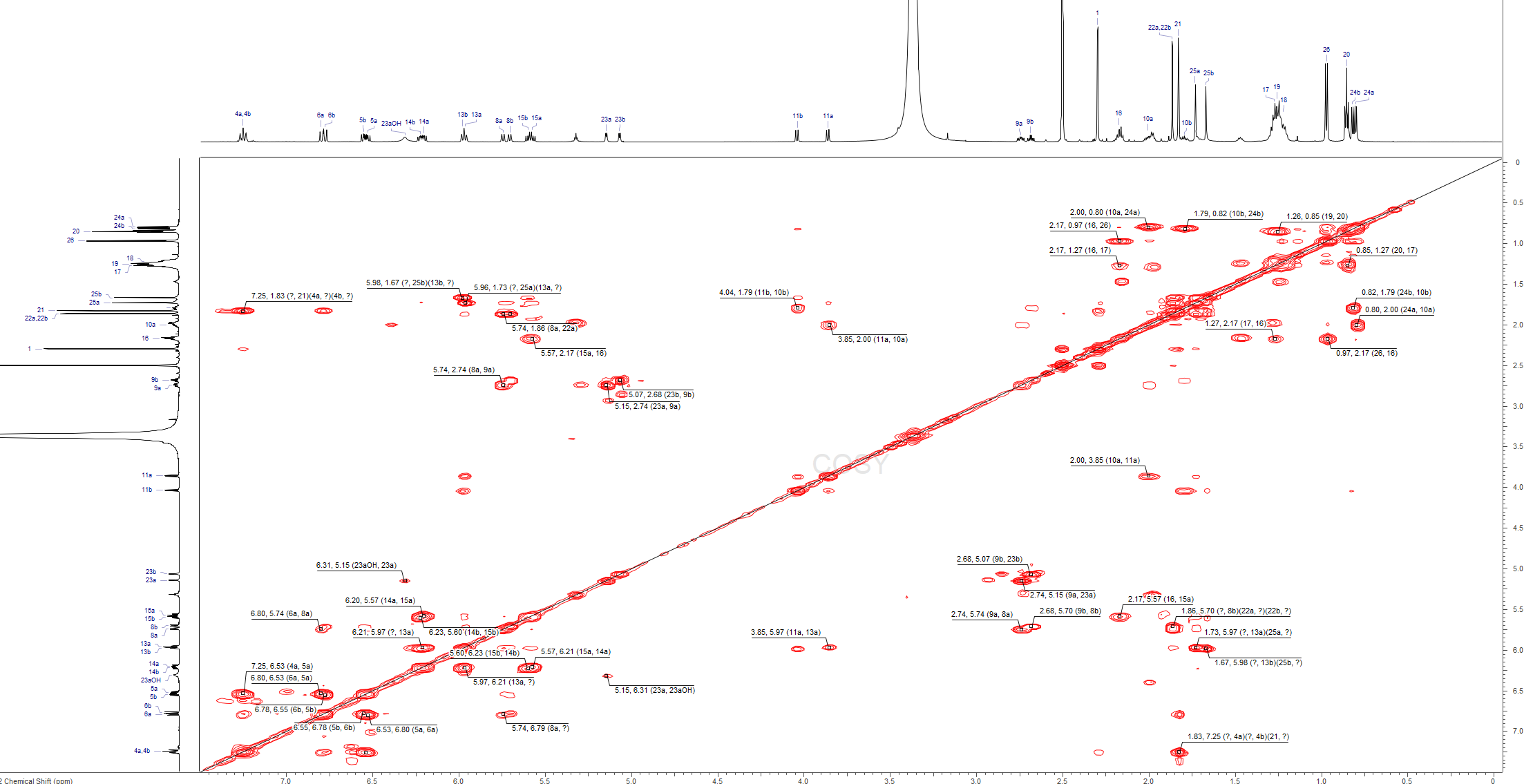


**Fig. S10** COSY NMR spectrum (700 MHz, DMSO­–*d*_6_) of tortoisellide A (**1**)


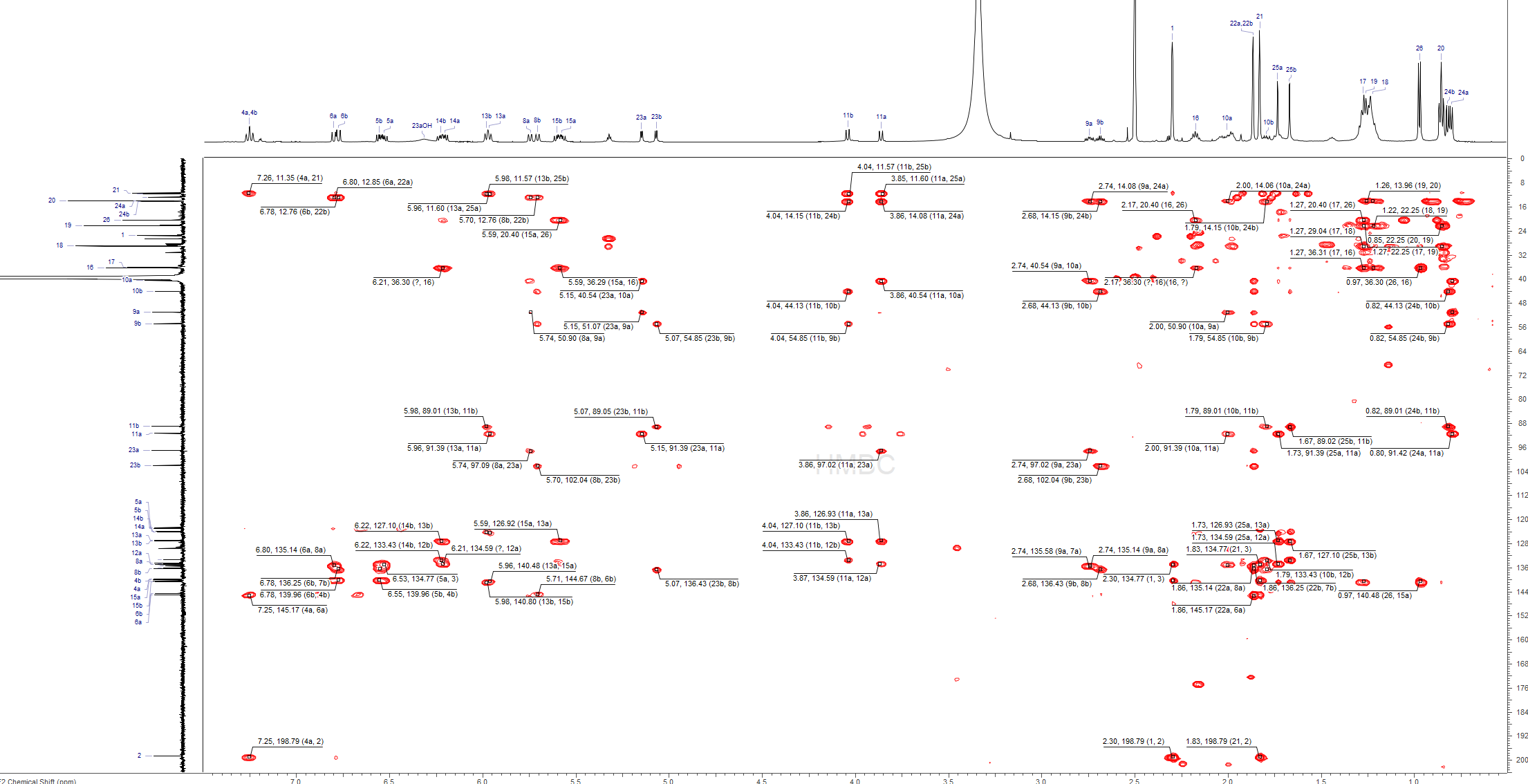


**Fig. S11** HMBC NMR spectrum (700 MHz, DMSO­–*d*_6_) of tortoisellide A (**1**)


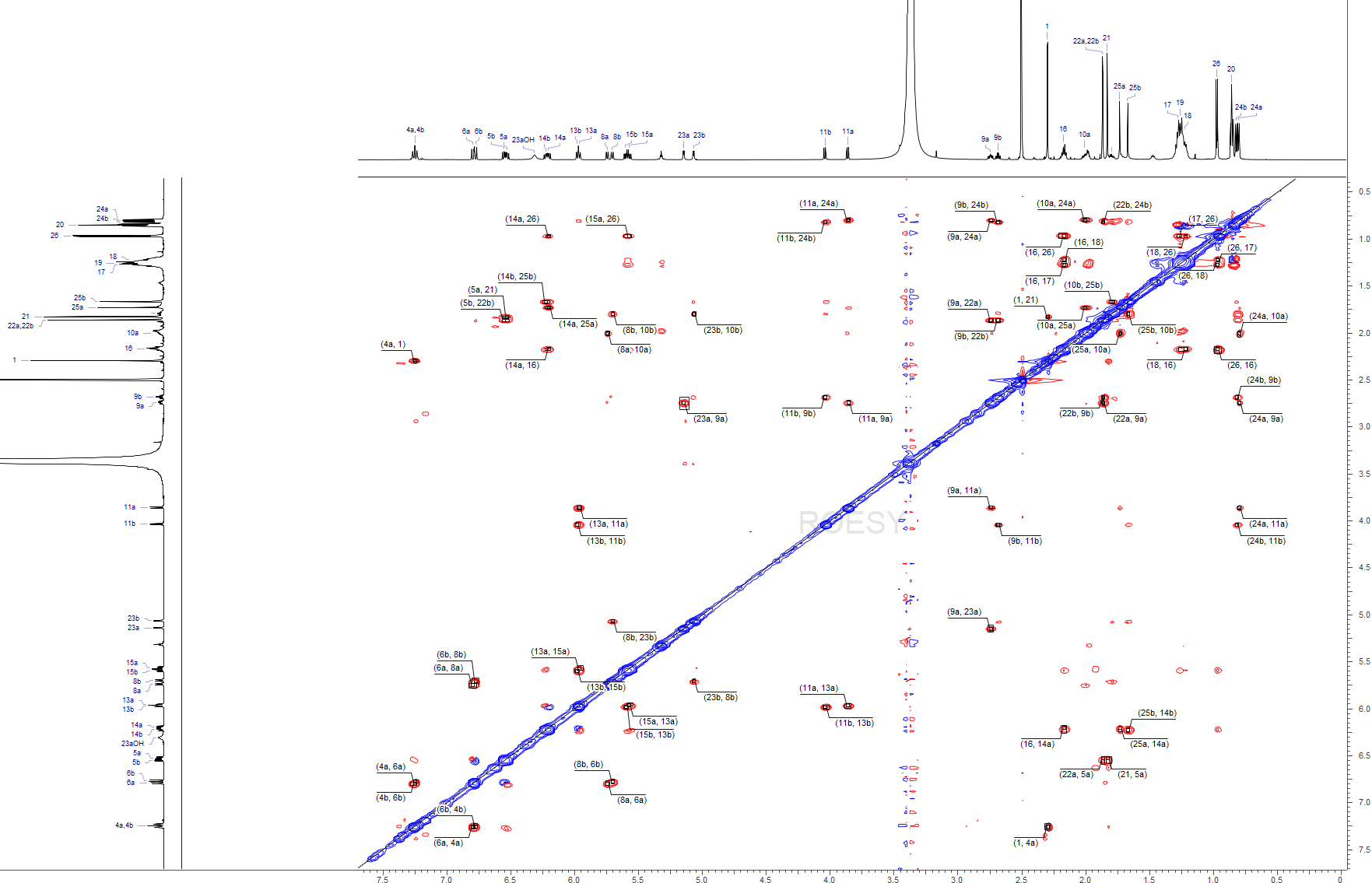


**Fig. S12** ROESY NMR spectrum (700 MHz, DMSO­–*d*_6_) of tortoisellide A (**1**)


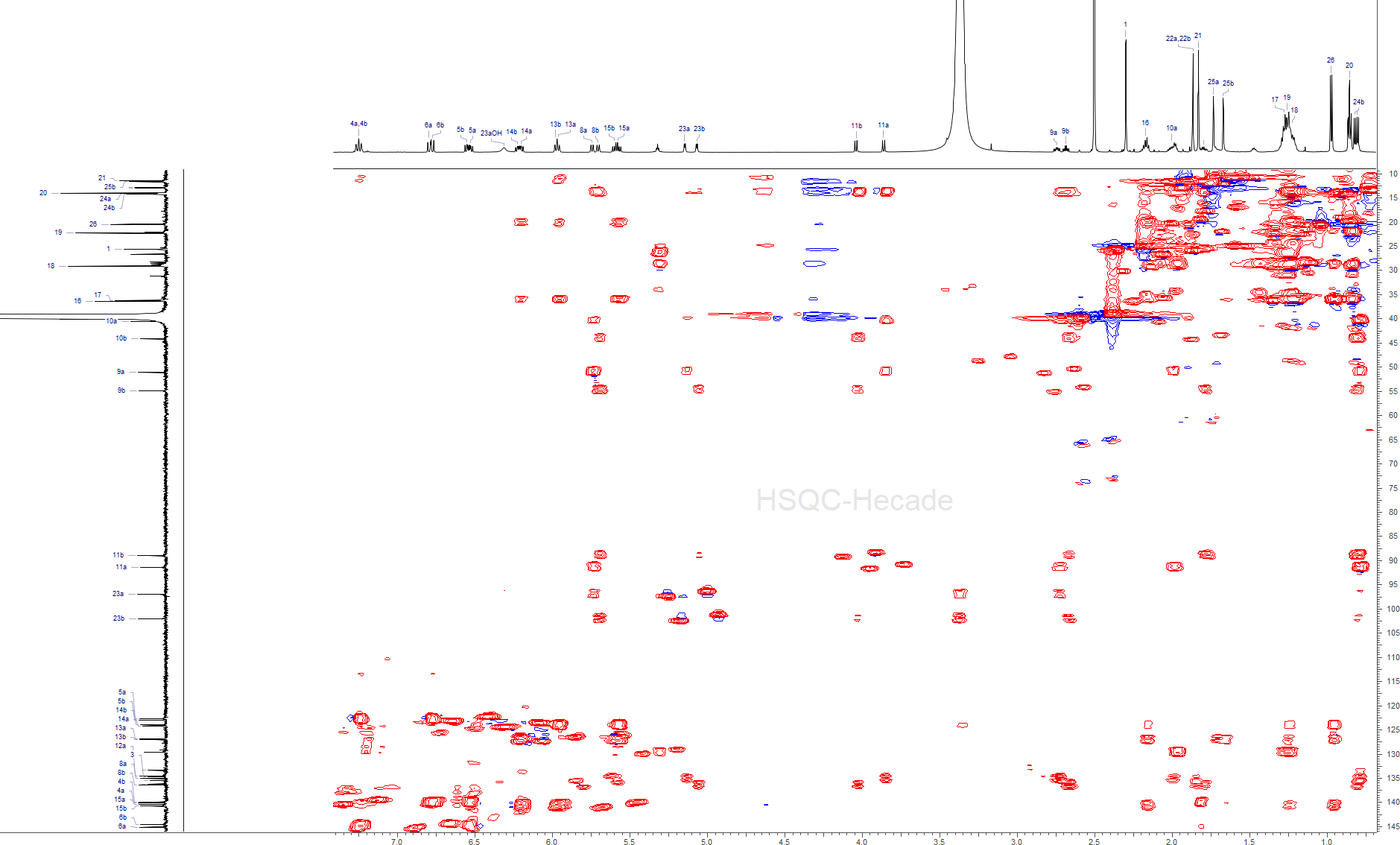


**Fig. S13** HSQC-Hecade NMR spectrum (700 MHz, DMSO­–*d*_6_) of tortoisellide A (**1**)


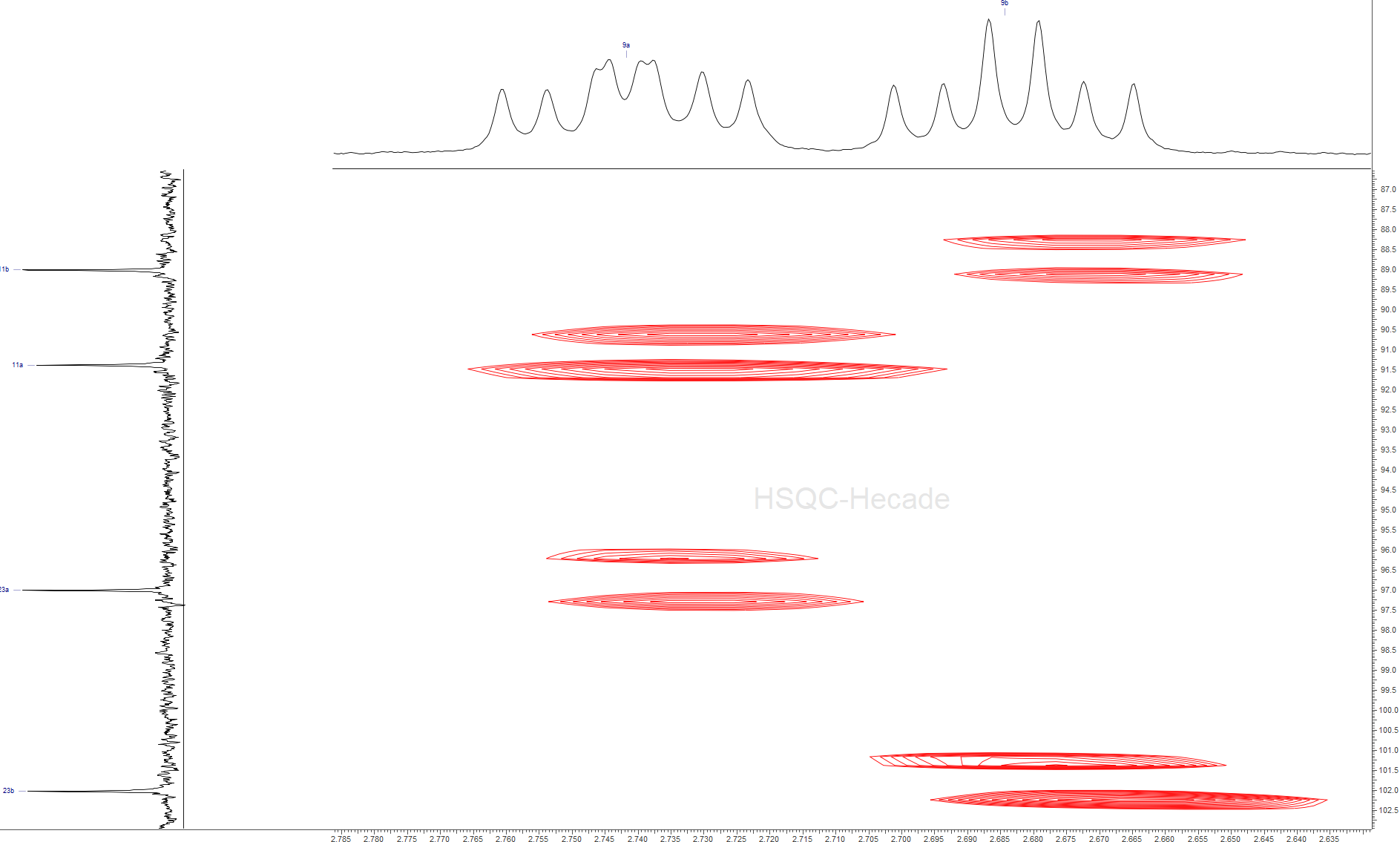


**Fig. S14** Section from the HSQC-Hecade NMR spectrum (700 MHz, DMSO­–*d*_6_) of tortoisellide A (**1**)

**Fig. S15** ^1^H NMR spectrum (700 MHz, DMSO­–*d*_6_) of **2**

**Fig. S16** ^13^C NMR spectrum (175 MHz, DMSO­–*d*_6_) of **2**


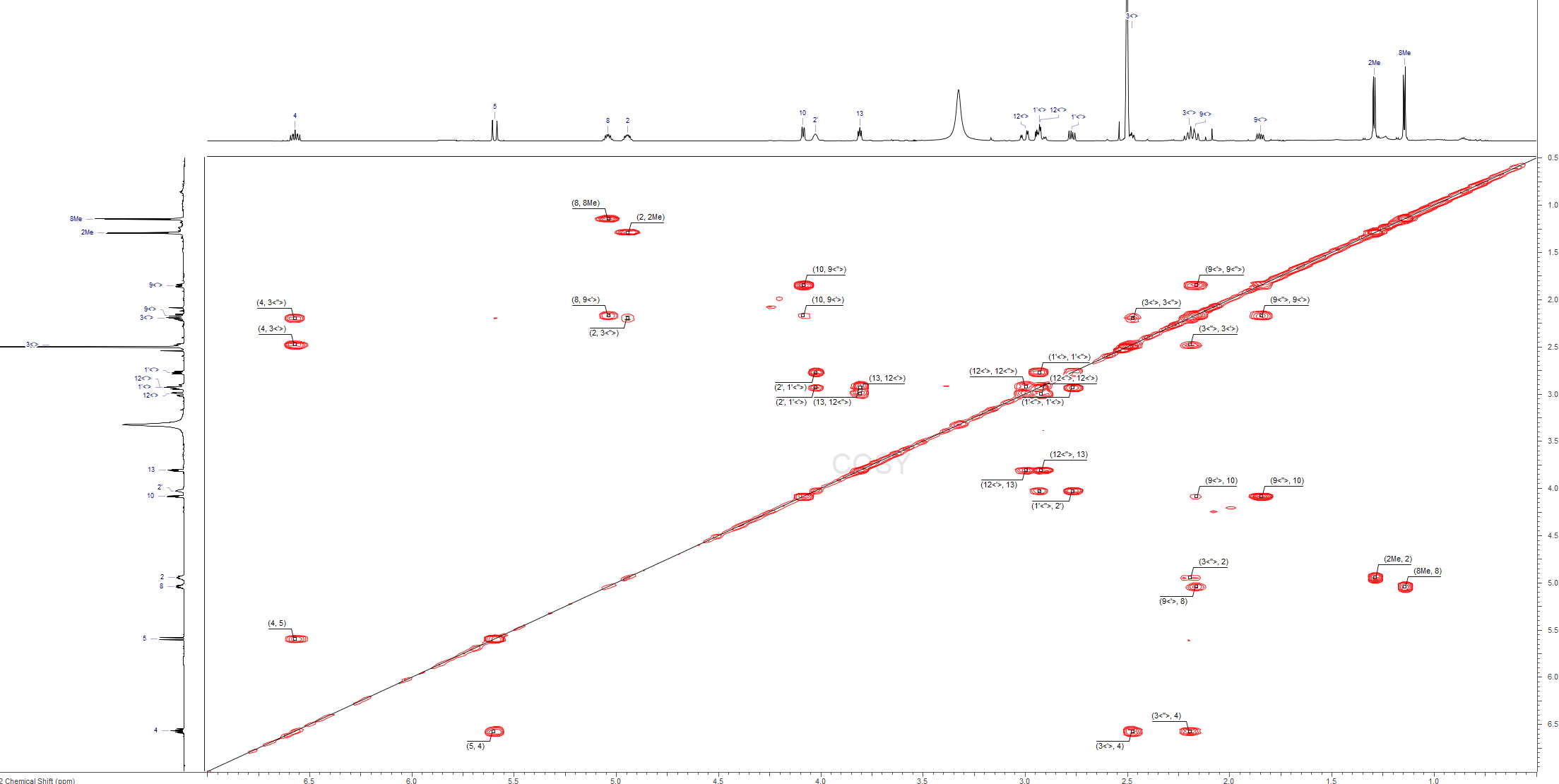


**Fig. S17** COSY NMR spectrum (700 MHz, DMSO­–*d*_6_) of **2**

**
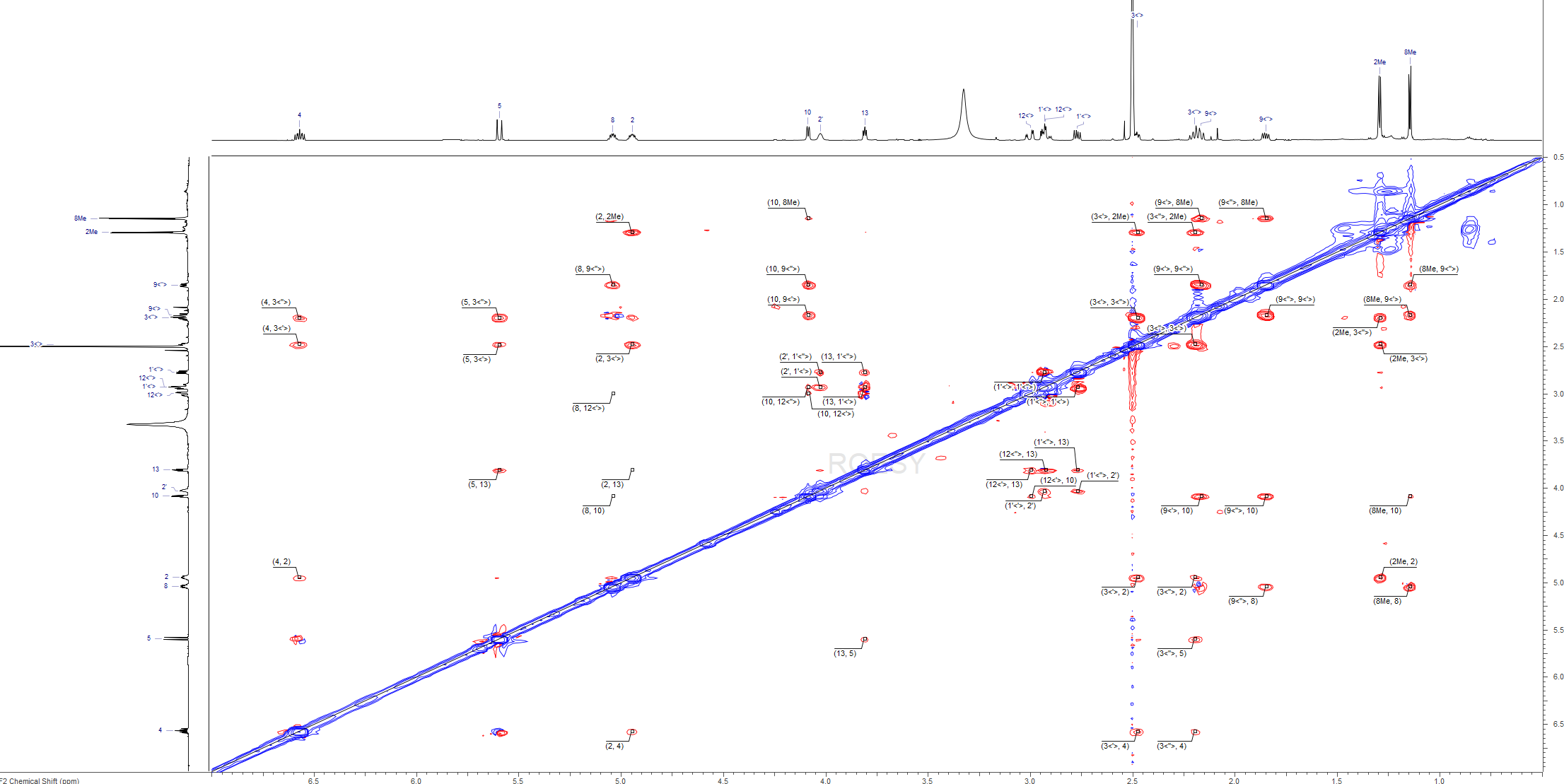
**

**Fig. S18** ROESY NMR spectrum (700 MHz, DMSO­–*d*_6_) of **2**


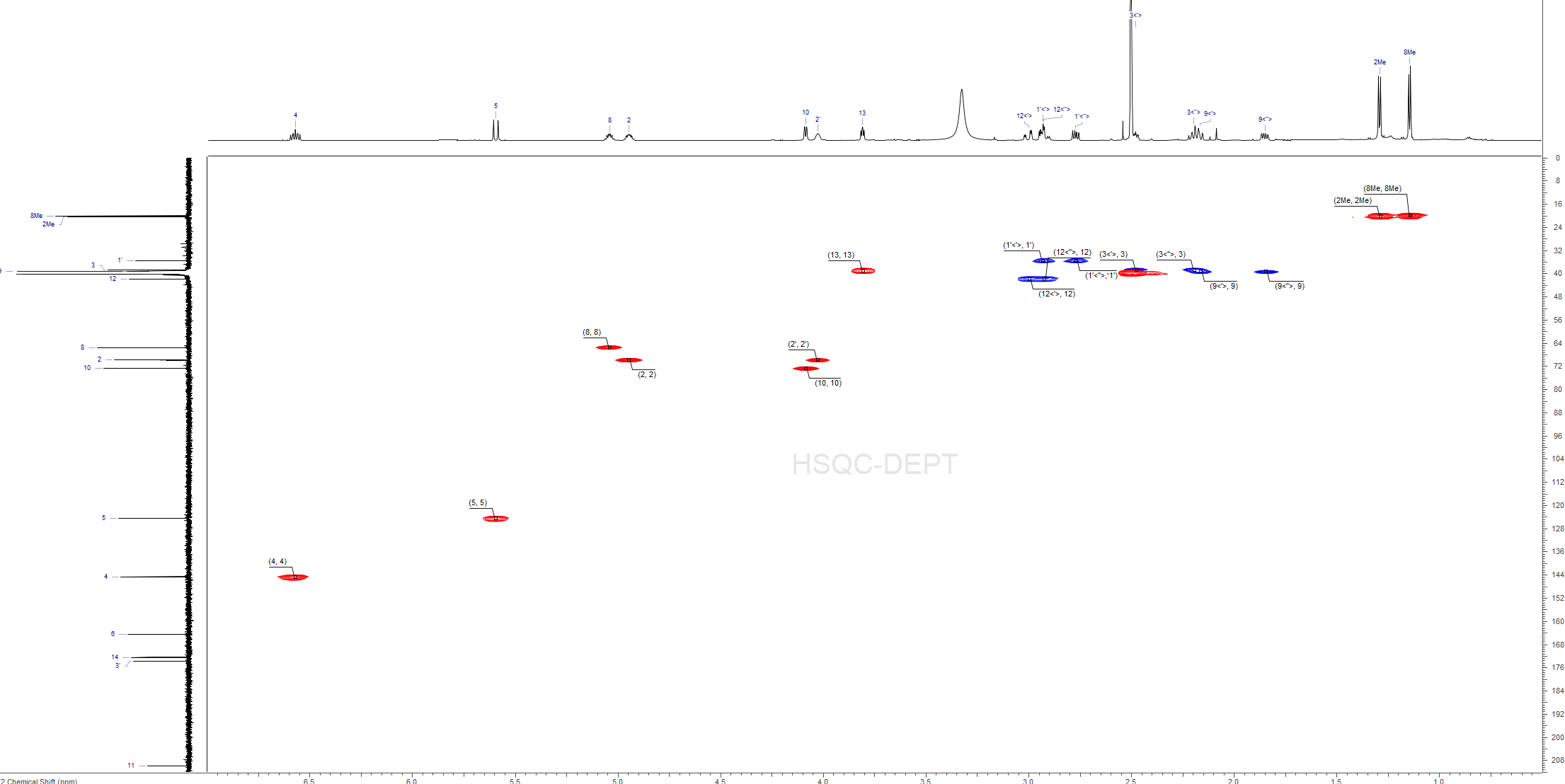


**Fig. S19** HSQC NMR spectrum (700 MHz, DMSO­–*d*_6_) of **2**


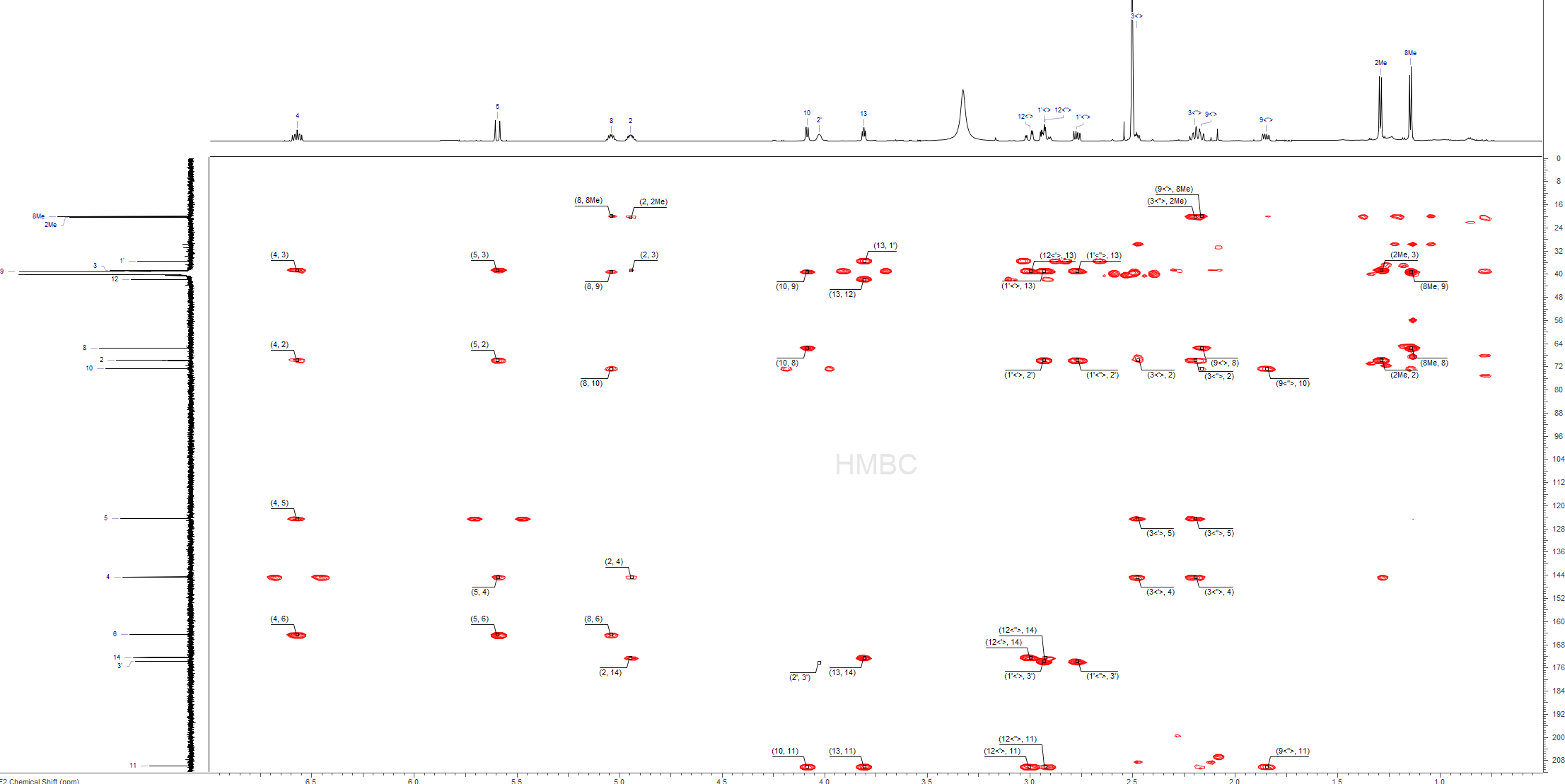


**Fig. S20** HMBC NMR spectrum (700 MHz, DMSO­–*d*_6_) of **2**

**Fig. S21** ^1^H NMR spectrum (500 MHz, CHCl_3_­–*d*) of **3**

**Fig. S22** ^13^C NMR spectrum (125 MHz, CHCl_3_­–*d*) of **3**


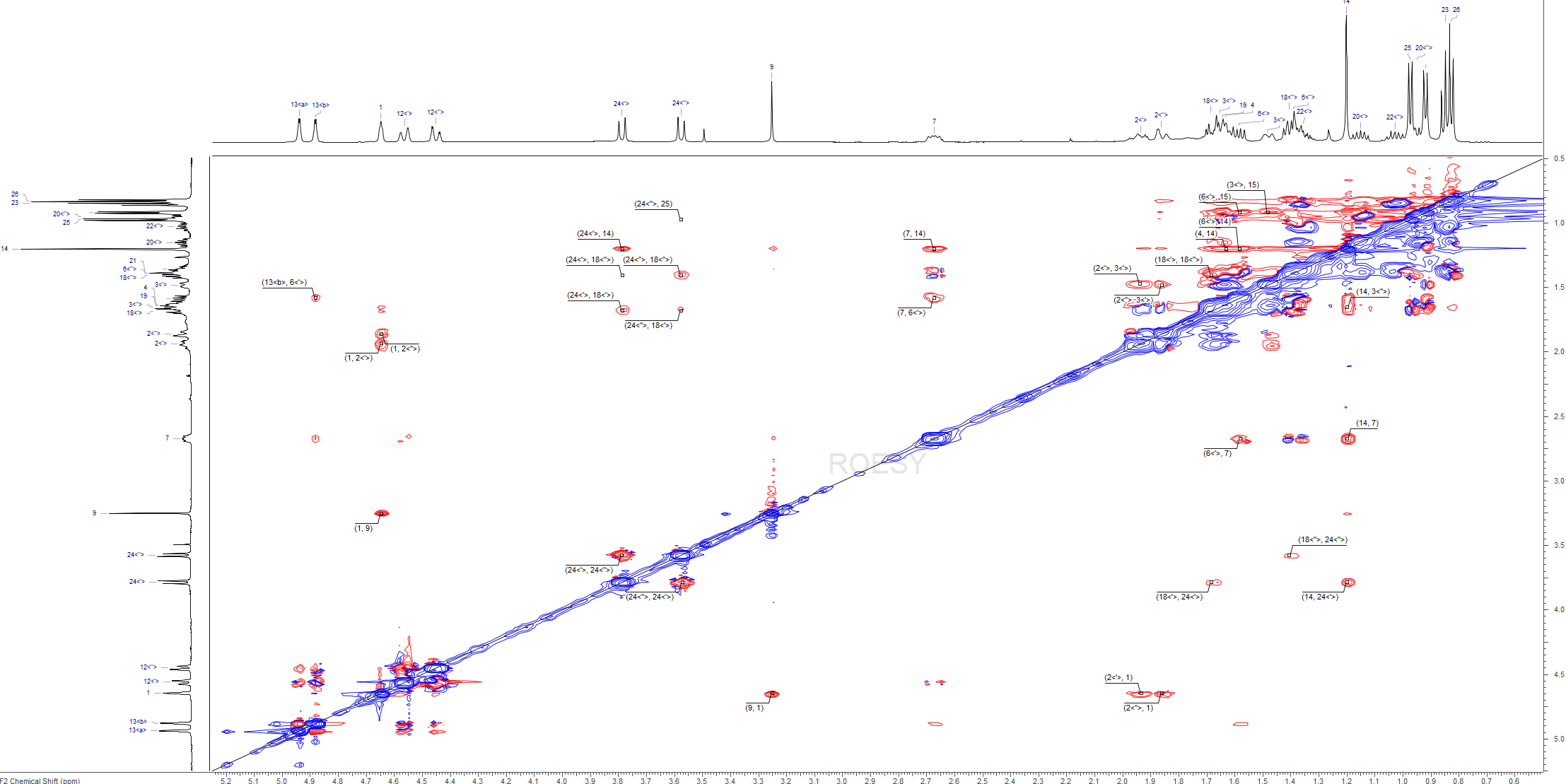


**Fig. S23** ROESY NMR spectrum (700 MHz, CHCl_3_­–*d*) of **3**

**
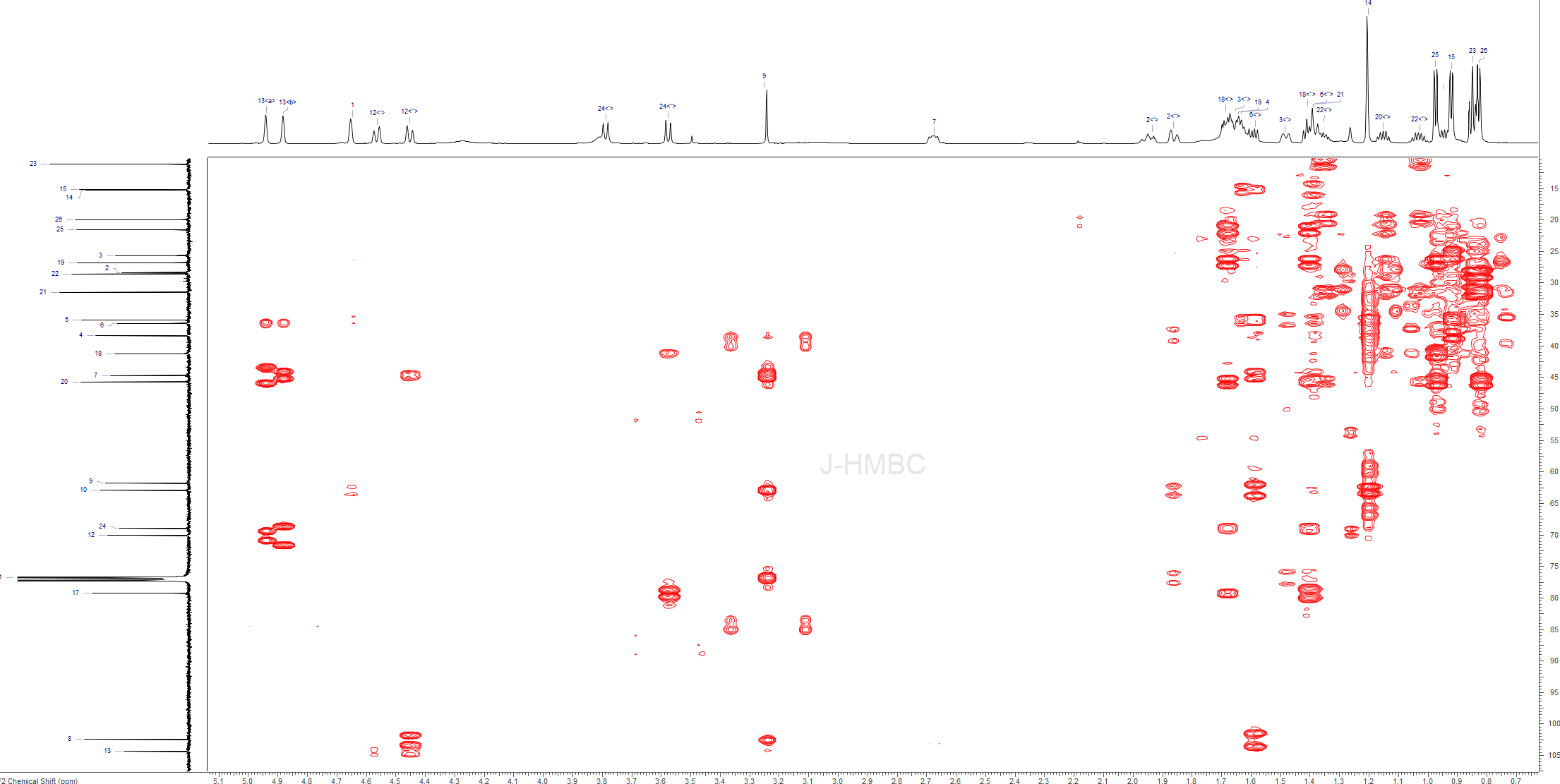
**

**Fig. S24** Section of the *J*-HMBC NMR spectrum (700 MHz, CHCl_3_­–*d*) of **3**

**
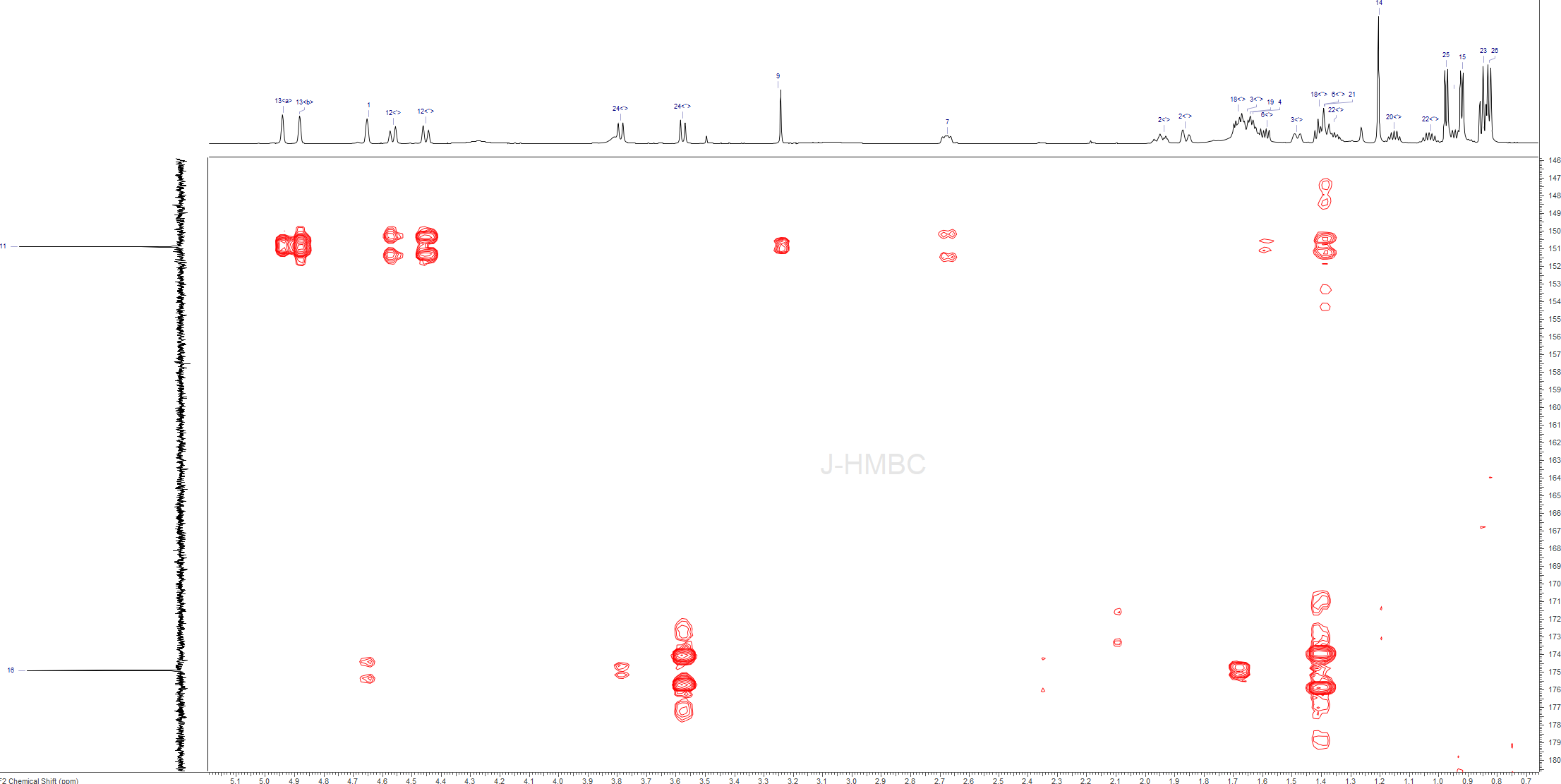
**

**Fig. S25** Section of the *J*-HMBC NMR spectrum (700 MHz, CHCl_3_­–*d*) of **3**

**Fig. S26** ^1^H NMR spectrum (500 MHz, DMSO­–*d*_6_) of **4**

**Fig. S27** ^13^C NMR spectrum (125 MHz, DMSO­–*d*_6_) of **4**

**
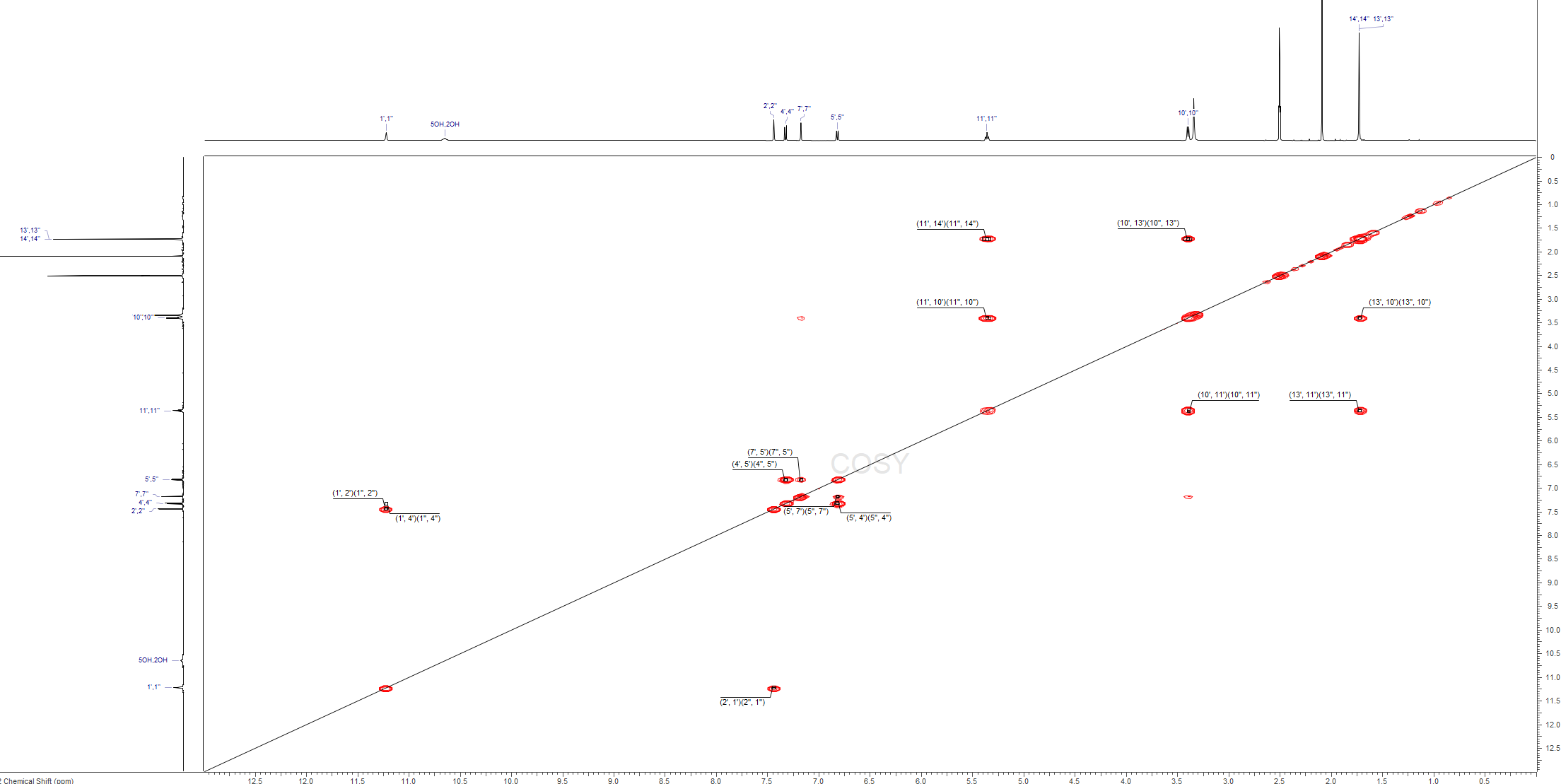
**

**Fig. S28** COSY NMR spectrum (500 MHz, DMSO­–*d*_6_) of **4**

**
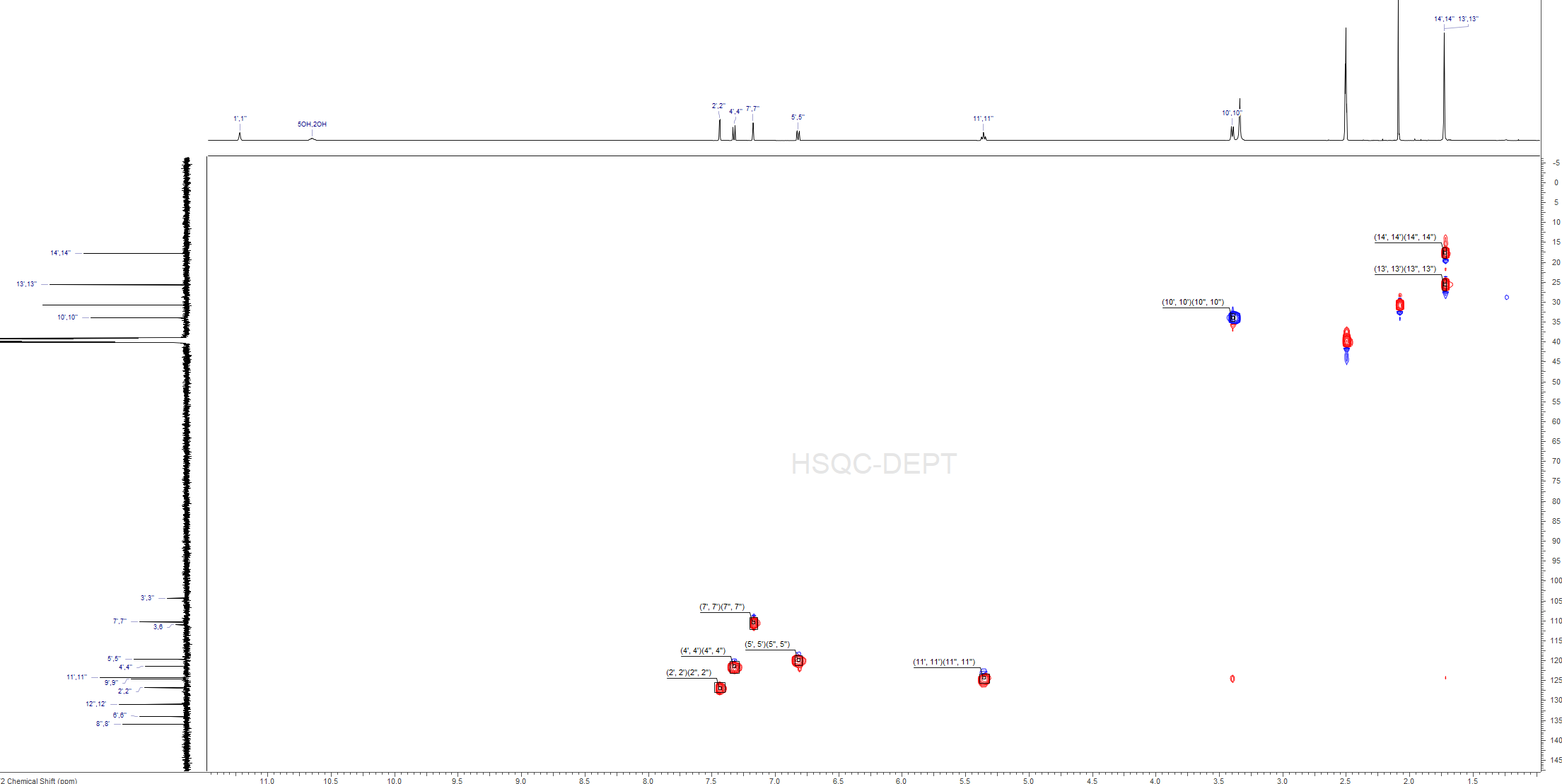
**

**Fig. S29** HSQC NMR spectrum (500 MHz, DMSO­–*d*_6_) of **4**


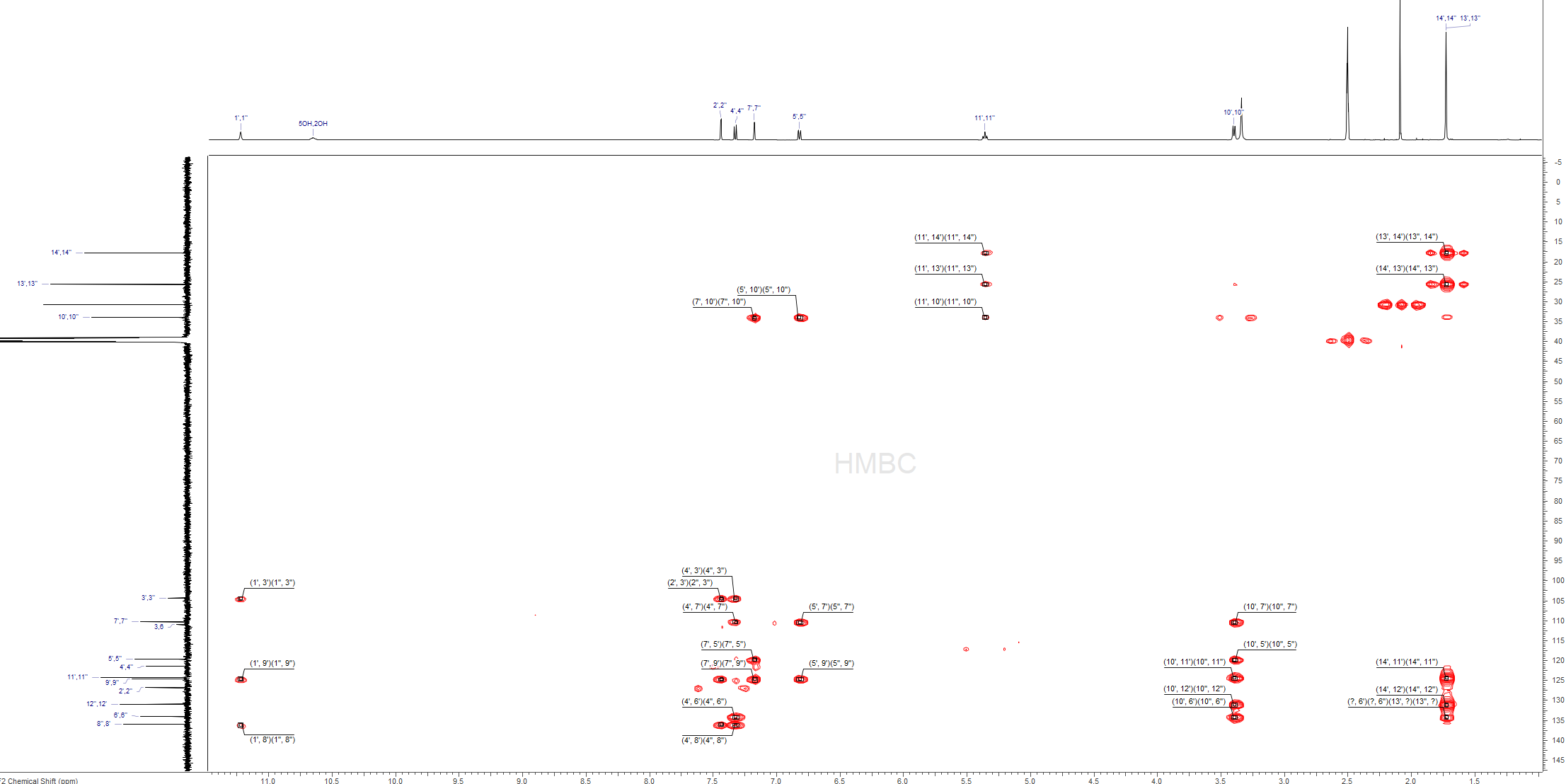


**Fig. S30** HMBC NMR spectrum (500 MHz, DMSO­–*d*_6_) of **4**

**Table S6**. Minimum inhibitory concentration (MIC, µg/mL) of isolated metabolites against bacterial and fungal test organisms. ^C^ ciprobay, ^G^ gentamicin, ^K^ kanamycin, ^O^ oxytetracycline, ^N^ nystatin, –: no inhibition observed under test conditions.

| **Test organism** | **1** | **2** | **3** | **Cochliodinol** | **Positive control** |
| --- | --- | --- | --- | --- | --- |
| *Candida albicans* | – | – | – | – | 8.3 ^N^ |
| *Schizosaccharomyces pombe* | – | – | – | – | 4.2 ^N^ |
| *Wickerhamomyces anomala* | – | – | – | – | 8.3 ^N^ |
| *Mucor hiemalis* | – | 16.6 | 66.6 | – | 4.2 ^N^ |
| *Mycolicibacterium smegmatis* | – | 66.6 | – | – | 1.70 ^K^ |
| *Bacillus subtilis* | – | 16.6 | 33.3 | 66.6 | 8.3 ^O^ |
| *Rhodotorula glutinis* | – | 33.3 | – | 66.6 | 2.1 ^N^ |
| *Staphylococcus aureus* | – | 8.3 | – | – | 0.21 ^O^ |
| *Acinetobacter baumannii* | – | – | – | – | 0.26 ^C^ |
| *Chromobacterium violaceum* | – | – | – | – | 0.42 ^O^ |
| *Escherichia coli* | – | – | – | – | 3.3 ^O^ |
| *Pseudomonas aeruginosa* | – | – | – | – | 0.42 ^G^ |

Alignment used in the phylogenetic study.

>Botryotrichum_atrogriseum_CBS_130_28

ATTACAGAGTTGCAAAACT-CCCTAAACCATTGTGAACGTTACCTTCAAACCGTTGCTTCGGCGGGCGGC-CCGGG-----TCC-GCCCGGTGCCCCCTGGCCCCCT----AGCGGGGCGCCCGCCGGAGGAAAACCCAACTCTTGAT-TATTATGGCCTCTCTGAGTCTTCTGTACTGAATAAGTCAAAACTTTCAACAACGGATCTCTTGGTTCTGGCATCGATGAAGAACGCAGCGAAATGCGATAAGTAATGTGAATTGCAGAATTCAGTGAATCATCGAATCTTTGAACGCACATTGCGCCCGCCAGTATTCTGGCGGGCATGCCTGTTCGAGCGTCATTTCAACCATCAAG-CCCCAGGCTTGTGTTGGGGACCTGCGGCTG--CCGCAGGCCCTGAAAACCAGTGGCGGGCTCGCTGT-CACACCGGGCGTAGTAGATTTTATCTCGCT----CTGGGCGTGCTGCGGGTTCCGGCCGTTAAAAAACCTTTTT------TACCCAA-GGTTGACCTCGGATCAGGTAGGAATACCCGCTGAACTTAAGCATATCAATAAG-CGGAGGAAAAGAAACCAACAGGGATTGCCCCAGTAACGGCGAGTGAAGCGGCAACAGCTCAAATTTGAAATCTGGCTTCGGCCCGAGTTGTAATTTGCAGAGGAAGCTTTAGGCGCGGCACCATCTGAGTCCCCTGGAACGGGGCGCCAAAGAGGGTGAGAGCCCCGTATAGATGGACGCCTAGCCTGTGTAAAGCTCCTTCGACGAGTCGAGTAGTTTGGGAATGCTGCTCAAAATGGGAGGTAAATTTCTTCTAAAGCTAAATATTGGCCAGAGACCGATAGCGCACAAGTAGAGTGATCGAAAGATGAAAAGCACTTTGAAAAGAGGGTTAAATAGCACGTGAAATTGTTGAAAGGGAAGCGCTTGTGACCAGACTTGCGCCGGGCGGATCATCCGGTGTTCTCACCGGTGCACTCCGCCCGGCTCAGGCCAGCATCGGTTCTCGTGGGGGGATAAAGGCACCGGGAACGTAGCTCCTCCGGGAGTGTTATAGCCCGGGGCGTAATGCCCTCGCGGGGACCGAGGACCGCGCTTCTGCAAGGATGCTGGTCACCCTGGTTCAAGGGTTGCGGAGGAAGAACGTCATCTCGTTCGAGGTTTCACTCGTCCGGGACATTCGCGACCGCGAGTTCAAGATTTTCTCCGACGCCGGCCGTGTCATGAGGCCGCTGTTCACCGTAGAACAAGAGAAGAAC---GGAGAGAGCGGCGCAGAGATGGGCCAGCTCATTCTCAACAAGGAGCACATTACGAGATTGGAGGCAGACAAGGAGTTGGGCAAGTATCACCCCGACTACTGGGGCTGGCAGGGCTTGTTGAAGTCGGGTGCTATCGAGTACCTCGATGCCGAAGAGGAGGAGACGGTCATGATCAGCATGACTCCCGAGGACCTCGACAAGTTCCGTTACCGCAAAATGGGGTTCATCGTCGAAGACAACTCTGGCCAAGGTAACAACAGGATCAAGACAAAGCCGAACCCGACCACGCACATGTACACCCACTGCGAGATCCACCCCAGCATGTTGCTCGGCATCTGCGCCAGCATCATTCCCTTCCCCGACCACAACCAGACGCGTCTGGGATGC---TTTTTT--GGACCCTGATCTACCCCAC--ACGCTGGGACGAC-GTCGTTCCACCACCAGTTCCCC--GACCAC----CCGACCGGC-----GCGAGAGCGGTAGC-GGCGCTGCCATGATGG-AATGTACACGATGCTGACTTTATCTTTCTACTTCAGGTCCACCTCCAGACCGGCCAATGCGTAAGTTGGATCGACTCGAACACGG------------CGACCGA----CCGGATGGAGCGGATGGACTGACAAGCAAC--CTCTCTAGGGTAACCAAATCGGTGCCGCTTTCTGGTATGTCCGGCCT-CAACATCAA-GCAATGACGTGTC-AAGACCGATCAAGACTGACTTCCCCTCCAGGCAGACCATTTCCGGCGAGCACGGCCTCGACGCCAATGGCGTGTATGTGACCGCCGCCGATTCCCGGCCGATGAAT--CCCCCGCTCACCGCTTCGATAGGTACAACGGCACCTCCGAGCTCCAGCTCGAGCGCATGAACGTCTACTTCAACGAGGTGAGTCGGGCCCGTAC-GCCT-----TTGGGCAGCCAGACGGTCGTGGTCGCTGACGA-CAGATATTCTGCAGGCGACCGGCAACAAGTATGTCCCTCGTGCCGTCCTTGTCGACTTGGAGCCCGGCACCATGGATGCCGTCCGCGCCGGTCCTTTCGGCCAGCTCTTCCGCCCCAACAACTT

>Botryotrichum_domesticum_UAMH_11929

ATTACAGAGTTGCAAAACT-CCCTAAACCATTGTGAACGTTACCTTCAAACCGTTGCTTCGGCGGGCGGC-CCGGG-----TCC-GCCCGGTGCCCCCTGGCCCCCT----AGCGGGGCGCCCGCCGGAGGAAAACCCAACTCTTGAT-TATTATGGCCTCTCTGAGTCTTCTGTACTGAATAAGTCAAAACTTTCAACAACGGATCTCTTGGTTCTGGCATCGATGAAGAACGCAGCGAAATGCGATAAGTAATGTGAATTGCAGAATTCAGTGAATCATCGAATCTTTGAACGCACATTGCGCCCGCCAGTATTCTGGCGGGCATGCCTGTTCGAGCGTCATTTCAACCATCAAG-CCCCGCGCTTGTGTTGGGGACCTGCGGCTG--CCGCAGGCCCTGAAAACCAGTGGCGGGCTCGCTGT-CACACCGGGCGTAGTAGATTTTATCTCGCT----CAGGGCGTGCTGCGGGTTCCGGCCGTTAAAAAGCCTTTTT------TACCCAA-GGTTGACCTCGGATCAGGTAGGAATACCCGCTGAACTTAAGCATATCAATAAG-CGGAGGAAAAGAAACCAACAGGGATTGCCTCAGTAACGGCGAGTGAAGCGGCAACAGCTCAAATTTGAAATCTGGCTTCGGCCCGAGTTGTAATTTGCAGAGGAAGCTTTAGGCGCGGCACCATCTGAGTCCCCTGGAACGGGGCGCCACAGAGGGTGAGAGCCCCGTATAGATGGACGCCTAGCCTGTGTAAAGCTCCTTCGACGAGTCGAGTAGTTTGGGAATGCTGCTCTAAATGGGAGGTAAATTTCTTCTAAAGCTAAATACCGGCCAGAGACCGATAGCGCACAAGTAGAGTGATCGAAAGATGAAAAGCACTTTGAAAAGAGGGTTAAATAGCACGTGAAATTGTTGAAAGGGAAGCGCTTGTGACCAGACTTGCGCCGGGCGGATCATCCGGTGTTCTCACCGGTGCACTCCGCCCGGCTCAGGCCAGCATCGGTTCTCGTGGGGGGATAAAGGCACCGGGAACGTAGCTCCTCCGGGAGTGTTATAGCCCGGGGCGTAATGCCCTCGCGGGGACCGAGGACCGCGCATCTGCAAGGATGCTGGTCACCCTGGTTCAGGGTTTGCGGAGGAAGAACGTCATCTCGTTCGAGGTTTCGCTCGTCCGGGACATTCGCGACCGCGAGTTCAAGATTTTCTCCGACGCGGGCCGTGTCATGAGGCCGTTGTTCACGGTAGAACAAGAGAAGAAC---GGAGAGAGCGGCGCAGAAATGGGCCAGCTTATTCTCAACAAAGAGCACATTACGAGATTGGAGGCGGACAAGGAGTTGGGCAAGTATCACCCCGACTACTGGGGCTGGCAGGGCTTGTTGAAGTCGGGCGCTATCGAGTACCTCGATGCCGAAGAAGAGGAGACGGTCATGATCAGCATGACTCCCGAGGACCTCGACAAGTTCCGTTACCGCAAGATGGGGTTCATCGTCGAAGACAACTCTGGCCAAGGTAACAACAGGATCAAGACAAAGCCGAACCCGGCCACGCACATGTACACCCACTGCGAGATCCACCCCAGCATGTTGCTCGGCATCTGCGCCAGCATCATTCCATTCCCCGACCACAACCAGACGCGTCTGGGATGC---TTTTTT-GGGATCCCGATCTACCCCAC--ACACTGGGACGAC-GTCGTTCCACCA---GTTC------ACCAC----CCGACCGAC-----GCGAGAGCGGTAGC-GGCGCTGCCATGATGG-AATGTACACGATGCTGACTTTATATTTCTCCTACAGGTCCACCTCCAGACCGGCCAATGCGTAAGTTGGATCGACTCGACCACGG------------CGACCGA----CCGGATGGAGCGGATGGACTGACAAGCAAC--CTCTCTAGGGTAACCAAATCGGTGCCGCTTTCTGGTATGTCCGGCCT-CAACATCAA-GCAATGACGTGTC-AAGACCGATCAAGACTGACTTCCCCTCTAGGCAGACCATTTCCGGCGAGCACGGCCTCGACGCCAATGGCGTGTATGTGACCGCCGCCGATTCCCGGCCGATGAAT--CCCCCGCTCACCGCTTCGATAGGTACAACGGCACCTCCGAGCTCCAGCTCGAGCGCATGAACGTCTACTTCAACGAGGTGAGTCGGGCCCCTACGGCCT-----TGGGGCAGCCAGACGGTCGTGGTCGCTGACGA-CAGATATTCTGCAGGCGGCAGGCAACAAGTATGTCCCTCGTGCCGTCCTTGTCGACTTGGAGCCCGGCACCATGG?????????????????????????????????????????????????

>Botryotrichum_geniculatum_CBS_144475

ATTACAGAGTTGCAAAACT-CCCTAAACCATTGTGAACGTTACCTACAAACCGTTGCTTCGGCGGGCGGCACCGGG-----CCC-GCCCGGTGCCCCTCGGCCCCCCC---AGCGGGGCGCCCGCCGGAGG-AAACCCAACTCTTGAT--ATCATGGCCTCTCTGAGTCTTCTGTACTGAATAAGTCAAAACTTTCAACAACGGATCTCTTGGTTCTGGCATCGATGAAGAACGCAGCGAAATGCGATAAGTAATGTGAATTGCAGAATTCAGTGAATCATCGAATCTTTGAACGCACATTGCGCCCGCCAGTATTCTGGCGGGCATGCCTGTTCGAGCGTCATTTCAACCATCAAG-CCCAGGGCTTGTGTTGGGGACCTGCGGCTG--CCGCAGGCCCTGAAAACCAGTGGCGGGCTCGCTGT-CACACCGGGCGTAGTAGATTTTATCTCGCT----CAGGGCGTGCTGCGGGTTCCGGCCGTTAAAAAGCCTTTTT------TACCCAA-GGTTGACCTCGGATCAGGTAGGAATACCCGCTGAACTTAAGCATATCAATAAG-CGGAGGAAAAGAAACCAACAGGGATTGCCCCAGTAACGGCGAGTGAAGCGGCAACAGCTCAAATTTGAAATCTGGCTTCGGCCCGAGTTGTAATTTGCAGAGGAAGCTTTAGGCGCGGCACCATCTGAGTCCCCTGGAACGGGGCGCCATAGAGGGTGAGAGCCCCGTAGAGATGGACGCCTAGCCTGTGTAAAGCTCCTTCGACGAGTCGAGTAGTTTGGGAATGCTGCTCAAAATGGGAGGTAAATTTCTTCTAAAGCTAAATACCGGCCAGAGACCGATAGCGCACAAGTAGAGTGATCGAAAGATGAAAAGCACTTTGAAAAGAGGGTTAAATAGCACGTGAAATTGTTGAAAGGGAAGCGCTTGTGACCAGACTTGCGCCGGGCAGATCATCCGGTGTTCTCACCGGTGCACTCTGCCCGGCTCAGGCCAGCATCGGTTCTCGCGGGGGGATAAAGGCACCGGGAACGTAGCTCCTCCGGGAGTGTTATAGCCCGGGGCGTAATGCCCTCGCGGGGACCGAGGACCGCGCTTCGGCAAGGATGCTGGTCACCTTGGTTCAAGGGCTTCGGAGAAGGAACGTCATTTCGTTCGAGGTTTCACTCGTCCGCGACATCCGCGACCGCGAGTTCAAGATCTTCTCCGATGCGGGCCGTGTCATGAGGCCGCTGTTTACCGTGGAGCAAGAGAAGAAC---GGAGAGAGCGGCGCCGAGATGGGACAGCTGATCCTCAACAAGGAGCACATTACGAGGTTGGAGGCCGACAAGGAGTTGGGCAAGTATCACCCCGACTACTGGGGCTGGCAGGGCTTGTTGAAGTCGGGTGCCATCGAGTACCTCGACGCCGAAGAGGAGGAGACGGTCATGATCAGCATGACTCCCGAGGACCTCGACAAGTTCCGTTACCGCAAGATGGGCTTCATCGTGGAAGACAACTCTGGCCAAGGTAACAACAGGATCAAGACAAAGCCGAACCCGGCCACGCACATGTACACCCACTGCGAGATCCACCCCAGCATGTTGCTCGGCATCTGCGCCAGCATCATCCCATTCCCCGACCACAACCAGACGCGTCCGGGAAGCT--TTTTTT-GGGCCCCTGATCTACCCCAC--ACATTGGGACGAC-GTCGTTCCACC----GCTACCC--GACCACCCGACCGACCGAC-----GCGAGGGCGATAGC-GGCGCTGCCATGATGGGAAGAAACACGAAGCTGACTCTGT-ATTCCACGACAGGTTCACCTCCAGACCGGCCAATGCGTAAGTTGGATCGACTCGAACACGG------------CGACCGA----CCGGATGGTGCGGGTGGACTGAC-AGCAAG--CTCTCCAGGGTAACCAAATCGGTGCCGCTTTCTGGTACGTTCGGCCA-CAACACCAA-GCACTGACGTGTC-AAGACCGATCAAGACTGACTTCCCTTGCAGGCAGACCATCGCCGGCGAGCACGGCCTCGACGCCAATGGCGTGTACGTGACCGCCGCCAGTTCCCGGCCGAAGACT---TCCCGCTCACCGCTTCGATAGGTACAATGGCACCTCCGAGCTCCAGCTCGAGCGCATGAACGTCTACTTCAACGAGGTTAGTCGGGCTCATACAGCTC---CAGCAAGCAACCAAACAACCATGTCCACTGACAA-CAGATGTTCTGCAGGCGTCCGGCAACAAGTATGTTCCTCGCGCCGTTCTGGTCGATCTGGAGCCCGGCACCATGGATGCCGTCCGCGCCGGTCCTTTCGGCCAGCTCTTCCGCCCCGACAACTT

>Botryotrichum_inquinatum_CBS_155_80

ATTACAGAGTTGCAAAACT-CCCTAAACCATTGTGAACGTTACCTTCAAACCGTTGCTTCGGCGGGCGGCACCGGG-----CCC-GCCCGGTGCCCCCTGGCCCCCCC---AGCGGGGCGCCCGCCGGAGG-AAACCCAACTCTTGATACATTATGGCCTCTCTGAGTCTTCTGTACTGAATAAGTCAAAACTTTCAACAACGGATCTCTTGGTTCTGGCATCGATGAAGAACGCAGCGAAATGCGATAAGTAATGTGAATTGCAGAATTCAGTGAATCATCGAATCTTTGAACGCACATTGCGCCCGCCAGTATTCTGGCGGGCATGCCTGTTCGAGCGTCATTTCAACCATCAAGCCCCCGGGCTTGTGCTGGGGACCTGCGGCTG--CCGCAGGCCCTGAAAACCAGTGGCGGGCTCGCTGT-CACACCGGGCGTAGTAGATTTTATCTCGCT----CAGGGCGTGCTGCGGGCTCCGGCCGTTAAAAAGCCTTTTT------CACCCAA-GGTTGACCTCGGATCAGGTAGGAAGACCCGCTGAACTTAAGCATATCAATAAG-CGGAGGAAAAGAAACCAACAGGGATTGCCCCAGTAACGGCGAGTGAAGCGGCAACAGCTCAAATTTGAAATCTGGCCTCGGCCCGAGTTGTAATTTGCAGAGGAAGCTTTAGGCGCGGCACCATCTGAGTCCCCTGGAACGGGGCGCCACAGAGGGTGAGAGCCCCGTATAGATGGACGCCTAGCCTGTGTAAAGCTCCTTCGACGAGTCGAGTAGTTTGGGAATGCTGCTCAAAATGGGAGGTAAATTTCTTCTAAAGCTAAATACCGGCCAGAGACCGATAGCGCACAAGTAGAGTGATCGAAAGATGAAAAGCACTTTGAAAAGAGGGTTAAATAGCACGTGAAATTGTTGAAAGGGAAGCGCTTGTGACCAGACTTGCGCCGGGCGGATCATCCGGTGTTCTCACCGGTGCACTCCGCCCGGCTCAGGCCAGCATCGGTTCTCGCGGGGGGATAAAGGCACCGGGAACGTAGCTCCCTCGGGAGTGTTATAGCCCGGGGCGTAATGCCCTCGCGGGGACCGAGGACCGCGCTTCGGCAAGGATGCTGGTCACACTGGTTCAGGGGCTGCGGAGGAAGAACGTCATCTCGTTCGAGGTTTCGCTCGTCCGGGACATTCGCGACCGCGAGTTCAAGATCTTTTCCGACGCCGGCCGTGTCATGAGGCCGCTGTATACCGTGGAACAAGAGAAGAAT---GGAGAGAGCGGCGCCGAGATGGGGCAGCTTATCCTCAACAAGGAGCACATAGCGAGGTTGGAGGCGGACAAGGAGTTGGGCAAATATCACCCCGACTACTGGGGCTGGCAGGGCTTGTTGAAGTCGGGTGCTATCGAGTACCTCGATGCCGAAGAGGAGGAGACGGTCATGATCAGCATGACTCCCGAGGACCTCGACAAGTTCCGCTACCGCAAAATGGGGTTCATCGTGGAAGACAACTCTGGCCAAGGTAACAACAGGATCAAGACGAAGCCGAACCCGGCTACGCACATGTACACCCACTGCGAGATCCACCCCAGCATGTTGCTCGGCATCTGCGCCAGCATCATCCCCTTCCCCGACCACAACCAGACGCGTCCTGGGAAGC--TTTTTT-GGGCCCCTGATCTACCCCAC--ACATTGGGACGAC-GTCGTTCCACCA---GCTACCC--GACCAC----CCGACCGAC-----GCGAGGGCGATAGC-GGCGCTGCCACGGTGG-AATGAACAAAATGCTGACTCTGTCTTTCTACTACAGGTTCACCTCCAGACCGGCCAATGCGTAAGTCGATTTGAATACAACACGG------------TGACCGA----CCGGATGGTGCGGGCGGACTGAC-AGCACG--CTCTC-AGGGTAACCAAATTGGTGCCGCTTTCTGGTATGTTTGACCTCCAACATCAGTATACTGACGTGTC-GAGAACAACCAAGACTGACTTCCCCTCCAGGCAGACCATTTCCGGCGAGCACGGCCTCGATAGCAATGGCGTGTACGTGACGGTCACCAATCCCCGGCCGATGGAT---CATCGCTCACCGCTTCGATAGGTACAACGGCACCTCCGAGCTCCAGCTCGAGCGCATGAACGTCTATTTCAACGAGGTGAGTCGGACTCCAGA-CCTT---CATCGGCTAATCAAGCGGTTGTGTTCGCTGACAA-CAGTTGTTCTGCAGGCGTCCGGCAACAAGTATGTCCCTCGCGCCGTCCTCGTCGACTTGGAGCCCGGCACCATGGATGCCGTCCGCGCCGGTCCTTTCGGCCAGCTCTTCCGCCCCGACAACTT

>Botryotrichum_iranicum_ABRIICC_10152

?????AGAGTTGCAAAACT-CCCTAAACCATTGTGAACGTTACCTTCAAACCGTTGCTTCGGCGGGCGGC-CCGGG-----TCC-GCCCGGTGCCCCCTGGCCCCCT----AGCGGGGCGCCCGCCGGAGG-AAACCCAACTCTTGAT-TATTATGGCCTCTCTGAGTCTTCTGTACTGAATAAGTCAAAACTTTCAACAACGGATCTCTTGGTTCTGGCATCGATGAAGAACGCAGCGAAATGCGATAAGTAATGTGAATTGCAGAATTCAGTGAATCATCGAATCTTTGAACGCACATTGCGCCCGCCAGTATTCTGGCGGGCATGCCTGTTCGAGCGTCATTTCAACCATCAAG-CCCCAGGCTTGTGTTGGGGACCTGCGGCTG--CCGCAGGCCCTGAAAACCAGTGGCGGGCTCGCTGT-CACACCGGGCGTAGTAGATTTTATCTCGCT----CAGGGCGTGCTGCGGGTTCCGGCCGTT-AAAAGCCTTTTT------TACCCAA-GGTTGACCTCGGATCAGGTAGGAATACCCGCTGAACTTAAGCATATCAATAAG-CGGAGGAAAAGAAACCAAC?????????????????????????????????????????????TTGAAATCTGGCTTCGGCCCGAGTTGTAATTTGCAGAGGAAGCTTTAGGCGCGGCACCATCTGAGTCCCCTGGAACGGGGCGCCACAGAGGGTGAGAGCCCCGTATAGATGGACGCCTAGCCTGTGTAAAGCTCCTTCGACGAGTCGAGTAGTTTGGGAATGCTGCTCAAAATGGGAGGTAAATTTCTTCTAAAGCTAAATACCGGCCAGAGACCGATAGCGCACAAGTAGAGTGATCGAAAGATGAAAAGCACTTTGAAAAGAGGGTTAAATAGCACGTGAAATTGTTGAAAGGGAAGCGCTTGTGACCAGACTTGCGCCGGGCGGATCATCCGGTGTTCTCACCGGTGCACTCCGCCCGGCTCAGGCCAGCATCGGTTCTCGTGGGGGGATAAAGGCACCGGGAACGTAGCTCCTCCGGGAGTGTTATAGCCCGGGGCGTAATGCCCTCGCGGGGACCGAGGACCGCGCATCTGCAAGGATG?????????????????????????????????????????????????????????????????????????????????????????????????????????????????????????????????????????????????????????????????????????????????????????????????????????????????????????????????????????????????????????????????????????????????????????????????????????????????????????????????????????????????????????????????????????????????????????????????????????????????????????????????????????????????????????????????????????????????????????????????????????????????????????????????????????????ACGCGTCTGGGATGC---TTTTCC-GAGACCCTGATCTACCCCAC--ACATCGGGACGAC-GTCGTTCCACCA----------------GC----CCGACCGGC-----GCGAGAGCAGTCGC-GGCGCTGCCATGATGG-AATGTACACGATGCTGACTCTATCTTTCTACTACAGGTCCACCTCCAGACCGGCCAATGCGTAAGTTGGATCGATTCGAACACGG------------CGACCGA----CCAGATGGAGCGGATGGACTGACAAACAAC--CTCTCTAGGGTAACCAAATTGGTGCCGCTTTCTGGTATGTTCTGCCC-CAACATCAA-GCAATGACGTGTC-AAGACCAAGCAAGACTGACTTCCACCCCAGGCAGACCATTTCCGGCGAGCACGGCCTCGACAGCAATGGCGTGTATGTGACCGTCGCCGATTCCCGGCCGATGAAT--CCCTCGCTCACCGCTTCGATAGGTACAACGGCACCTCCGAGCTCCAGCTCGAGCGCATGAACGTCTACTTCAACGAGGTGAGTCGGGCGCCGTACGCCT-----TCAGCCAGCCAAACGGCCGTCATCGCTGACAA-CAAATATTCTGCAGGCGTCCGGCAACAAGTATGTCCCCCGTGCCGTCCTTGTCGACTTGGAGCCCGGCACCATGGATGCCGTCCGCGCCGGTCCTTTCGGCCAGCTCTTCCGCCCCGACAACTT

>Botryotrichum_luteum_KNUF_22_25JW

ATTACAGAGTTGCAAAACT-CCCTAAACCATTGTGAACGTTACCTTCAAACCGTTGCTTCGGCGGGCGGC-CCGGG-----TCC-GCCCGGTGCCCCCTGGCCCCCT----AGCGGGGCGCCCGCCGGAGG-AAACCCAACTCTTGAT-TATTATGGCCTCTCTGAGTCTTCTGTACTGAATAAGTCAAAACTTTCAACAACGGATCTCTTGGTTCTGGCATCGATGAAGAACGCAGCGAAATGCGATAAGTAATGTGAATTGCAGAATTCAGTGAATCATCGAATCTTTGAACGCACATTGCGCCCGCCAGTATTCTGGCGGGCATGCCTGTTCGAGCGTCATTTCAACCATCAAG-CCCCAGGCTTGTGTTGGGGACCTGCGGCTG--CCGCAGGCCCTGAAAACCAGTGGCGGGCTCGCTGT-CACACCGGGCGTAGTAGATTTTATCTCGCT----CAGGGCGTGCTGCGGGTTCCGGCCGTTAAAAAGCCTTTTT------TACCCAA-GGTTGACCTCGGATCAGGTAGGAATACCCGCTGAACTTAAGCATATC?????????GAGGAAAAGAAACCAACAGGGATTGCCCCAGTAACGGCGAGTGAAGCGGCAACAGCTCAAATTTGAAATCTGGCTTCGGCCCGAGTTGTAATTTGCAGAGGAAGCTTTAGGCGCGGCACCATCTGAGTCCCCTGGAACGGGGCGCCACAGAGGGTGAGAGCCCCGTATAGATGGACGCCTAGCCTGTGTAAAGCTCCTTCGACGAGTCGAGTAGTTTGGGAATGCTGCTCAAAATGGGAGGTAAATTTCTTCTAAAGCTAAATACCGGCCAGAGACCGATAGCGCACAAGTAGAGTGATCGAAAGATGAAAAGCACTTTGAAAAGAGGGTTAAATAGCACGTGAAATTGTTGAAAGGGAAGCGCTTGTGACCAGACTTGCGCCGGGCGGATCATCCGGTGTTCTCACCGGTGCACTCCGCCCGGCTCAGGCCAGCATCGGTTCTCGTGGGGGGATAAAGGCACCGGGAACGTAGCTCCTCCGGGAGTGTTATAGCCCGGGGCGTAATGCCCTCGCGGGGACCGAGGACCGCGCATCTGCAAGGATGCTGGTCACCCTGGTTCAAGGGCTGCGGAGGAAGAACGTCATCTCGTTCGAGGTTTCACTCGTCCGGGACATTCGTGACCGCGAGTTCAAGATTTTCTCCGACGCTGGCCGTGTCATGAGGCCGCTGTTCACCGTAGAACAGGAGAAGAAC---GGAGAGAGCGGCGCAGAGATGGGCCAGCTTATTCTCAACAAGGACCACATCACGAGGTTGGAGGCGGACAAGGAGTTGGGCAAGTATCACCCCGACTACTGGGGCTGGCAGGGCTTGCTGAAGTCGGGCGCCATCGAGTACCTCGATGCCGAAGAGGAGGAGACGGTCATGATCAGCATGACTCCCGAGGACCTCGACAAGTTCCGTTACCGCAAAATGGGGTTCATCGTGGAAGACAACTCTGGCCAAGGTAACAACAGGATCAAGACAAAGCCAAACCCGGCTACGCACATGTATACCCACTGCGAAATCCACCCCAGCATGTTGCTCGGCATCTGCGCCAGCATCATTCCCTTCCCCGACCACAACCAG???????????????????????????????????????????????????????ACGAC-GTCGTTCCACCA----------------GC----CCGACCGGC-----GCGAGAGCGGTAGC-GGCGCTGCCATGATGG-AATGTACACGATGCTGACTTTATCTTTCCACTACAGGTCCACCTCCAGACCGGCCAATGCGTAAGTTGGATCGATTCGAACACGG------------CGACCGA----CCAGATGGTGCGGGTGGACTGAC-AGCAAC--CTCTCTAGGGTAACCAAATCGGTGCCGCTTTCTGGTATGTTCGGCCT-CATCATCAA-GCAATGACGTGTC-AAGACCGATCAAGACTGACTTCCCCTCCAGGCAGACCATTTCCGGCGAGCACGGCCTCGACAGCAATGGCGTGTATGTGACCGTCGCCGATTCCCGGTCGATGAAT--CCCCCGCTCACCGCTTCGATAGGTACAACGGCACCTCCGAGCTCCAGCTCGAGCGCATGAACGTCTACTTCAACGAGGTGAGTCGGCCCCGTAC-GCCT-----GAGGGCAGCCAGATAGCCGTAGTCGCTGACGA-CAGATATTCTGCAGGCGTCCGGCAACAAGTATGTCCCTCGTGCCGTCCTTGTCGACTTGGAGCCCGGCACCATGGATGCCGTCCGCGCCGGTCCTTTCGGCCAGCTCTTCCGCCCCGACAACTT

>Botryotrichum_murorum_CBS_163_52

ATTACAGAGTTGCAAAACT-CCC-AAACCATTGTGAACGTTACCTTCAAACCGTTGCTTCGGCGGGCGGC-CCGGG-----TCC-GCCCGGTGCCCCCTGGCCCCCT----CGCGGGGCGCCCGCCGGAGG-AAACCCAACTCTTGATACATTATGGCCTCTCTGAGTCTTCTGTACTGAATAAGTCAAAACTTTCAACAACGGATCTCTTGGTTCTGGCATCGATGAAGAACGCAGCGAAATGCGATAAGTAATGTGAATTGCAGAATTCAGTGAATCATCGAATCTTTGAACGCACATTGCGCCCGCCAGTATTCTGGCGGGCATGCCTGTTCGAGCGTCATTTCAACCATCAAG-CCCCAGGCTTGTGTTGGGGACCTGCGGCTG--CCGCAGGCCCTGAAAACCAGTGGCGGGCTCGCTGT-CACACCGGGCGTAGTAGATTTTATCTCGCT----CAGGGCGTGCTGCGGGTTCCGGCCGTTAAAAAGCCTTTTT------TACCCAA-GGTTGACCTCGGATCAGGTAGGAAGACCCGCTGAACTTAAGCATATCAATAAG-CGGAGGAAAAGAAACCAACAGGGATTGCCCCAGTAACGGCGAGTGAAGCGGCAACAGCTCAAATTTGAAATCTGGCTTCGGCCCGAGTTGTAATTTGCAGAGGAAGCTTTAGGCGCGGCACCATCTGAGTCCCCTGGAACGGGGCGCCACAGAGGGTGAGAGCCCCGTATAGATGGACGCCTAGCCTGTGTAAAGCTCCTTCGACGAGTCGAGTAGTTTGGGAATGCTGCTCTAAATGGGAGGTAAATTTCTTCTAAAGCTAAATACCGGCCAGAGACCGATAGCGCACAAGTAGAGTGATCGAAAGATGAAAAGCACTTTGAAAAGAGGGTTAAATAGCACGTGAAATTGTTGAAAGGGAAGCGCTTGTGACCAGACTTGCGCCGGGCGGATCATCCGGTGTTCTCACCGGTGCACTCCGCCCGGCTCAGGCCAGCATCGGTTCTCGTGGGGGGACAAAGGTCCCGGGAACGTAGCTCCTCCGGGAGTGTTATAGCCCGGGGCGTAATGCCCTCGCGGGGACCGAGGACCGCGCTTCTGCAAGGATGTTGGTCACCCTGGTTCAGGGGCTGCGGAGGAAGAACGTCATCTCGTTCGAGGTTTCGCTCGTCCGCGACATCCGCGACCGCGAGTTCAAGATCTTCTCCGATGCGGGTCGTGTCATGAGGCCGTTGTTCACCGTGGAACAAGAGAAGAAC---GGAGAGAGCGGTGTTGAGATGGGCCAGCTTATTCTCAAAAAGGAGCACATCACAAGGTTGGAGGCGGACAAGGAGTTGGGCAAATATCACCCCGACTACTGGGGCTGGCAGGGCTTGCTGAAGTCGGGTGCTATCGAGTACCTCGACGCCGAGGAGGAGGAGACGGTCATGATCAGCATGACGCCCGAGGACCTCGACAAGTTCCGTTACCGCAAAATGGGGTTCATCGTGGAAGACAACTCTGGCCAAGGTAACAACAGGATCAAGACAAAGCCAAACCCGGCTACGCACATGTACACCCACTGCGAGATCCACCCCAGCATGTTGCTCGGCATCTGCGCCAGCATCATCCCTTTCCCCGACCACAACCAGACGCGTCCGGGAAGCT--TTTTTT-GGGCCCCTGATCTACCCCAC--ACATTGGGACGAC-GTCGTTCCACCA---GCTCCCC--GACCAC----CCGACCGAC-GCGAGCGAGGGCGATAGC-GGCGCTGCCATGATGG-AATGAACACAGTACTGACTTTGTCTTTTTACTACAGGTCCATCTTCAGACCGGCCAATGCGTAAGTGGGATCGACTCGAACACGG------------CGACCGA----CCGGATGGTGCGGGTGGACTGAC-AGCAAG--CTCTCTAGGGTAACCAAATTGGTGCCGCTTTCTGGTATGTTCAGCCT-CAACACCAG-GCACTGACGTGTC-AAGACCGACCAAGACTGACTTCCTCTCCAGGCAGACCATTTCCGGCGAGCACGGCCTCGACAGCAATGGCGTGTACGTGACGGGCGCCGACTCCCGACCAATGATC--CCCTCGCTCACCGATTCGATAGGTACAACGGCACCTCGGAGCTCCAGCTCGAGCGCATGAACGTCTACTTCAACGAGGTGAGTCGGACACATGCAGCCTT--CATCAGGCAACCGAACGTTCCTGGTTGCTGACAA-CAGAT-TTCTACAGGCCGCCGGCAACAAGTATGTTCCCCGCGCCGTCCTGGTCGACTTGGAGCCCGGCACCATGGATGCCGTCCGCGCCGGTCCCTTCGGCCAGCTCTTCCGCCCCAACAACTT

>Botryotrichum_peruvianum_CBS_460_90

ATTACAGAGTTGCAAAACT-CCCTAAACCATTGTGAACGTTACCTTCAAACCGTTGCTTCGGCGGGCGGC-CCGGG-----TCC-GCCCGGTGCCCCCTGGCCCCCT----AGCGGGGCGCCCGCCGGAGG-AAACCCAACTCTTGAT-TATTATGGCCTCTCTGAGTCTTCTGTACTGAATAAGTCAAAACTTTCAACAACGGATCTCTTGGTTCTGGCATCGATGAAGAACGCAGCGAAATGCGATAAGTAATGTGAATTGCAGAATTCAGTGAATCATCGAATCTTTGAACGCACATTGCGCCCGCCAGTATTCTGGCGGGCATGCCTGTTCGAGCGTCATTTCAACCATCAAG-CCCCGGGCTTGTGTTGGGGACCTGCGGCTG--CCGCAGGCCCTGAAAACCAGTGGCGGGCTCGCTGT-CACACCGGGCGTAGTAGATTTTATCTCGCT----CAGGGCGTGCTGCGGGTTCCGGCCGTTAAAAAGCCTTTTT------TACCCAA-GGTTGACCTCGGATCAGGTAGGAAGACCCGCTGAACTTAAGCATATCAATAAG-CGGAGGAAAAGAAACCAACAGGGATTGCCCTAGTAACGGCGAGTGAAGCGGCAACAGCTCAAATTTGAAATCTGGCTTCGGCCCGAGTTGTAATTTGCAGAGGAAGCTTTAGGCGCGGCACCATCTGAGTCCCCTGGAACGGGGCGCCACAGAGGGTGAGAGCCCCGTATAGATGGACGCCTAGCCTGTGTAAAGCTCCTTCGACGAGTCGAGTAGTTTGGGAATGCTGCTCAAAATGGGAGGTAAATTTCTTCTAAAGCTAAATACCGGCCAGAGACCGATAGCGCACAAGTAGAGTGATCGAAAGATGAAAAGCACTTTGAAAAGAGGGTTAAATAGCACGTGAAATTGTTGAAAGGGAAGCGCTTGTGACCAGACTTGCGCCGGGCGGATCATCCGGTGTTCTCACCGGTGCACTCCGCCCGGCTCAGGCCAGCATCGGTTCTCGTGGGGGGATAAAGGCACCGGGAACGTAGCTCCTCCGGGAGTGTTATAGCCCGGGGCGTAATGCCCTCGCGGGGACCGAGGACCGCGCTTCTGCAAGGATGCTGGTCACCCTGGTTCAGGGGTTGCGGAGGAAGAACGTCATCTCGTTCGAGGTTTCACTCGTCCGGGACATTCGCGACCGCGAGTTCAAGATCTTCTCCGACGCTGGCCGTGTCATGAGGCCGCTGTTCACCGTAGAACAGGAAAAGAAC---GGGGAGAGCGGCGCAGAGATGGGCCAGCTTATTCTCAACAAGGATCATATTGCGAGATTGGAGGCGGACAAGGAGTTGGGCAAGTATCACCCCGACTACTGGGGCTGGCAGGGCTTGTTGAAGTCGGGTGCTATCGAGTACCTCGATGCCGAAGAGGAGGAGACGGTCATGATCAGCATGACTCCCGAGGACCTCGACAAGTTCCGTTACCGCAAAATGGGGTTCATCGTGGAAGACAACTCTGGCCAAGGTAACAACAGGATCAAGACGAAGCCGAACCCGGCTACGCACATGTACACTCACTGCGAGATCCACCCCAGTATGTTGCTCGGCATCTGCGCCAGCATCATCCCCTTCCCGGACCACAATCAGACGCGTCTGGGATGCT--TTTTTG-CGACCTCCGATCTACCCCAC--ACGCTGGGACGAC-GTCGTTCCACCA---GTTCCCCCTGACCAC----CCGACCGAC-----GCGAGAGCGGTAGC-GGCGCTGTCATCATGG-AATGTACACGATGCTGACTCTGTCTTTCTCCTACAGGTTCACCTCCAGACCGGTCAATGCGTAAGTTGGATCGATTCGAACATGG--------------ACCGA----CTGGATGGTGCGGGTGGACTGAC-AACAGC--CTCTCTAGGGTAACCAAATCGGTGCCGCTTTCTGGTATGTTCGGCCT-CAACATCAA-GCAATGACGTGTC-GAGACCGATCAAGACTGACATTCCCTCCAGGCAGACTATTTCCGGCGAGCACGGCCTCGACAGCAATGGCGTGTACGTGACTGTCGCCGATTCCCGGCCGATGAATACCCCCCACTCACCGCTTCGATAGATACAACGGCACCTCCGAGCTCCAGCTCGAGCGCATGAACGTCTACTTCAACGAGGTAAGTCGGGCCCATACACCTT-----CAAGGTAGCTAGACGGCCATGGTCGCTGACGA-CAGATATTCCACAGGCGTCCGGCAACAAGTATGTCCCTCGTGCCGTCCTTGTCGACTTGGAGCCCGGCACCATGGATGCCGTCCGCGCCGGTCCTTTCGGCCAGCTCTTCCGCCACGACAACTT

>Botryotrichum_piluliferum_CBS_654_79

ATTACAGAGTTGCAAAACT-CCCTAAACCATTGTGAACGTTACCTTCAAACCGTTGCTTCGGCGGGCGGC-CCGGG-----TCC-GCCCGGTGCCCCCTGGCCCCCT----AGCGGGGCGCCCGCCGGAGGAAAACCCAACTCTTGAT-TATTATGGCCTCTCTGAGTCTTCTGTACTGAATAAGTCAAAACTTTCAACAACGGATCTCTTGGTTCTGGCATCGATGAAGAACGCAGCGAAATGCGATAAGTAATGTGAATTGCAGAATTCAGTGAATCATCGAATCTTTGAACGCACATTGCGCCCGCCAGTATTCTGGCGGGCATGCCTGTTCGAGCGTCATTTCAACCATCAAG-CCCCGCGCTTGTGTTGGGGACCTGCGGCTG--CCGCAGGCCCTGAAAACCAGTGGCGGGCTCGCTGT-CACACCGGGCGTAGTAGATTTTATCTCGCT----CAGGGCGTGCTGCGGGTTCCGGCCGTTAAAAAGCCTTTTT------TACCCAA-GGTTGACCTCGGATCAGGTAGGAATACCCGCTGAACTTAAGCATATCAATAAG-CGGAGGAAAAGAAACCAACAGGGATTGCCTCAGTAACGGCGAGTGAAGCGGCAACAGCTCAAATTTGAAATCTGGCTTCGGCCCGAGTTGTAATTTGCAGAGGAAGCTTTAGGCGCGGCACCATCTGAGTCCCCTGGAACGGGGCGCCACAGAGGGTGAGAGCCCCGTATAGATGGACGCCTAGCCTGTGTAAAGCTCCTTCGACGAGTCGAGTAGTTTGGGAATGCTGCTCAAAATGGGAGGTAAATTTCTTCTAAAGCTAAATACCGGCCAGAGACCGATAGCGCACAAGTAGAGTGATCGAAAGATGAAAAGCACTTTGAAAAGAGGGTTAAATAGCACGTGAAATTGTTGAAAGGGAAGCGCTTGTGACCAGACTTGCGCCGGGCGGATCATCCGGTGTTCTCACCGGTGCACTCCGCCCGGCTCAGGCCAGCATCGGTTCTCGTGGGGGGATAAAGGCACCGGGAACGTAGCTCCTCCGGGAGTGTTATAGCCCGGGGCGTAATGCCCTCGCGGGGACCGAGGACCGCGCATCTGCAAGGATGCTGGTCACCCTGGTTCAGGGTTTGCGGAGGAAGAACGTCATCTCGTTCGAGGTTTCGCTCGTCCGGGACATTCGCGACCGCGAGTTCAAGATTTTCTCCGACGCGGGCCGTGTCATGAGGCCGTTGTTCACGGTAGAACAAGAGAAGAAC---GGAGAGAGCGGCGCAGAAATGGGGCAGCTTATTCTCAACAAGGAGCACATTACGAGATTGGAGGCGGACAAGGAGTTGGGCAAGTATCACCCCGACTACTGGGGCTGGCAGGGCTTGTTGAAGTCGGGCGCTATCGAGTACCTCGATGCCGAAGAAGAGGAGACGGTCATGATCAGCATGACTCCCGAGGACCTCGACAAGTTCCGTTACCGCAAGATGGGGTTCATCGTTGAAGACAACTCTGGCCAAGGTAATAACAGGATCAAGACAAAGCCGAACCCGGCCACGCACATGTACACCCACTGCGAGATCCACCCCAGCATGTTGCTCGGCATCTGCGCCAGCATCATCCCCTTCCCCGACCACAACCAGACGCGTCTGGGATGC---TTTTTG-GGGACCCTGATCTACCCCAC--ACACTGGGACGAC----------------GTTCCCC--GACCAC----CCGACCGAC-----GCGAGAGCGGTAGC-GGCGCTGCCATGATGA-AATGTACACGATGCTGACTTTATCTTTCTCCTACAGGTCCACCTCCAGACCGGCCAATGCGTAAGTTGGATCGACTCGAACACGG------------CGACCGA----CCGGATGGGGCGGATGGACTGACAAGCAAC--CTCTCTAGGGTAACCAAATCGGTGCCGCTTTCTGGTATGTCCGGCCT-CAACATCAA-GCAATGACGTGTC-AAGACGGATCAAGACTGACTTCCCCTCCAGGCAGACCATTTCCGGCGAGCACGGCCTCGACGCCAATGGCGTGTATGTGACCGCCGCCGATTCCCGGCCGATGAAT--CCCCCGCTCACCGCTTCGATAGGTACAACGGCACCTCCGAGCTCCAGCTCGAGCGCATGAACGTCTACTTCAACGAGGTGAGTCGGGCCCGTACGGCCT-----TGAGGCAGCCAGAC------GGTCGCTGACGA-CAGATATTCTACAGGCGGCAGGCAACAAGTATGTCCCTCGTGCCGTCCTTGTCGACTTGGAGCCCGGCACCATGGATGCCGTCCGCGCCGGTCCTTTCGGCCAGCTCTTTCGCCCCGACAACTT

>Botryotrichum_pseudomurorum_FMR_19917

ATTACAGAGTTGCAAAACTCCCCTAAACCATTGTGAACGTTACCTTCAAACCGTTGCTTCGGCGGGCGGCACCGGG-----TCC-GCCCTGTGCCCCCTGGCCCCCC----AGCGGGGCGCCCGCCGGAGG-AAACCCAACTCTTGATACATTATGGCCTCTCTGAGTCTTCTGTACTGAATAAGTCAAAACTTTCAACAACGGATCTCTTGGTTCTGGCATCGATGAAGAACGCAGCGAAATGCGATAAGTAATGTGAATTGCAGAATTCAGTGAATCATCGAATCTTTGAACGCACATTGCGCCCGCCAGTATTCTGGCGGGCATGCCTGTTCGAGCGTCATTTCAACCATCAAG-CCCCAGGCTTGTGTTGGGGACCTGCGGCTG--CCGCAGGCCCTGAAAACCAGTGGCGGGCTCGCTGT-CACACCGGGCGTAGTAGATTATATCTCGCT----CAGGGCGTGCTGCGGGTTCCGGCCGTTAAAAAGCCTTTTT------TACCCAA-GGTTGACCTCGGATCAGGTAGGAAGACCCGCTGAACTTAAGCATATCAATAAG-CGGAGGAAAAGAAACCAACAGGGATTGCCCTAGTAACGGCGAGTGAAGCGGCAACAGCTCAAATTTGAAATCTGGCTTCGGCCCGAGTTGTAATTTGCAGAGGAAGCTTTAGGCGCGGCACCATCTGAGTCCCCTGGAACGGGGCGCCACAGAGGGTGAGAGCCCCGTATAGATGGACGCCTAGCCTGTGTAAAGCTCCTTCGACGAGTCGAGTAGTTTGGGAATGCTGCTCAAAATGGGAGGTAAATTTCTTCTAAAGCTAAATACCGGCCAGAGACCGATAGCGCACAAGTAGAGTGATCGAAAGATGAAAAGCACTTTGAAAAGAGGGTTAAATAGCACGTGAAATTGTTGAAAGGGAAGCGCTTGTGACCAGACTTGCGCCGGGCGGATCATCCGGTGTTCTCACCGGTGCACTCCGCCCGGCTCAGGCCAGCATCGGTTCTCGTGGGGGGATAAAGGCACCGGGAACGTAGCTCCTTCGGGAGTGTTATAGCCCGGGGCGTAATGCCCTCGCGGGGACCGAGGACCGCGCATCTGCAAGGATGCTGGTCACTCTGGTTCAAGGGCTGCGGAGGAAGAACGTCATCTCGTTCGAGGTTTCACTCGTCCGGGACATTCGCGACCGCGAGTTCAAGATCTTTTCCGATGCCGGCCGTGTCATGAGGCCGCTGTATACCGTGGAGCAAGAGAAGAAT---GGGGAGAGCGGCGCCGAGATGGGGCAGCTGATCCTCAACAAAGATCACATTACGAGGTTGGAGGCGGACAAGGAGTTGGGCAAATATCATCCCGACTACTGGGGCTGGCAGGGCTTGTTGAAGTCGGGTGCGATCGAGTACCTCGATGCCGAAGAGGAGGAGACGGTCATGATCAGCATGACGCCCGAGGACCTCGACAAGTTCCGTTACCGCAAAATGGGTTTCATCGTGGAAGACAACTCTGGCCAAGGTAACAACAGGATCAAGACAAAGCCAAACCCGGCTACGCACATGTACACCCACTGCGAGATCCACCCCAGTATGTTGCTCGGCATCTGCGCCAGCATCATTCCCTTCCCCGACC????????ACGCGTC-GGGAAGCT--TTTTTT-GGGCCCCTGATCTACCCCAC--ACATTGGGACGAC-GTCGTTCCACCA---GCTACCC--GACCAC----CCGACCAAC-----GCGAGGGCGATGGC-CTCGCTGCCATGATGG-AATGAACACGATGCTGACTCTGTCTTTCTGCTACAGGTTCACCTCCAGACCGGCCAATGCGTAAGTTGGATCGATTCGCACACGG------------CGACCGA----CCGGATGGTGCGGGTGGACTGAC-AGCAAG--CTCTCCAGGGTAACCAAATCGGTGCCGCTTTCTGGTATGTTCGGCCT-CAACATCAA-GCACTGACGTGTC-AAGACCGATCAAGACTGACTTCCTCTCCAGGCAGACCATTTCCGGCGAGCACGGCCTCGACAGCAATGGCGTGTACGTGACCGTCGCCGATTCCCGGCCGATGAAT---CCCCGCTCACCGCTTTGATAGGTACAACGGCACCTCCGAGCTCCAGCTCGAGCGCATGAACGTCTACTTCAATGAGGTGAGTCGAGCCCATACCATACACCTTGGAGCCAGCCAAACCGTCGTGGTTGCTGACGA-CAGATACTCTACAGGCGTCCGGCAACAAGTATGTCCCTCGTGCCGTCCTTGTCGACTTGGAGCCCGGCACCATGGATGCCGTCCGCGCCGGTCCCTTCGGCCAGCTCTTCCGCCCCGACAACTT

>Botryotrichum_spirotrichum_CBS_211_55

ATTACAGAGTTGCAAAACT-CCC-AAACCATTGTGAACGTTACCTTCAAACCGTTGCTTCGGCGGGCGGCGCCGGG-----TCC-GCCCGGTGCCCCTCGGCCCCCT----TGCGGGGCGCCCGCCGGAGG-AAACCCAACTCTTGAT-TATCATGGCCTCTCTGAGTCTTCTGTACTGAATAAGTCAAAACTTTCAACAACGGATCTCTTGGTTCTGGCATCGATGAAGAACGCAGCGAAATGCGATAAGTAATGTGAATTGCAGAATTCAGTGAATCATCGAATCTTTGAACGCACATTGCGCCCGCCAGTATTCTGGCGGGCATGCCTGTTCGAGCGTCATTTCAACCATCAAG-CCCCAGGCTTGTGTTGGGGACCTGCGGCTG--CCGCAGGCCCTGAAAACCAGTGGCGGGCTCGCTGT-CACACCGGGCGTAGTAGATTTTATCTCGCT----CAGGGCGTGCTGCGGGTTCCGGCCGTTAAAAAGCCTTTTT------TACCCAA-GGTTGACCTCGGATCAGGTAGGAATACCCGCTGAACTTAAGCATATCAATAAG-CGGAGGAAAAGAAACCAACAGGGATTGCCCCAGTAACGGCGAGTGAAGCGGCAACAGCTCAAATTTGAAATCTGGCTTCGGCCCGAGTTGTAATTTGCAGAGGAAGCTTTAGGCGCGGCACCATCTGAGTCCCCTGGAACGGGGCGCCAAAGAGGGTGAGAGCCCCGTAGAGATGGACGCCTAGCCTGTGTAAAGCTCCTTCGACGAGTCGAGTAGTTTGGGAATGCTGCTCAAAATGGGAGGTAAATTTCTTCTAAAGCTAAATACCGGCCAGAGACCGATAGCGCACAAGTAGAGTGATCGAAAGATGAAAAGCACTTTGAAAAGAGGGTTAAATAGCACGTGAAATTGTTGAAAGGGAAGCGCTTGTGACCAGACTTGCGCCGGGCAGATCATCCGGTGTTCTCACCGGTGCACTCTGCCCGGCTCAGGCCAGCATCGGTTCTCGCGGGGGGATAAAGGCGCCGGGAACGTAGCTCCTCCGGGAGTGTTATAGCCCGGCGTGTAATGCCCTCGCGGGGACCGAGGACCGCGCATCTGCAAGGATGCTCGTCACGTTGGTGCAGGGGCTGCGGAGGAAGAACGTCATCTCGTTCGAGGTCTCGCTCGTCCGCGACATTCGCGACCGCGAGTTCAAGATCTTCTCTGACGCGGGCCGTGTCATGAGGCCGCTCTTCACGGTGGAGCAGGAGAAGAAC---GGGGAGAGCGGGGCCGAGATGGGCCAGCTGATCCTCAACAAGGAGCATATTACGAGGTTGGAGGCGGACAAGGAATTGGGCAAGTACCACCCCGACTACTGGGGCTGGCCGGGCTTGTTGAAGTCGGGTGCTATCGAGTACCTCGACGCCGAGGAGGAGGAGACGGTCATGATCAGTATGACCCCCGAGGACCTCGACAAGTTCCGTTACCGCAAGATGGGCTTCATCGTGGAAGACAACTCTGGACAAGGTAACAACAGGATCAAGACAAAGCCGAACCCGGCTACGCACATGTACACCCACTGCGAGATCCACCCCAGCATGTTGCTCGGCATCTGCGCCAGCATCATCCCGTTCCCCGACCACAACCAGACGCGTCTGGGAAGC----TTTTT-AGGCCCCTGATCTACCCCAC--ACGCTGGGACGAC-GTCGTTCCACCA---GCTACCC--GACCGC----CCGCCCGAC-----GCGAGGGCGATCGC-GGCGCTGCCGTGACGG-GGAGAACACGATGCTGACTCTGCCTTCCCACTACAGGTTCACCTCCAGACCGGCCAATGCGTAAGTTGGATCGATATAAACACGG------------CGACCGACCGTCCGGATGATGCGGGTGGACTGAC-AACAAG--CTCTCCAGGGTAACCAAATTGGTGCCGCTTTCTGGTATGCCCGACCA-CAACACCAG-GCACTGACGTGTC-AAGGCCGATCGAGACTGACTTCTCTCGCAGGCAGACCATTTCCGGCGAGCACGGCCTCGACAGCAATGGCGTGTACGTGACCGTCACCGGTTCCCGGCCGAAGGGT---CCCCGCTCACCGCTTCGATAGGTACAACGGCACCTCCGAGCTCCAGCTCGAGCGCATGAACGTCTACTTCAACGAGGTTAGTCGGGCTCATACGGCTT---CAGCAAGCAGCGAGACGAGTGTGGCCGCTGACAA-CACATGTTCTGCAGGCGTCCGGCAACAAGTATGTTCCTCGCGCCGTCCTGGTCGACTTGGAGCCCGGCACCATGGATGCCGTCCGCGCCGGTCCTTTCGGCCAGCTCTTCCGCCCCGACAACTT

>Botryotrichum_trichorobustum_CBS_563_67

??????????????????????????????????????????????????????????????????????????????????????????????????????????????????????????????????????????????????????????????????????????????????????????????????????????????????????????????????????????????????????????????????????????????????????????????????????????????????????????????????????????????????????????????????????????????????????????????????????????????????????????????????????????????????????????????????????????????????????????????????????????????????????????????????????????????????????????????????????????GCATATCAATAAG-CGGAGGAAAAGAAACCAACAGGGATTGCCCCAGTAACGGCGAGTGAAGCGGCAACAGCTCAAATTTGAAATCTGGCCTCGGCCCGAGTTGTAATTTGCAGAGGAAGCTTTAGGCGCGGCACCATCTGAGTCCCCTGGAACGGGGCGCCGCAGAGGGTGAGAGCCCCGTAGAGATGGACGCCTAGCCTGTGTAAAGCTCCTTCGACGAGTCGAGTAGTTTGGGAATGCTGCTCAAAATGGGAGGTAAATTTCTTCTAAAGCTAAATACCGGCCAGAGACCGATAGCGCACAAGTAGAGTGATCGAAAGATGAAAAGCACTTTGAAAAGAGGGTTAAATAGCACGTGAAATTGTTGAAAGGGAAGCGCTTGTGACCAGACTTGCGCCGGGCGGATCATCCGGTGTTCTCACCGGTGCACTCCGCCCGGCTCAGGCCAGCATCGGTTCTCGCGGGGGGATAAAGGCACCGGGAACGTAGCTCCTCCGGGAGTGTTATAGCCCGGGGCGTAATGCCCTCGCGGGGACCGAGGACCGCGCATCTGCAAGGATGCTGGTCACCCTGGTTCAAGGGCTGCGGAGGAAGAACGTCATCTCGTTTGAGGTTTCACTGGTCCGGGACATCCGTGAACGCGAGTTCAAGATTTTCTCGGATGCCGGACGTGTCATGAGGCCGCTGTTCACCGTGGAGCAAGAGAAGAAC---GGAGAGAGCGGCGTCGAGATGGGCCAGCTGATACTCAGCAAGGAGCACATTACTAGGTTGGAGGCGGACAAAGAGTTGGGCAAGTATCACCCCGACTACTGGGGTTGGCAGGGCTTGTTGAAGTCGGGCGCTATCGAGTACCTCGATGCCGAGGAGGAGGAGACGGTCATGATCAGCATGACTCCCGAGGACCTCGACAAGTTCCGTTACCGCAAGATGGGGTTCATCGTGGAAGACAACTCTGGCCAAGGTAACAACAGGATCAAGACAAAACCAAACCCAACCACGCACATGTACACCCACTGCGAGATCCACCCTAGCATGCTGCTCGGCATCTGCGCCAGCATCATTCCGTTCCCCGACCATAACCAGACGCGTCTGGGAGGC---TTTTTT-TGGGCCCCGATTTACCCCAC--ACGTTGGGACGACAGTCGTTCCACCA---GCTACCC--GAGCAC----CCGACCGAC---------GGGCGATAGCGGGCGCTGCCATGATGG-AAAGAGTACGATGCTAACACAGTCTCTCTACTACAGGTTCACCTCCAGACCGGCCAATGCGTAAGTTGAATCGATTCGAACACGGCGACCGACCGATCGACCGA----CCGGATGGCGCGGGTGGACTGAC-AGCAAG--CTCTCTAGGGCAACCAAATCGGTGCCGCCTTCTGGTATGTTTGGCCT-CGACATCAA-GCAACGACGTGTC-AAGATCGACCAAGACTGACTTCCCTTGCAGGCAGACCATTTCCGGCGAGCACGGCCTTGACGCAAGTGGCGTGTACGTGGTGACGGCCGACTCCCCGCCGATGAAC--CCCTCGCTCACCGCTTCGATAGGTACAACGGCACCTCTGAGCTCCAGCTCGAGCGCATGAACGTCTACTTCAACGAGGTGAGTTGGGCCCCGTTTACCTT--GATCAGACAACC-GACGGGCGTGATGACTGACAA-GATGTGTTCTGTAGGCGTCCGGCAACAAGTATGTCCCTCGCGCCGTCCTGGTCGACTTGGAGCCCGGCACCATGGATGCCGTCCGCGCCGGTCCCTTCGGCCAGCTCTTCCGCACCGACAACTT

>Botryotrichum_verrucosum

ATTACAGAGTTGCAAA-CT-CCCTAAACCATTGTGAACGTTACCTTCAAACCGTTGCTTCGGCGGGCGGC-CCGGG-----TCC-GCCCGGTGCCCCCTGGCCCCCT----AGCGGGGCGCCCGCCGGAGG-AAACCCAACTCTTGATACATTATGGCCTCTCTGAGTCTTCTGTACTGAATAAGTCAAAACTTTCAACAACGGATCTCTTGGTTCTGGCATCGATGAAGAACGCAGCGAAATGCGATAAGTAATGTGAATTGCAGAATTCAGTGAATCATCGAATCTTTGAACGCACATTGCGCCCGCCAGTATTCTGGCGGGCATGCCTGTTCGAGCGTCATTTCAACCATCAAG-CCCCAGGCTTGTGTTGGGGACCTGCGGCTG--CCGCAGGCCCTGAAAACCAGTGGCGGGCTCGCTGT-CACACCGGGCGTAGTAGATTTTATCTCGCT----CAGGGCGTGCTGCGGGTTCCGGCCGTTAAAAAGCCTTTTT------TACCCAA-GGTTGACCTCGGATCAGGTAGGAAGACCCGCTGAACTTAAGCATATCAATAAG-CGGAGGAAAAGAAACCAACAGGGATTGCCCCAGTAACGGCGAGTGAAGCGGCAACAGCTCAAATTTGAAATCTGGCTTCGGCCCGAGTTGTAATTTGCAGAGGAAGCTTTAGGCGCGGCACCATCTGAGTCCCCTGGAACGGGGCGCCACAGAGGGTGAGAGCCCCGTATAGATGGACGCCTAGCCTGTGTAAAGCTCCTTCGACGAGTCGAGTAGTTTGGGAATGCTGCTCAAAATGGGAGGTAAATTTCTTCTAAAGCTAAATACCGGCCAGAGACCGATAGCGCACAAGTAGAGTGATCGAAAGATGAAAAGCACTTTGAAAAGAGGGTTAAATAGCACGTGAAATTGTTGAAAGGGAAGCGCTTGTGACCAGACTTGCGCCGGGCGGATCATCCGGTGTTCTCACCGGTGCACTCCGCCCGGCTCAGGCCAGCATCGGTTCTCGTGGGGGGATAAAGGCACCGGGAACGTAGCTCCTCCGGGAGTGTTATAGCCCGGGGCGTAATGCCCTCGCGGGGACCGAGGACCGCGCATCTGCAAGGATG?????????????????????????????????????????????????????????????????????????????????????????????????????????????????????????????????????????????????????????????????????????????????????????????????????????????????????????????????????????????????????????????????????????????????????????????????????????????????????????????????????????????????????????????????????????????????????????????????????????????????????????????????????????????????????????????????????????????????????????????????????????????????????????????????????????????ACGCGTCTGGGAAAC---TTTTTT-GGGACCCTGATCTACCCCAC--ACACTGGGACGAC-GTCGTTCCACCA---GTTCCCC--GACCAC----CCGACCGAC-----GCGAGAGCGGTAGC-GGCGCTGCCATGATGG-AATGAACACGATGCTGACTCGGTCTTGCTACTACAGGTTCACCTCCAGACCGGCCAATGCGTAAGTTGGATCGATTCGAACACGG------------C-ACTGA----CCGGATGGTGCGGATGGACTGAC-AGCAAG--CTCTCTAGGGTAACCAAATCGGTGCTGCTTTCTGGTATGTTCGGCCT-CAGCATCAA-GCACTGACGTGTC-GGGACCGATCAAGACTAACTTCCCCTCCAGGCAGACCATTTCCGGCGAGCACGGCCTCGACAGCAATGGCGTGTACGTGACCGTCGCCGATTCCCGGCCGATGAAC---CCCCGCTCACCGCTTCGATAGGTACAACGGCACCTCCGAGCTCCAGCTCGAGCGCATGAACGTCTACTTCAACGAGGTGAGTCGGGTT---ACACCTT---CATCGCGTAACCAAAACGTCGAGGTCACTAACCG-CAGATGTTCTGCAGGCGTCCGGAAACAAGTATGTCCCTCGTGCCGTCCTCGTCGACTTGGAGCCCGGCACCATGGATGCCGTCCGCGCCGGTCCTTTCGGCCAGCTCTTCCGCCCCGACAACTT

>Botryotrichum_vitellinum_CBS_180_84

?TTACAGAGTTGCAAAACT-CCCTAAACCATTGTGAACGTTACCTTCAAACCGTTGCTTCGGCGGGCGGCGCCGGG-----TCC-GCCCGGCGCCCCTCGGCCCCCCCTCGCGGGGAGCGCCCGCCGGAGG-AAACCCAACTCTTGATATA-TATGGCCTCTCTGAGTCTTCTGTACTGAATAAGTCAAAACTTTCAACAACGGATCTCTTGGTTCTGGCATCGATGAAGAACGCAGCGAAATGCGATAAGTAATGTGAATTGCAGAATTCAGTGAATCATCGAATCTTTGAACGCACATTGCGCCCGCCAGTATTCTGGCGGGCATGCCTGTTCGAGCGTCATTTCAACCATCAAGCCCCCGGGCTTGTGTTGGGGACCTGCGGCTG--CCGCAGGCCCTGAAAACCAGTGGCGGGCTCGCTGT-CACACCGGGCGTAGTAG-TTTTATCTCGCT----CAGGGCGTGCTGCGGGTTCCGGCCGTT-AAAAGCCTTTTT------TACCCAA-GGTTGACCTCGGATCAGGTAGGAATACCCGCTGAACTTAAGCATATCAATAAG-CGGAGGAAAAGAAACCAACAGGGATTGCCCTAGTAACGGCGAGTGAAGCGGCAACAGCTCAAATTTGAAATCTGGCTTCGGCCCGAGTTGTAATTTGCAGAGGAAGCTTTAGGCGCGGCACCATCTGAGTCCCCTGGAACGGGGCGCCATAGAGGGTGAGAGCCCCGTAGAGATGGACGCCTAGCCTGTGTAAAGCTCCTTCGACGAGTCGAGTAGTTTGGGAATGCTGCTCAAAATGGGAGGTAAATTTCTTCTAAAGCTAAATACCGGCCAGAGACCGATAGCGCACAAGTAGAGTGATCGAAAGATGAAAAGCACTTTGAAAAGAGGGTTAAATAGCACGTGAAATTGTTGAAAGGGAAGCGCTTGTGACCAGACTTGCGCCGGGCGGATCATCCGGTGTTCTCACCGGTGCACTCCGCCCGGCTCAGGCCAGCATCGGTTCTCGCGGGGGGATAAAGGCACCGGGAACGTAGCTCCTCCGGGAGTGTTATAGCCCGGGGCGTAATGCCCTCGCGGGGACCGAGGACCGCGCATCTGCAAGGATGCTAGTCACTCTAGTGCAAGGGCTGCGGAGGAAGAATGTCATCTCGTTTGAGGTTTCGCTCGTCCGGGACATCCGCGACCGCGAGTTCAAGATCTTCTCCGATGCGGGTCGTGTCATGAGGCCGCTGTTCACCGTGGAGCAGGAAAAGAAC---GGAGACAGCGGCGCCGAGATGGGCCAACTGATCCTCAACAAGGAGCACATTACGAGGCTGGAGGCGGATAAGGAGTTGGGCAAGTACCACCCCGACTACTGGGGCTGGCAAGGGCTGCTGAAGTCGGGTGCCATCGAGTATCTCGACGCCGAGGAGGAGGAGACAGTCATGATCAGCATGACTCCCGAGGATCTCGACAAGTTCCGTTACCGCAAGATGGGGTTCATCGTGGAAGACAACTCTGGTCAAGGAAACAACAGAATCAAGACGAAGCCAAACCCCGCGACGCACATGTACACGCACTGCGAGATCCACCCCAGCATGTTGCTCGGCATCTGCGCCAGCATCATCCCGTTCCCCGACCACAACCAGACGCGTCTGGGAAGCT--TTTTCT-GGGCCCCTGATCTACCCCAC--ACATTTGGACGACAGTCGTTCCACCA---GCTCCCC--GACC------CCGACCGACGGAGGGCGATGTCGATAGC-GGCGCTGCCATGATGGAAAGGAATATGTCGCTGACACTGTCTCTCTGCTACAGGTTCACCTCCAGACCGGCCAATGCGTAAGTTGGATCGATGCGAACACGG------------CGACCGA----TCGGGTGGTGCGGGTGGACTGAC-AGCAAC--CTCTCCAGGGTAACCAAATTGGTGCCGCTTTCTGGTATGTTCGACCT-CAACATGAA-ACACTGACATGCCGAAAAACGATCAAGACTGACTTCCCCTCCAGGCAGACCATTTCCGGCGAGCACGGCCTTGACAGCAGTGGCGTGTACGTGACCGTCGCCGA-TCCCGGCCAACGAAT--CCCTCGCTCACCGCTACGATAGGTACAATGGCACCTCCGAGCTCCAGCTCGAGCGCATGAACGTCTACTTCAACGAGGTGAGTCGGGCCCAGACACCTT---AATCGAGCAATCAAACGGCCGCGGTCGCTAACAACCACACTTTCTGCAGGCGTCCGGCAACAAGTATGTCCCTCGTGCCGTCCTGGTCGACTTGGAGCCCGGCACCATGGATGCCGTCCGCGCCGGTCCCTTCGGCCAGCTCTTCCGCCCCGACAACTT

>Botryotrichum_retardatum_CBS_197_84

ATTACAGAGTTGCAAAACT-CCCTAAACCATCGTGAACGTTACCCACAAACCGTTGCTTCGGCGGGCGGCTACGGG-----CCCAGCCCGGCGCCCCCCGGCCCCCT------GCGGGCGCCCGCCGGAGG-AAACCAAACTCTTCTGACATCATGGCCTCTCTGAGTATTCTGTACTGAATAAGTCAAAACTTTCAACAACGGATCTCTTGGTTCTGGCATCGATGAAGAACGCAGCGAAATGCGATAAGTAATGTGAATTGCAGAATTCAGTGAATCATCGAATCTTTGAACGCACATTGCGCCCGCCAGTATTCTGGCGGGCATGCCTGTTCGAGCGTCATTTCAACCATCAAG-CCCACGGCTTGTGTTGGGGACCTGCGGCTG--CCGCAGGCCCTGAAAACCAGTGGCGGGCTCGCTGT-CACACCGGGCGTAGTAGATTACATCTCGCC----CAGGGCGTGCTGCGGGTTCCGGCCGTAAAAAAGCCTTTTT------CACCCAA-GGTTGACCTCGGAKCAGGTRGGAAKACSYGCTGAACTTAAGCATATCAATAAGGCGGAGGA?????????????????????????????????????????????????????????????????????????????????????????????????????????????????????????????????????????????????????????????????????????????????????????????????????????????????????????????????????????????????????????????????????????????????????????????????????????????????????????????????????????????????????????????????????????????????????????????????????????????????????????????????????????????????????????????????????????????????????????????????????????????????????????????????????????????????????????????CTGGTCAACTTAGTCCAGGGGCTGCGGAGAAAGAACGTCATTTCGTTCGAGGTCTCGCTGGTCCGCGACATCCGGGACCGCGAGTTCAAGATCTTTTCGGATGCGGGCCGCGTCATGAGGCCGCTGTTCACGGTGGAGCAAGAGATTAAC---GGGGAGAGCGGCGCCCAGCAAGGCCAGCTCATCCTTACCAAGGAGCACATTGCAAGGTTGGAGGCGGATAAGGAGTTGGGCAAGTTCCACCCCGACTACTGGGGCTGGCAGGGCTTGTTGAAGTCGGGTGCCATCGAGTACCTCGACGCCGAAGAGGAGGAGACGGTCATGATCAGCATGACGCCCGAGGACCTCGACAAGTTCCGTTTCCGCAAAATGGGGTTCATCGTGGAAGACAACTCTGGCCAAGGTAACAACAGGATCAAGACGAAGCCAAACCCGGCTACGCACATGTACACTCACTGCGAGATCCACCCCAGCATGCTGCTCGGCATCTGCGCCAGCATCATCCCCTTCCCCGACCACAACCAAACGCGTCCGGGAAGCT--TTTTTTCTGGGGTCCGATCTACCCCAC-AACATTGGGACGAC-GTCGTTCCACCA---GCTACCC--GACCAC----CCGACCGAC-----GCGAGGGCGGTAGC-GGCGCGACCATGATGACCACGAACACGATGCTGACGCGGCATTTCTATTGCAGGTTCACCTCCAGACCGGCCAATGCGTAAGTTGGATCGATCTTCCGACCG------------CGACCGA----CCGGGTGATGCGGGTGGACTGAC-AACAAGCCCTCTCCAGGGTAACCAAATCGGTGCCGCTTTCTGGTATGTTTCACCA-CGACTGCCA-GCTCTAACGTGTT-GCTAGACCGACAAACTAACTTCCTCTACAGGCAGACCATTTCCGGCGAGCACGGCCTCGACGGCAATGGCGTGTATGTGACTGTCGCCGGGTTACGTCCCACGGAT---GCCTGCTCACCGCGTCGATAGGTACAACGGCAGCTCCGAGCTCCAGCTCGAGCGCATGAACGTCTACTTCAACGAGGTGAGTCGGGATTACCACCTGCT-GCACCAAGCAACC--GCGAGCGCCTTTACTGACAG-CGG---TTACTCAGGCCTCCGGCAACAAGTATGTCCCTCGCGCCGTCCTGGTCGACTTGGAGCCCGGCACCATGGATGCCGTCCGCGCCGGTCCCTTCGGCCAGCTCTTCCGCCCCGACAACTT

>Botryotrichum_foricae_CCF_5752

ATTACAGAGTTGCAAAACT-CCCTAAACCATTGTGAACGTTACCTTCAAACCGTTGCTTCGGCGGGCGGC-CCGGG-----TCC-GCCCGGTGCCCCCTGGCCCCCT----AGCGGGGCGCCCGCCGGAGG-AAACCCAACTCTTGAT-TATTATGGCCTCTCTGAGTCTTCTGTACTGAATAAGTCAAAACTTTCAACAACGGATCTCTTGGTTCTGGCATCGATGAAGAACGCAGCGAAATGCGATAAGTAATGTGAATTGCAGAATTCAGTGAATCATCGAATCTTTGAACGCACATTGCGCCCGCCAGTATTCTGGCGGGCATGCCTGTTCGAGCGTCATTTCAACCATCAAG-CCCCAGGCTTGTGTTGGGGACCTGCGGCTG--CCGCAGGCCCTGAAAACCAGTGGCGGGCTCGCTGT-CACACCGGGCGTAGTAGATTTTATCTCGCT----CAGGGCGTGCTGCGGGTTCCGGCCGTT-AAAAGCCTTTTT------TACCCAA-GGTTGACCTCGGATCAGGTAGGAATACCCGCTGAACTTAAGCATATCAATAAG-CGGAGGAAAAGAAACCAACAGGGATTGCCCCAGTAACGGCGAGTGAAGCGGCAACAGCTCAAATTTGAAATCTGGCTTCGGCCCGAGTTGTAATTTGCAGAGGAAGCTTTAGGCGCGGCACCATCTGAGTCCCCTGGAACGGGGCGCCACAGAGGGTGAGAGCCCCGTATAGATGGACGCCTAGCCTGTGTAAAGCTCCTTCGACGAGTCGAGTAGTTTGGGAATGCTGCTCTAAATGGGAGGTAAATTTCTTCTAAAGCTAAATACCGGCCAGAGACCGATAGCGCACAAGTAGAGTGATCGAAAGATGAAAAGCACTTTGAAAAGAGGGTTAAATAGCACGTGAAATTGTTGAAAGGGAAGCGCTTGTGACCAGACTTGCGCCGGGCGGATCATCCGGTGTTCTCACCGGTGCACTCCGCCCGGCTCAGGCCAGCATCGGTTCTCGTGGGGGGATAAAGGCACCGGGAACGTAGCTCCTCCGGGAGTGTTATAGCCCGGGGCGTAATGCCCTCGCGGGGACCGAGGACCGCGCATCTGCAAGGATG??????????????????????????????????????????????????????????????????????????????????????????????????????????????????????????????????????????????????????????????????????????????????????????????????????????????????????????????????????????????????????????????????????????????????????????????????????????????????????????????????????????????????????????????????????????????????????????????????????????????????????????????????????????????????????????????????????????????????????????????????????????????????????????????????????????????????????????????????????????????????????????????????????????????????????????????????????????????????????????????????????????????????????????????????????????????????????????????????????????????????????????????????????????????????????????????????????????????????????????????????????????????????????????GGTGCTGCTTTCTGGTATGTTCGGCCT-CAACATCAA-GCAATGACGTGTC-AAGACCAAGCAAGACTGATTTCCCCTCCAGGCAGACCATTTCCGGCGAGCACGGCCTCGACAGCAATGGCGTGTATGTGACCGTCGCCGATCCCCGGCCGATGAAT--CCCTCGCTCACCGCTTCGATAGGTACAACGGCACCTCCGAGCTCCAGCTCGAGCGCATGAACGTCTACTTCAACGAGGTGAGTCGGGCCCCGTACGCCT-----TCGGGCAGCCAAACGGCCGTGGTCGCTGACGA-CAAATATGCTGCAGGCGTCCGGCAACAAGTATGTCCCCCGTGCCGTCCTTGTCGACTTGGAGCCCGGCACCATGGATGCCGTCCGCGCCGGTCCTTTCGGCCAGCTCTTCCGCCCCGACAACTT

>Botryotrichum_murorum_DSM_113281

ATTACAGAGTTGCAAAACT-CCC-AAACCATTGTGAACGTTACCTTCAAACCGTTGCTTCGGCGGGCGGC-CCGGG-----TCC-GCCCGGTGCCCCCTGGCCCCCT----CGCGGGGCGCCCGCCGGAGG-AAACCCAACTCTTGATACATTATGGCCTCTCTGAGTCTTCTGTACTGAATAAGTCAAAACTTTCAACAACGGATCTCTTGGTTCTGGCATCGATGAAGAACGCAGCGAAATGCGATAAGTAATGTGAATTGCAGAATTCAGTGAATCATCGAATCTTTGAACGCACATTGCGCCCGCCAGTATTCTGGCGGGCATGCCTGTTCGAGCGTCATTTCAACCATCAAG-CCCCAGGCTTGTGTTGGGGACCTGCGGCTG--CCGCAGGCCCTGAAAACCAGTGGCGGGCTCGCTGT-CACACCGGGCGTAGTAGATTTTATCTCGCT----CAGGGCGTGCTGCGGGTTCCGGCCGTTAAAAAGCCTTTTT------TACCCAA-GGTTGACCTCGGATCAGGTAGGAAGACCCGCTGAACTTAAGCATATCAATAAG-CGGAGGAAAAGAAACCAACAGGGATTGCCCCAGTAACGGCGAGTGAAGCGGCAACAGCTCAAATTTGAAATCTGGCTTCGGCCCGAGTTGTAATTTGCAGAGGAAGCTTTAGGCGCGGCACCATCTGAGTCCCCTGGAACGGGGCGCCACAGAGGGTGAGAGCCCCGTATAGATGGACGCCTAGCCTGTGTAAAGCTCCTTCGACGAGTCGAGTAGTTTGGGAATGCTGCTCTAAATGGGAGGTAAATTTCTTCTAAAGCTAAATACCGGCCAGAGACCGATAGCGCACAAGTAGAGTGATCGAAAGATGAAAAGCACTTTGAAAAGAGGGTTAAATAGCACGTGAAATTGTTGAAAGGGAAGCGCTTGTGACCAGACTTGCGCCGGGCGGATCATCCGGTGTTCTCACCGGTGCACTCCGCCCGGCTCAGGCCAGCATCGGTTCTCGTGGGGGGACAAAGGTCCCGGGAACGTAGCTCCTCCGGGAGTGTTATAGCCCGGGGCGTAATGCCCTCGCGGGGACCGAGGACCGCGCTTCGGCAAGGATGTTGGTCACCCTGGTTCAGGGGCTGCGGAGGAAGAACGTCATCTCGTTCGAGGTTTCGCTCGTCCGCGACATCCGCGACCGCGAGTTCAAGATCTTCTCCGATGCGGGTCGTGTCATGAGGCCGTTGTTCACCGTGGAACAAGAGAAGAAC---GGAGAGAGCGGTGTTGAGATGGGCCAGCTTATTCTCAAAAAGGAGCACATCACAAGGTTGGAGGCGGACAAGGAGTTGGGCAAATATCACCCCGACTACTGGGGCTGGCAGGGCTTGCTGAAGTCGGGTGCTATCGAGTACCTCGACGCCGAGGAGGAGGAGACGGTCATGATCAGCATGACGCCCGAGGACCTCGACAAGTTCCGTTACCGCAAAATGGGGTTCATCGTGGAAGACAACTCTGGCCAAGGTAACAACAGGATCAAGACAAAGCCAAACCCGGCTACGCACATGTACACCCACTGCGAGATCCACCCCAGCATGTTGCTCGGCATCTGCGCCAGCATCATCCCTTTCCCCGACCACAACCAGACGCGTCCGGGAAGCT--TTTTTT-GGGCCCCTGATCTACCCCAC--ACATTGGGACGAC-GTCGTTCCACCA---GCTCCCC--GACCAC----CCGACCGAC-GCGAGCGAGGGCGATAGC-GGCGCTGCCATGATGG-AATGAACACAGTACTGACTTTGTCTTTCTACTACAGGTCCATCTTCAGACCGGCCAATGCGTAAGTGGGATCGACTCGAACACGG------------CGACCGA----CCGGATGGTGCGGGTGGACTGAC-AGCAAG--CTCCCTAGGGTAACCAAATTGGTGCCGCTTTCTGGTATGTTCAGCCT-CAACACCAG-GCACTGACGTGTC-AAGACCGACCAAGACTGACTTCCTCTCCAGGCAGACCATTTCCGGCGAGCACGGCCTCGACAGCAATGGCGTGTACGTGACGGGCGCCGACTCCCGACCAATGATC--CCCTCGCTCACCGATTCGATAGGTACAACGGCACCTCGGAGCTCCAGCTCGAGCGCATGAACGTCTACTTCAACGAGGTGAGTCGGACACATGCAGCCTT--CATCAGGCAACCGAACGTTCCTGGTTGCTGACAA-CAGAT-TTCTACAGGCCGCCGGCAACAAGTATGTTCCCCGCGCCGTCCTGGTCGACTTGGAGCCGGGCACCATGGATGCCGTCCGCGCCGGTCCCTTCGGCCAGCTCTTCCGCCCCGAC?????

>Chaetomium_globosum_CBS_160_62

ATTACAGAGTTGCAAAACT-CCCTAAACCATTGTGAACGTTACCT--ATACCGTTGCTTCGGCGGGCGGCCCCGGGGTTTACCC-CCCGGGCGCCCCTGGGCCCCAC-----CGCGGGCGCCCGCCGGAGG-TCACCAAACTCTTGATAATTTATGGCCTCTCTGAGTCTTCTGTACTGAATAAGTCAAAACTTTCAACAACGGATCTCTTGGTTCTGGCATCGATGAAGAACGCAGCGAAATGCGATAAGTAATGTGAATTGCAGAATTCAGTGAATCATCGAATCTTTGAACGCACATTGCGCCCGCCAGCATTCTGGCGGGCATGCCTGTTCGAGCGTCATTTCAACCATCAAGCCCCCGGGCTTGTGTTGGGGACCTGCGGCTG--CCGCAGGCCCTGAAAAGCAGTGGCGGGCTCGCTGT-CGCACCGAGCGTAGTAGCATACATCTCGCT----CTGGTCGCGCCGCGGGTTCCGGCCGTTAAACCACC-TTTT------AACCCAA-GGTTGACCTCGGATCAGGTAGGAAGACCCGCTGAACTTAAGCATATCAATAAG-CGGAGGAAAAGAAACCAACAGGGATTGCCCTAGTAACGGCGAGTGAAGCGGCAACAGCTCAAATTTGAAATCTGGCTTCGGCCCGAGTTGTAATTTGCAGAGGAAGCTTTAGGCGCGGCACCTTCTGAGTCCCCTGGAACGGGGCGCCATAGAGGGTGAGAGCCCCGTATAGTTGGATGCCTAGCCTGTGTAAAGCTCCTTCGACGAGTCGAGTAGTTTGGGAATGCTGCTCAAAATGGGAGGTAAATTTCTTCTAAAGCTAAATACCGGCCAGAGACCGATAGCGCACAAGTAGAGTGATCGAAAGATGAAAAGCACTTTGAAAAGAGGGTTAAATAGCACGTGAAATTGTTGAAAGGGAAGCGCTTGTGACCAGACTTGCGCCGGGCGGATCATCCGGTGTTCTCACCGGTGCACTCCGCCCGGCTCAGGCCAGCATCGGTTCTCGCGGGGGGATAAAGGTCCTGGGAACGTAGCTCCTCCGGGAGTGTTATAGCCCGGGGCGTAATGCCCTCGCGGGGACCGAGGTTCGCGCATCTGCAAGGATGCTTGTCACGCTGGTTCAGGGGCTGCGGAGAAAGAACGTTATCTCGTTTGAGGTTTCGCTCGTTAGAGACATCCGCGACCGTGAGTTCAAGATCTTTTCAGATGCGGGTCGGGTGATGAGGCCGCTGTTCACGGTGGAGCAAGAACCGAAT---GGCGAGAGCGGCGCTGAGATGGGCGCACTGATCCTGAACAAGGATCATATTGGGCGCCTGAAGATGGACGCAGAGCTGGGCAAATACCACCCGGACTACTGGGGCTGGCAAGGCCTGTTGAAGTCGGGCGCTATTGAGTATCTTGATGCTGAGGAGGAGGAGACGGTCATGATCTGCATGACCCCCCAGGATCTTGATCAGTTCCGTGCCCGCAAGATGGGAAGGATCGAGCCGGACAACTCCGGGTTGGGCAATAACCGGATCAAGACGAAACCAAATCCGACAACTCACATGTACACGCACTGCGAGATCCATCCGAGCATGCTCCTCGGCATCTGCGCAAGCATCATCCCCTTTCCTGATCATAACCAACTGCGTCTGGGAAGCTTTTTTTTT-GGGCCTCTGAACTACCCCAC--TCATCGGGACGACCCTCATTTCCACA---GCTCCT-------------------------------GACGGATAGC-AACACGACGATGATGCGAAGCTTGATGATGCTGACTGCTTTTTCGTACTATAGGTTCACCTCCAGACCGGCCAGTGCGTAAGTTGGACCGAATTGAACATTA------------CGACCGA----CCGG-CCGCGCAGGATAACTGAC-ATGGAG--CTCTCTAGGGTAACCAAATCGGTGCCGCTTTCTGGTACGTCCAAGCA-AAGCAAACA-CTCTTG----GCT-GATGACAATCGAGACTGACTT-CTTTTCAGGCAGACCATCTCTGGCGAGCACGGCCTCGACAGCAATGGCGTGTATGTGGGCAT-GACAGTTCCCAACCGATAAAT---CCCCGCTCACCGCTTCGATAGGTACAACGGCACCTCCGAGCTCCAGCTCGAGCGTATGAACGTGTACTTCAACGAGGTCAGTCGGGTCAAATAATTTT-------ACACGACC-GA-----GTGATGGCGTGCTC-ATAGTATTATACAGGCTTCCGGCAACAAGTATGTTCCTCGCGCTGTCCTCGTCGACTTGGAGCCCGGCACCATGGATGCCGTCCGTGCCGGCCCCTTCGGCCAGCTCTTCCGCCCGGACAACTT

>Dichotomopilus_pseudofunicola_CBS_142033

ATTACAGAGTTGCAAAACT-CCC-AAACCATCGTGAACGTTACCT--ACATCGTTGCTTCGGCGGGTGGC---GGG----CTTCGGCCC--TGCCC-TCGGCCCCTC------TCGGGGGCCCGCCGGAGGTACACCAAACTCTTGAATTTACATGGCCTCTCTGAGTCTTCTGTACTGAATAAGTCAAAACTTTCAACAACGGATCTCTTGGTTCTGGCATCGATGAAGAACGCAGCGAAATGCGATAAGTAATGTGAATTGCAGAATTCAGTGAATCATCGAATCTTTGAACGCACATTGCGCCCGCCAGTATTCTGGCGGGCATGCCTGTCCGAGCGTCATTTCAACCATCAAG-CCCCCGGCTTGTGTTGGGGACCTGCGGCACACCCGCAGGCCCTGAAAACCAGTGGCGGGCTCGCTGTCCACACCGAGCGTAGTAGCATATCTTTGTCTCGCTCAGGGCGTGCGGCGGGTTCCGGCCGTGAAACCCACCTTCTCGAAGGTACCCAAAGGTTGACCTCGGATCAGGTAGGAAGACCCGCTGAACTTAAGCATATCAATAAG-CGGAGGAAAAGAAACCAACAGGGATTGCCCTAGTAACGGCGAGTGAAGCGGCAACAGCTCAAATTTGAAATCTGGCTTCGGCCCGAGTTGTAATTTGCAGAGGAAGCTTTAGGCGCGGCACCTTCTGAGTCCCCTGGAACGGGGCGCCATAGAGGGTGAGAGCCCCGTATAGTTGGATGCCTAGCCTGTGTAAAGCTCCTTCGACGAGTCGAGTAGTTTGGGAATGCTGCTCAAAATGGGAGGTAAATTTCTTCTAAAGCTAAATACCGGCCAGAGACCGATAGCGCACAAGTAGAGTGATCGAAAGATGAAAAGCACTTTGAAAAGAGGGTTAAACAGCACGTGAAATTGTTGAAAGGGAAGCGCTTGTGACCAGACTTGCGCCAGGCTGATCATCCGGTGTTCTCACCGGTGCACTCGGCCCGGCACAGGCCAGCATCGGTTCTCGCGGGGGGATAAAGGTCTCGGGAACGTAGCTCCTCCGGGAGTGTTATAGCCCGGGGCGTAATGCCCTCGCGGGGACCGAGGTTCGCGCATCTGCAAGGAT?CTGGTTACCTTGGTCCAAGGCTTACGAAGGAGGAACGTCATCTCCTTCGAGGTGTCGCTTGTCCGCGATATCCGCGACCGCGAGTTCAAGATCTTTTCCGATGCGGGGCGTGTCATGAGACCGCTATTCACCGTGGAGCAAGAAGTAAACGGCGGCGAGAGCGGAGCAGAGATGGGCGCTTTGATCCTCAACAAGGAGCACATTGCGCGGTTGGAAACGGACAAAGACCTCGGCCGGTACCACCCAGACTACTGGGGTTGGAGAGGCTTGTTGAAGTCGGGTGCGATTGAGTACCTTGATGCCGAAGAAGAGGAGACAGTCATGATCTGCATGACTCCCGAAGACCTGGAGCGATTCCGCTTGCGGAAGAAGGGGAGAGAAATGCCCGACAACTCCGGGGTGGGCAACAATCGGATCAAGACGAAGACCAACCCGACAACTCACATGTACACACACTGCGAGATCCACCCCAGTATGCTGCTTGGCATTTGCGCCAGCATCATTCCGTTCCCCGACCACAACCAAACGCGTCGAGGAAGCTTTTTTTTAGGCTCCTGGAATCTACCCCACCCAATTTGAGACGACCTCCTCCGCAAAGC--GCTCCC---GACCGATGAACCGACGGAT---------GCTGGCGAGC-AACGCGGCTATGATGGGAAGAT-CTCGATGCTAACTTGACTTTTCTACTACAGGTCCACCTTCAGACCGGCCAGTGCGTAAGTTGGAACCGAATAGATATCG------------CTGCCGA----CCGACAAGGCACATTGACTGACCTTACCCCCTCTTTATAGGGTAACCAAATTGGTGCCGCCTTCTGGTAAGTTGTAGCCGAAACCAGGACGAGTTTTTACATT-CAGAAC-ATCAGTACTAACTTCATATCCAGGCAAACCATCTCTGGCGAGCACGGCCTCGATAGCAATGGCGTGTATGTAGATATCGCGAGCCCCCGACCGATTCGT--ACCCCGCTCACCGCTTCAATAGGTACAACGGCACTTCCGAGCTCCAGCTGGAGCGCATGAACGTCTACTTCAACGAGGTAGGTTTGGTGGTCTATC---------GTGATGGGGTCAAGTGATGGCATGCTGATAAGTCCCCCTACTTCAGGCCTCCGGCAACAAGTATGTTCCCCGTGCCGTCCTTGTCGACTTGGAGCCCGGCACTATGGACGCTGTCCGCGCCGGTCCCTTCGGCCAGCTTTTCCGCCCGGACAACTT
